# Canonical and Interior Circular RNAs Function as Competing Endogenous RNAs in Psoriatic Skin

**DOI:** 10.3390/ijms22105182

**Published:** 2021-05-13

**Authors:** Xiaoxin Liu, Jacqueline Frost, Anne Bowcock, Weixiong Zhang

**Affiliations:** 1 Institute for Systems Biology, Jianghan University, Wuhan 430056, China; xiaoxinliu@wustl.edu; 2 Department of Computer Science and Engineering, Washington University, Saint Louis, MO 63130, USA; 3 Department of Oncological Sciences, Icahn School of Medicine at Mount Sinai, New York, NY 10029, USA; jacqueline.frost@mssm.edu (J.F.); anne.bowcock@mssm.edu (A.B.); 4 Departments of Dermatology and Genetics & Genomics, Icahn School of Medicine at Mount Sinai, New York, NY 10029, USA; 5 Department of Genetics, Washington University School of Medicine, Saint Louis, MO 63130, USA

**Keywords:** circular RNA, competing endogenous RNA, psoriasis, non-coding RNA

## Abstract

(1) Background: Understanding the function of circular RNAs (circRNAs), a class of noncoding RNA, in psoriatic skin can provide important insights into the complex regulation of genes contributing to the pathogenesis of psoriasis. (2) Methods: A novel method was applied to RNA-seq datasets from 93 skin biopsy samples to comprehensively identify circRNAs of all types, i.e., canonical circRNAs from the intron-exon junctions of mRNAs and interior circRNAs (i-circRNAs) from the interior regions of exons, introns, and intergenic regions. Selected circRNAs were experimentally validated by qRT-PCR and Sanger sequencing. CircRNAs with abundant and differential expression were identified and their putative function as competing endogenous RNAs (ceRNAs) was analyzed by an integrated analysis of circRNAs, microRNAs, and mRNAs. (3) Results: With a comprehensive search using no information of splicing signals, we systematically identified 179 highly abundant circRNAs in psoriatic skin. Many of these were reported for the first time and many were differentially expressed in involved versus normal or uninvolved skin. Validation based on three additional RNA-seq datasets confirmed most of the identified circRNAs in psoriatic skin. Experimental analyses confirmed the expression of the well-known circRNA CDR1as, a canonical circRNA, and a novel i-circRNA in psoriasis. We also identified many circRNAs that may act as ceRNAs to regulate the expression of mRNA genes in psoriasis-related signaling pathways in psoriasis. (4) Conclusions: The result of the study suggested that circRNAs are abundant in psoriatic skin, have distinct characteristics, and contribute to psoriatic pathogenesis.

## 1. Introduction

Psoriasis (PS) is a chronic, inflammatory, and immune-mediated skin disease, characterized by raised, red scaly plaques [1]. Its prevalence ranges from between 0.09% and 11.4%, affecting at least 100 million individuals worldwide [2]. Besides the long-lasting and high recurrence rate, PS may increase the risk of stroke [3], myocardial infarction [4], type 2 diabetes [5], metabolic syndrome [6,7], and cancer [8,9]. The etiology and pathogenesis of PS remain poorly understood. Studies in the past decades have identified many genetic risk factors [10,11] and aberrant expression of many transcripts including non-coding RNAs such as microRNAs (miRNAs) [12,13,14,15] and long non-coding RNAs (lncRNAs) [16]. The expression of many PS related mRNAs may be regulated by miRNAs [14,15]. For example, miR-21 contributes to T-cell derived psoriatic skin inflammation [17], and miR-31 overexpression enhances the production of inflammatory cytokines and chemokines [18]. Many lncRNAs also function in PS, e.g., PRINS [19,20], lnc-IL7R [21], and LincR-Ccr2-5′AS [22].

Circular RNAs (circRNAs), a new class of non-coding RNAs, have been identified in nearly all eukaryotic species [23,24,25,26]. CircRNAs have many molecular functions [27]. For example, they act as sponges of miRNAs [28], promote transcription [29], alter the expression of mRNAs [30,31] and proteins [32], and can even turn into functional polypeptides [33,34,35]. Many circRNAs exhibit aberrant expression in complex diseases such as Alzheimer’s disease (AD) [36,37], osteoarthritis [38], and cardiovascular disease [39,40] as well as cancer [41,42].

However, the expression and potential function of circRNAs in PS are understudied and poorly understood. Three previous studies have been reported: A microarray-based analysis of psoriatic lesions identified 4,956 differentially expressed (DE) circRNAs [43]; a study of mesenchymal stem cells (MSC) of psoriatic skin described 129 DE circRNAs [44]; and a recent study of six paired lesional and non-lesional skin samples reported 148 DE circRNAs [45]. The microarray study missed novel circRNAs, the MSC study was unlikely to have captured many circRNAs in psoriatic skin, and the skin study compared circRNAs between lesional and non-lesional skin but not those of healthy controls.

We set forth to identify novel circRNAs and study their potential functions in PS. We studied a cohort of 93 skin tissue samples from which a large collection of circRNA-enriched RNA-seq libraries from 28 psoriatic-involved (PP) lesions, 38 normal (NN) controls, and 27 psoriatic-uninvolved (PN) skin samples were derived. Besides studying circRNAs arising from intron-exon boundaries, we also studied interior circRNAs (i-circRNAs) arising from the interior regions of introns, exons, and intergenic regions [46]. We confirmed our discoveries with three validation sets of RNA-seq data from psoriatic skin samples and cell lines. We also experimentally validated and studied three circRNAs in psoriatic skin with qRT-PCR and Sanger sequencing. Furthermore, we studied the potential function of circRNAs as competing endogenous RNAs (ceRNAs) in PS.

## 2. Results

### 2.1. Detection and Profiling of circRNAs in PS

**Figure 1:**
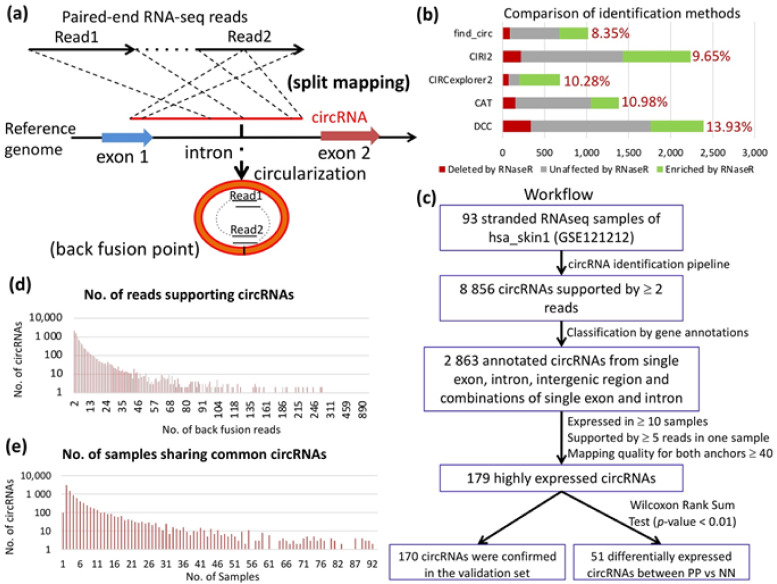
Workflow of CAT and analysis of circRNAs in PS. (**a**) Illustration of split mapping of paired-end RNA-seq reads to candidate circRNAs. If Read1 is completely mapped and Read2 is split mapped to the reference genome, a circular structured RNA, circRNA, could be formed from this genomic locus with the back-fusion point identified from the split mapping. (**b**) Stacked bar plots of the circRNAs in HeLa cells (RNase R vs. control) that were predicted by five circular RNA identification methods, with results separated into RNase R resistant (≥ fivefold enrichment, green), unaffected (one to fivefold enrichment, gray) and RNase R sensitive (depleted in RNase R treated samples, red). Percentages refer to the fraction of RNase R sensitive circRNAs defined as false positives. The minimal number of supporting reads was set to 3 for all five methods compared. (**c**) Workflow for identification and analysis of circRNAs in the ribo-zero, stranded paired-end RNA-seq data from 93 PS samples. In total, 8856 unique circRNAs were identified and 2863 circRNAs were annotated and classified by single exons, single introns, pairs of adjacent exons and introns, and intergenic non-coding transcripts. A total of 179 highly expressed circRNAs were selected by three criteria, i.e., expressed in more than 10 samples, supported by more than five reads in at least one sample and filtered based on a mapping quality threshold of 40 for both anchors. This resulted in 51 differentially expressed circRNAs when PP vs. NN skin was compared by Wilcoxon rank sum test. (**d**) The number of BF reads supporting 8856 unique circRNAs, and (**e**) the number of samples expressing 8856 unique circRNAs, where the y-axes are logarithmic (log10).

A new method for finding circRNAs of all types, termed CAT, was developed to comprehensively identify circRNAs and i-circRNAs from RNA-seq data (Figure 1a, see Methods and an early version of CAT in [46]). CAT uses no information of splicing signals or genome annotation for circRNA detection but uses genome annotation to classify circRNAs into the categories of canonical circRNAs with two back-fusion (BF) points at intron-exon boundaries, complete i-circRNAs with both BF points not residing at intron-exon boundaries, and partial i-circRNAs with one BF point from an intron-exon boundary [46]. When applied to paired-end RNA-seq data, it detects a circRNA if one end of a paired-end read is mapped to the genome in an orientation-reversed splitting form (see Methods); the splitting points define the back-splicing or BF point of the circRNA (Figure 1a). Although a circRNA may span more than one exon or intron, it is unreliable to infer its actual structure based on RNA-seq data alone, particularly if RNA-seq data were not enriched for circRNAs. Therefore, we focused on candidate circRNAs from single exons, single introns, pairs of adjacent exons and introns, and intergenic transcripts. We compared CAT with the four most popular existing methods for circRNA detection, i.e., CIRI2 [47], CIRCexplorer2 [48], DCC [49], and find_circ [23], on a single-end stranded HeLa dataset [50] following the scheme proposed in [51]. CAT had a comparable performance with the other four methods, i.e., the estimated error rate of CAT was ~3% lower than that of DCC and ~2% higher than that of find_circ (Figure 1b). In summary, CAT is an exclusively RNA-seq data-driven approach for identifying circRNAs from any genomic locus without using any information of splicing signals or genome annotation.

Using CAT, we detected and characterized circRNAs in 93 biopsy skin samples for circRNA discovery (Figure 1c and Supplementary Figure S1a, Table S1). We identified 8856 circRNAs supported by at least two sequencing reads (Figure 1c–e). To increase the confidence of reporting genuine circRNAs, we focused on 2863 circRNAs originating from single exons, single introns, pair of adjacent exons and introns, and intergenic transcripts. As expected, splicing signals were observed on most canonical circRNAs arising from intron-exon boundaries but not on complete i-circRNAs (Figure S1b). In total, 91.69% (739/806) and 72.65% (263/362) of the canonical and partial boundary circRNAs were accompanied by a splicing signal, whereas this ratio decreased to 11.98% (203/1,695) for complete i-circRNAs. Combined, the results showed that i-circRNAs existed broadly in skin tissues and that most i-circRNAs were not accompanied by the splicing signal.

### 2.2. circRNAs in PS and their Characteristics

**Figure 2:**
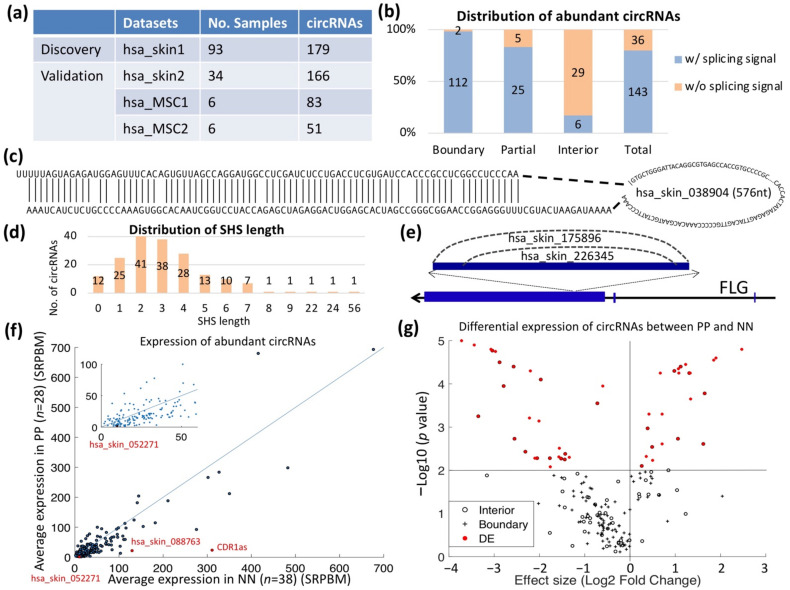
Distribution and characteristics of abundant circRNAs. (**a**) Names and number of samples in the discovery and validation datasets. In total, 179 abundant circRNAs were identified in the discovery dataset, among which 166, 83, and 51 were independently validated by the three validation datasets. (**b**) Distribution of the 179 circRNAs in the discovery datasets. Boundary circRNAs are canonical circRNAs, and i-circRNAs are further classified into partial and complete i-circRNAs. All circRNAs are further annotated as to whether they are adjacent to splicing signals. (**c**) An example of RNA folding structure and complementary sequences flanking an exon i-circRNA, hsa_skin_038904. (**d**) Distribution of the lengths of short homologous sequences (SHS). (**e**) An example of two circRNAs, hsa_skin_175896 and hsa_skin_226345, arising from FLG (filaggrin). (**f**) Average expression (spliced reads per billion mappings [SRPBM]; see Methods) of the 179 highly expressed circRNAs in PP and NN skin. (**g**) Volcano plot visualizing differential expression of circRNAs when PP and NN skin samples were compared. The red and black dots in the plot represent significantly differentially expressed (*p*-value < 0.01, see Methods) and not significantly expressed circRNAs, respectively. Circle and cross marks represent interior and boundary circRNAs, respectively.

We identified 179 circRNAs expressed in at least 10 samples and supported by at least five reads in one of these samples (Figure 1c, Table S2). Among the 179 circRNAs, 64 (35.8%) were identified for the first time and 65 (36.3%) were i-circRNAs, 49 of which were novel. As a validation of the result, we examined the expression of the 179 circRNAs in three additional RNA-seq datasets (Figure 2a). In total, 170 circRNAs were expressed in at least one of the three validation datasets. In particular, 166 (92.7%) of the 179 circRNAs were expressed in another large RNA-seq dataset of 34 psoriatic and normal skin biopsy samples in dataset hsa_skin2, and 37 circRNAs were highly expressed in all three validation datasets. Among these, three were exon or intron i-circRNAs with no associated splicing signal (Figure 2a). A substantial portion of the boundary and partial i-circRNAs, but very few of the complete i-circRNAs, of the 179 circRNAs carried the splicing signal (Figure 2b).

Many circRNAs have two features: (1) they have complementary sequences flanking their BF points. Twenty-four circRNAs had paired flanking sequences, among which 17 were i-circRNAs including 15 with no splicing signal (see Figure 2c for an example). This suggested that the production of these i-circRNAs may be assisted by fold-back structures of complementary sequences; and (2) the BF points of many circRNAs, particularly i-circRNAs, resided within short homologous sequences (SHS), with lengths of 2- to 56-nt and peaked at 2-nt (Figure 2d). Among the 179 abundant circRNAs, 101 (56.4%) were associated with SHS that were longer than 2-nt, 29 of which had no splicing signal (Table S2), indicating a potential role of SHS in circRNA biogenesis [46].

More than one circRNA may arise from a genomic locus. Among the 161 host genes of the 179 circRNAs, 13 (including FLG, FLG2, KRT10, KRT2, and LPP; Table S2) produced multiple circRNAs. For example, hsa_skin_175896 contained hsa_skin_226345 (Figure 2e).

### 2.3. Aberrantly Expressed circRNAs in PS

The digital expression level of a circRNA was quantified by the number of reads splitting mapped to its BF point. The digital expression was normalized to the reads per billion mappings (SRPBM; see Methods). Using SRPBM, 47 (26.3%) of the 179 circRNAs were expressed at higher levels between PP vs. NN (Figure 2f) and 51 (28.5%) circRNAs were differentially expressed (DE) (see Methods) in PP vs. NN skin (Figure 2g).

A putative function of circRNAs is acting as competing endogenous RNAs (ceRNAs) [52] for regulating mRNA genes that share common regulating miRNAs with circRNAs. We named these circRNAs ce-circRNAs and the associated mRNAs circRNA-associated genes. There were 8096 mRNA genes significantly DE between PP vs. NN skin (Table S3), among which 4034 were associated with 51 DE circRNAs.

**Figure 3:**
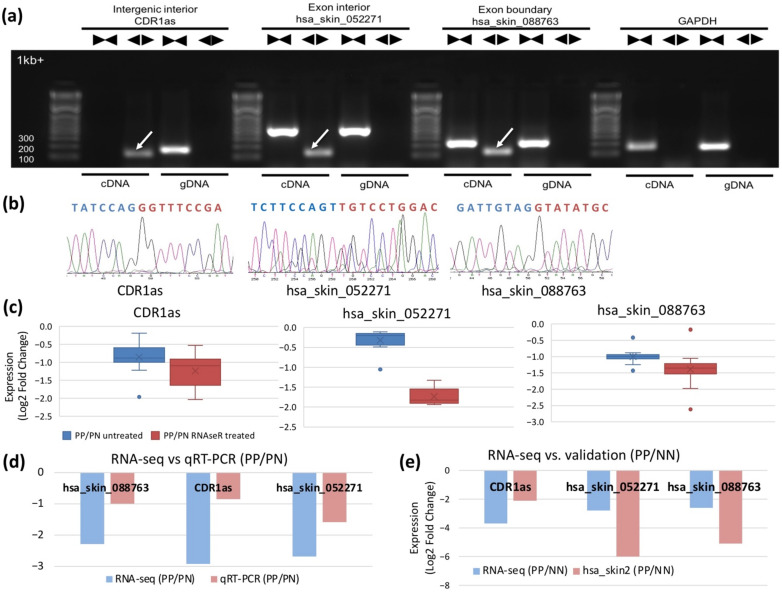
Experimental validation of three identified circRNAs in psoriatic skin and HaCaT keratinocyte cells. (**a**) Validation of a well-known intergenic i-circRNA (CDR1as), a novel exon i-circRNA (hsa_skin_052271), and a canonical circRNA (hsa_skin_088763) by PCR. The divergent and convergent arrows above the gel image represent, respectively, the divergent and convergent PCR primers used in RNA (cDNA) and DNA (gDNA), and the white arrows in the gel image point to circRNAs. Numbers on the left ranging from 100 to 1kb+ represent sizes in DNA ladder. GAPDH was used as an internal control. (**b**) Validation of CDR1as, hsa_skin_052271, and hsa_skin_088763 by Sanger sequencing. (**c**) Box and whisker plot for RNase R validation of differential expression of CDR1as, hsa_skin_052271, and hsa_skin_088763 when PP vs. PN skin was compared. (**d**) Quantitative real-time PCR (qRT-PCR) validation of differentially expressed circRNAs hsa_skin_088763, CDR1as, and hsa_skin_052271 in PP vs. PN skin. (**e**) RNA-seq (hsa_skin2 of validation set) validation of differentially expressed circRNAs CDR1as, hsa_skin_052271, and hsa_skin_088763 in PP vs. NN skin.

From these circRNAs, we selected two canonical circRNAs, hsa_skin_194345 (CDR1as) and hsa_skin_088763 (hsa_circ_0109327), and three i-circRNAs, hsa_skin_100269, hsa_skin_143837, and hsa_skin_052271 for experimental validation using psoriatic skin biopsy samples (PP and PN) and HaCaT keratinocyte cells (see Methods). Using sequencing reads across BF points as surrogates to circRNAs, the five circRNAs had 1,440, 405, 41, 24, and 22 reads in the discovery dataset, respectively. Note that these five circRNAs were all detected in the validation datasets, providing the first validation of these circRNAs in psoriasis. For experimental validation, divergent and convergent PCR primers (Table S4) were applied separately to the RNA and DNA of these circRNAs. Three circRNAs, CDR1as, hsa_skin_052271, and hsa_skin_088763, were experimentally validated (Figure 3a) and their BF points were confirmed with Sanger sequencing (Figure 3b). Sanger sequencing successfully recovered the full-length of hsa_skin_052271. Furthermore, the three validated circRNAs were subjected to RNase R treatment (Figure S2a) and the changes in their expression in PP vs. PN were confirmed. The fold changes from the RNase R experiments were consistent with and even more pronounced than those of the untreated ones (Figure 3c and Supplementary Figure S2b). qRT-PCR was also applied to validate their RNA-seq based DE in PP vs. PN skin and consistent fold change directions were confirmed (Figure 3d and Supplementary Figure S2c). We also determined that these circRNAs were DE in PP vs. NN skin by an analysis of sequencing data from the hsa_skin2 validation dataset (Figure 3e).

### 2.4. Putative circRNA Functions in PS

**Figure 4:**
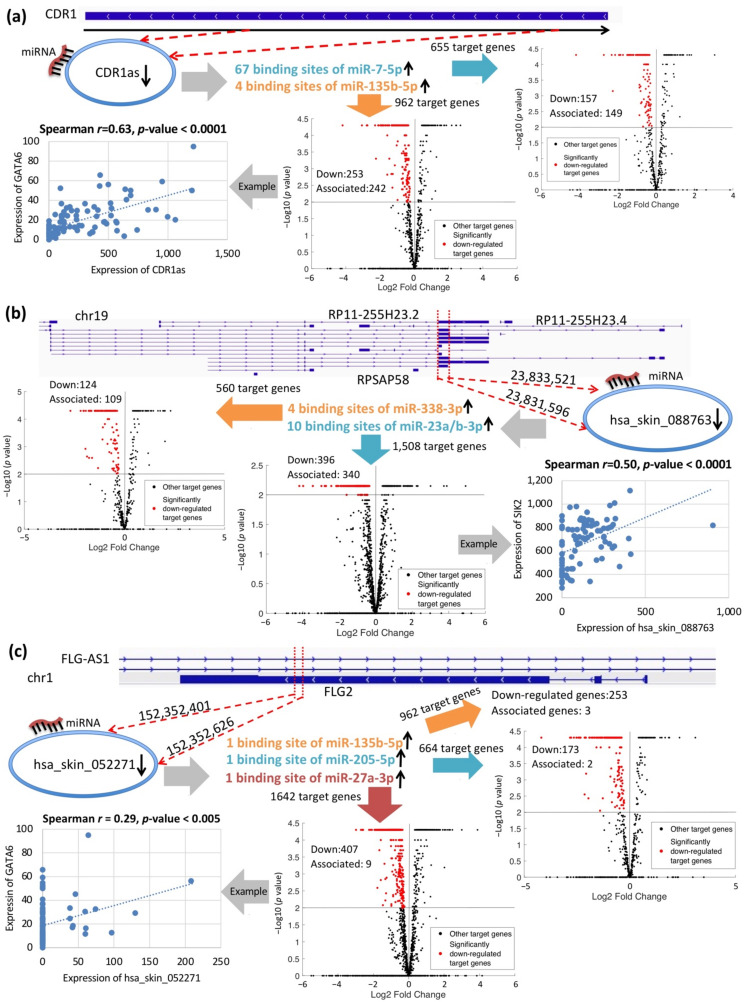
Putative functions of three circRNAs as ceRNAs. The genomic origin of (**a**) intergenic i-circRNA CDR1as, (**b**) exon boundary circRNA hsa_skin_088763, and (**c**) exon i-circRNA hsa_skin_052271, their psoriasis-related binding miRNAs predicted by miRanda, and their circRNA-associated genes. Volcano plots visualize the differential expression of target genes between PP and NN skin. The red and black dots in the plot represent significantly down-regulated (*p*-value < 0.01, see Methods) and other target genes, respectively. Among the significantly down-regulated target genes, those that are positively correlated with the expression of circRNAs are defined as circRNA-associated genes (see Methods). Scatter plots show Spearman’s rank-order correlation between three circRNAs and one of their associated genes, where *r* is the Spearman correlation coefficient.

The three experimentally validated circRNAs may potentially function as ce-circRNAs in psoriatic skin. CDR1as may regulate 149 out of 655 targets of miR-7-5p via 67 binding sites and 242 out of 962 targets of miR-135b-5p via four binding sites (Figure 4a, Table S5). CDR1as was sharply down-regulated 3.7-fold between PP vs. NN (Table S6), whereas both miR-7-5p and miR-135b-5p are up-regulated in psoriasis [12,15,53]. The target genes that were down-regulated in PP skin and positively correlated with the expression of CDR1as (Spearman’s rank-order correlation analysis) were called circRNA-associated genes of CDR1as (see Methods, Figure 4a). Among the associated genes were EGR3, GATA6, GATA3, and FOXN3, which play important roles in psoriasis. EGR3 can regulate late epidermal differentiation and contribute to the keratinocyte differentiation-related module in a skin-specific network [54]. GATA6 has significantly lower expression in psoriatic dermal MSCs [55]. FOXN3, regulating cell differentiation and cell cycle, is down-regulated along with GATA3 in psoriasis [56].

CircRNA hsa_skin_088763 from pseudogene RP11-255H23.2 may regulate 109 out of 560 targets of miR-338-3p via four binding sites and 340 out of 1,508 targets of miR-23a/b-3p via 10/11 binding sites (Figure 4b, Table S5). hsa_skin_088763 was down-regulated 2.6-fold when PP vs. NN was compared (Table S6), and miR-338-3p and miR-23a/b-3p were up-regulated in psoriasis, consistent with this trend in their putative regulatory circRNA [57]. Several psoriasis-related genes were among the associated genes of hsa_skin_088763, including GATA6, SIK2 (Figure 4b), IL17RD [58], EGR3, FAS, LRIG1, and PPARGC1A [59]. LRIG1 negatively regulates growth factor signaling to regulate epidermal stem cell quiescence [60]; SIK2 modulates cytokine responses during innate immune activation [61]; FAS signaling is essential for inducing key inflammatory cytokines in psoriasis [62]. All these results indicated that hsa_skin_088763 functions as a ceRNA in psoriasis.

hsa_skin_052271, an i-circRNA from the third exon of gene FLG2, may mediate three target genes of miR-135b-5p, two target genes of miR-205-5p, and nine target genes of miR-27a-3p (Figure 4c, Table S5). Hsa_skin_052271 was down-regulated 2.8-fold between PP and NN (Table S6), and miR-135b-5p [12,15,53], miR-205-5p [12] and miR-27a-3p [57] were up-regulated in psoriasis. Psoriasis-related gene GATA6 was an associated gene of hsa_skin_052271 (Figure 4c).

We performed GO and KEGG pathway analyses separately on the 1,558 up-regulated and 2,476 down-regulated circRNA-associated transcripts (see Methods). The results revealed that most of the top 20 KEGG pathways and many of the top 20 GO terms were psoriasis-related (Figures S3–S6). The above pathway analysis added additional pieces of evidence that CDR1as, hsa_skin_088763, and hsa_skin_052271 may play essential roles in psoriatic pathogenesis by acting as ceRNAs.

## 3. Discussion

CircRNA has not been well-investigated in psoriasis. The only previous study on this species of RNA in psoriatic lesional skin used a small cohort of six patients but no healthy controls [45]. That study revealed that circRNAs in psoriatic skin exhibited a lower abundance in lesional skin than in non-lesional skin, which was also observed in our current study (Figure 2f,g). That study also reported that miRNAs were in large part not responsive to the differential expression of circRNAs because the miRNAs targeted by circRNAs were profiled as a group so that the regulatory relationships of pairs of circRNAs and miRNAs were lost.

In the study described here, we leveraged a large collection of RNA-seq data from psoriatic and normal skin biopsies to detect and profile the expression of circRNAs and identify their potential targets contributing to PS. Among the 179 highly abundant circRNAs detected, 64 (35.8%) were identified the first time, and 51 (28.5%) exhibited aberrant expression in PS. Interior circRNAs (i-circRNAs), a newly characterized type of circRNA [46], were also detected in PS. Specifically, 65 (36.3%) of the 179 circRNAs were i-circRNAs and 49 of the 65 i-circRNAs were novel.

We validated most of the identified circRNAs and i-circRNAs with three new validation RNA-seq datasets and experimentally confirmed and studied the expression of three circRNAs (CDR1as, hsa_skin_088763, and hsa_skin_052271) in PS by PCR and Sanger sequencing (Figure 3, Figure S2).

The significance of this experimental confirmation is threefold. First, it demonstrated for the first time that the most well-studied circRNA CDR1as can potentially function in psoriasis. CDR1as is known to play a role in cancer [63,64], neurological diseases such as Alzheimer’s disease [36,37], and many other diseases, and during development [65]. CDR1as was the fourth most abundant and the topmost DE circRNA in psoriatic skin (Tables S2 and S6). It was not only aberrantly expressed but also functioned as a ce-RNA [31,52] through a circRNA-miRNA-mRNA regulatory cascade by regulating miR-7-5p and miR-135b-5p in psoriasis. Psoriasis is an inflammatory skin disorder where hyperproliferation of keratinocytes is accompanied by abnormal differentiation of immune cells [66]. The theme of abnormal cell proliferation that is common in psoriasis and cancer explains, in part, the aberrant expression of CDR1as in these different diseases. Indeed, our results suggest that many aberrantly expressed circRNAs regulated many genes function in cancer-related pathways (Figures S3–S5). miR-135b-5p is also reported to target KLF4 [67], an important transcription factor in skin barrier formation [68]; a pathway that is disrupted in psoriasis.

The second significance of this experimental confirmation is that it showed, for the first time, that interior circRNA (i-circRNA) exist and potentially function in human cells [46]. The novel i-circRNA hsa_skin_052271 emerged from an exon and its BF points were not adjacent to the splicing signal. Importantly, hsa_skin_052271 may function as a ce-RNA, mechanistically acting as a sponge of miR-27a-3p and thus positively regulating the expression of GATA6, which is a target of miR-27a-3p and down-regulated significantly in psoriatic dermal MSCs [55].

The third is that the result revealed a possible connection between a circRNA and a pseudogene. CircRNA hsa_skin_088763 arose from pseudogene RP11-255H23.2. Many pseudogenes are known to function as ceRNAs as they are prone to become the targets of miRNAs [52]. Therefore, circRNAs and pseudogenes may crosstalk by competing for miRNA binding. This is likely the case in our study since both hsa_skin_088763 and pseudogene RP11-255H23.2 are down-regulated in psoriatic lesions (Tables S2 and S3).

The i-circRNAs reported in the current study were genuine circRNAs, which was supported by the validation datasets. i-circRNAs were previously identified from intergenic transcripts in rice [69] and oncogenic chimeric transcripts in cancer [70]. We previously reported a large number of i-circRNAs in humans, mice, and rice [46]. The results from the current study expanded our understanding of i-circRNAs in complex disease.

The discovery of i-circRNAs was thought-provoking and shed new light on the biogenesis of circRNAs. While the splicesome-based biogenesis model is well-supported, it cannot explain all canonical circRNAs. In particular, 19,444 (21.05%) of the 92,369 circRNAs in circBase have no splicing signal adjacent to their back splicing points. Interestingly, the back fusion points of most i-circRNAs and some canonical circRNAs were embedded in short homolog sequences (SHS, Figure 2d) [46]. We hypothesized earlier that i-circRNAs and canonical circRNAs with SHS are products of RNA polymerase template switching during RNA processing [46]. This needs to be tested in future studies.

## 4. Materials and Methods

### 4.1. RNA-seq Data

Four paired-end RNA-seq datasets from human skin were used to investigate circRNAs in PS (Figure S1a, Table S1). One was used for discovery, the other three were used for validation. The discovery dataset, hsa_skin1 (GSE121212), was derived from human skin biopsies where the TruSeq Stranded Total RNA Protocol was used in combination with the RiboZero rRNA removal Kit. It consisted of 28 PP, 38 NN, and 27 PN samples. One of the validation datasets, hsa_skin2 (GSE74697), was generated from human skin samples with the ScriptSeq complete kit from Epicenter and contained 18 PP and 16 NN samples. The other two datasets, hsa_MSC1 (GSE81106) and hsa_MSC2 (GSE89725), were from skin-derived Mesenchymal stem cells (MSC) and derived with the TruSeq Stranded Total RNA Library Prep kit using the CircRNA Enrichment kit and ribo-zero rRNA Removal kit, respectively. Each of the MSC datasets was from three PP and three NN samples.

### 4.2. Psoriatic Skin Biopsy Samples and Cells for Validation

Psoriatic samples (PP and PN) from skin biopsies of 30 PS patients and HaCaT keratinocyte cells were used for validation. These psoriatic samples were from patients with untreated psoriasis of moderate-to-severe intensity (PASI > 12, BSA at least 10%) and they were all collected with ethical approval obtained from The Rockefeller University Institutional Review Board (RU IRB, New York city, NY, USA). The RNA was extracted with the miRNeasy kit for purification of total RNA (Qiagen, Germantown, MD, USA).

### 4.3. RNase R Digestion

RNA was digested with RNase R (BioVision, Milpitas, CA, USA) (a 5′ to 3′ exonuclease) to degrade linear RNA and enrich for circular RNA. RNase R digested RNA was prepared with a reaction containing 2 μg of prepared RNA, 1 μL (40U) RiboLock (ThermoFisher-Scientific, Waltham, MA, USA), 2 μL 10× RNase R reaction buffer, and 1 μL (10U) RNase R; adjusting the volume to 20 μL with nuclease-free water [71]. RNA was then converted to cDNA with a Quantitect Reverse Transcription kit (Qiagen, Germantown, MD, USA).

### 4.4. PCR and Sanger Sequencing

Divergent primers (FisherScientific, Waltham, MA, USA) were designed with the CircBase IDs of circRNAs in the CircInteractome webtool, if available; otherwise, primers were manually designed across the back-fusion (BF) points. The primers used for validation are listed in Table S4. Divergent primers are outward-facing primers that should only amplify the circular RNA. Convergent primers were also designed as a control. Convergent primers can generate products from genomic DNA, whereas divergent primers cannot. The correctness of the BF sites of these amplified products was verified with Sanger sequencing (Psomagen, Brooklyn, NY, USA).

### 4.5. Quantitative Real-Time PCR

Divergent primers were used to amplify cDNA in a qRT-PCR reaction with SYBR Green (Kapa Biosystems, Wilmington, MA, USA). GAPDH was used as a reference. Both target and reference were amplified in triplicate and the relative level of each circRNA was calculated with the 2−∆∆Ct method.

### 4.6. Identification of circRNAs of All Types—The CAT Method

A new method was developed to comprehensively search for circular RNAs of all types (i.e., the CAT method) using paired-end RNA-seq data without information of splicing signals. CAT first maps sequencing reads to the reference genome with Bowtie2 [72]. The mappable reads are discarded since they are from linear RNAs. CAT takes a left and a right *x*-mer on the 5′-end and 3′-end of an unmapped read, called the left and right anchors, respectively (Figure 1a). It attempts to split-map the read to the genome—the left anchor maps to a locus that is downstream from the locus to which the right anchor maps (Figure 1a). If successful, it then searches for the split point within the sequence between the two anchors such that when the read is split at the split point, the two segments of the read can be aligned to the genomic loci that the anchors determine (Figure 1a). In the current CAT implementation for sequencing read length of 100 bp, *x* was initially set to 20, and no more than two mismatches were allowed in the mapping. Once a candidate BF point was found from one read of a pair, an additional criterion was adopted to ensure the other read was completely mapped to the region within the two BF points.

CAT retains those BF points that are supported by at least k (e.g., *k* > 2 in most of our analyses) split-mapped reads in an RNA-seq library. Furthermore, only unambiguous candidate BF points are considered. Specifically, while there certainly exist circRNAs spanning more than one exon, it is difficult to infer such circRNAs with certainty based on RNA-seq data alone, particularly on RNA libraries not enriched for circRNAs. Therefore, circRNAs from single introns, single exons, pairs of adjacent exons and introns, and intergenic non-coding transcripts are considered if RNA-seq libraries are not RNase R treated (Table S2). The CAT software in python is available in the github repository at https://github.com/xiaoxin8712/find_circ_strand (accessed on 27 July 2019).

### 4.7. Identification of Differentially Expressed circRNAs

Two criteria were introduced to identify abundantly expressed circRNAs. First, the minimal number of reads mapped to a BF point in at least one sample is no less than *k* = 5. Second, the total number of samples in which the circRNA appears is no less than *m*, and a parameter is adjusted for different sample sizes; in our experiment, *m* = 10. The circRNAs that did not meet these two criteria were considered as having low expression. The expression levels of highly expressed circRNAs were normalized by SRPBM (spliced reads per billion mappings), i.e., the number of circular reads/number of mapped reads (units in billion), to ameliorate the effects of sequencing depth and batch effects. Differentially expressed (DE) circRNAs were identified by the RankSum method [73] with a threshold of *p*-value ≤ 0.01 based on SPRBM.

### 4.8. Identification of Differentially Expressed Genes

DE genes of PP versus NN and PN were obtained with the Tophat and Cufflinks pipelines [74], with the threshold being adjusted to *q* < 0.05 and fold change >2.

### 4.9. GO and KEGG Pathway Analyses

Analyses of GO and KEGG pathway enrichment were carried out with Metascape [75] with the default parameters: minimum overlap of 3, *p*-value cutoff of 0.01, and minimum enrichment of 1.5, with GO biological processes and KEGG pathway for enrichment generated separately.

### 4.10. CircRNA-Associated Genes

CircRNA-associated mRNAs were defined as DE mRNAs that were positively regulated by circRNAs via circRNA-miRNA-mRNA regulatory cascades. That is, a DE mRNA was associated with a circRNA if both were bound and regulated by a common miRNA. mRNA and circRNA targets of mature miRNAs [76] were predicted with miRanda [77] using the default parameters of a score of 140.0 and energy of −1.0. CircRNA-associated genes were selected from the DE genes that were positively correlated with their corresponding circRNAs, i.e., Spearman’s rank correlation coefficient > 0.0 and *p*-value < 0.01.

## 5. Conclusions

Overall, our results reveal and demonstrate the diversity of circRNAs and the functional complexity of circRNAs operating in psoriasis. There exist not only circRNAs originating from exon-intron boundaries, but also i-circRNAs from interior regions of exons, introns, and intergenic regions. Importantly, circRNAs may function as ce-RNAs and contribute to psoriasis pathogenesis. Our results suggest that CDR1as, hsa_skin_088763, and hsa_skin_052271 should be analyzed in future studies as novel biomarkers for PS diagnosis.

## Data Availability

The discovery dataset hsa_skin1 used in this study is available in GEO at https://www.ncbi.nlm.nih.gov/geo/query/acc.cgi?acc=GSE121212 (accessed on 6 February 2019), reference number GSE121212 [78]. The validation datasets hsa_skin2, hsa_MSC1 and hsa_MSC2 used in this study are available at GEO https://www.ncbi.nlm.nih.gov/geo/query/acc.cgi?acc=GSE74697 (accessed on 20 December 2017), https://www.ncbi.nlm.nih.gov/geo/query/acc.cgi?acc=GSE81106 (accessed on 28 November 2018), and https://www.ncbi.nlm.nih.gov/geo/query/acc.cgi?acc=GSE89725 (accessed on 29 November 2017), reference number GSE74697 [79,80], GSE81106 [44], and GSE89725, respectively.

## Supplementary Materials

The following are available online at https://www.mdpi.com/article/10.3390/ijms22105182/s1, Figure S1: dataset and distribution of circRNAs, Figure S2: experimental validation of three identified circRNAs, CDR1as, hsa_skin_088763 and hsa_skin_052271 in psoriatic skin and HaCaT keratinocyte cells, Figure S3: pathways enriched by circRNA-associated genes between PP and NN, Figure S4: pathways enriched by circRNA-associated genes between PP and PN, Figure S5: pathways enriched by circRNA-associated genes between PN and NN, Figure S6: pathways enriched by differentially expressed genes between PP and NN, Table S1: RNA-seq datasets, Table S2: 179 abundant circRNAs (expressed in at least 10 samples and supported by at least five reads in at least one sample, GRCh38), Table S3: genes significantly differentially expressed between PP and NN, Table S4: divergent and convergent primers for the validation experiments, Table S5: associated genes of three validated circRNAs, CDR1as, hsa_skin_088763 and hsa_skin_052271, Table S6: CircRNAs significantly differentially expressed between PP and NN (*p*-value < 0.01).

## References

[1] Harden J.L., Krueger J.G., Bowcock A.M. (2015). The immunogenetics of psoriasis: A comprehensive review. J. Autoimmun..

[2] World Health Organization (2016). Global Report on Psoriasis.

[3] Gelfand J.M., Dommasch E.D., Shin D.B., Azfar R.S., Kurd S.K., Wang X., Troxel A.B. (2009). The risk of stroke in patients with psoriasis. J. Investig. Dermatol..

[4] Gelfand J.M., Neimann A.L., Shin D.B., Wang X., Margolis D.J., Troxel A.B. (2006). Risk of myocardial infarction in patients with psoriasis. JAMA.

[5] Li W., Han J., Hu F.B., Curhan G.C., Qureshi A.A. (2012). Psoriasis and risk of type 2 diabetes among women and men in the United States: A population-based cohort study. J. Investig. Dermatol..

[6] Sommer D.M., Jenisch S., Suchan M., Christophers E., Weichenthal M. (2007). Increased prevalence of the metabolic syndrome in patients with moderate to severe psoriasis. Arch. Dermatol. Res..

[7] Gisondi P., Fostini A.C., Fossà I., Girolomoni G., Targher G. (2018). Psoriasis and the metabolic syndrome. Clin. Dermatol..

[8] Fuxench Z.C.C., Shin D.B., Beatty A.O., Gelfand J.M. (2016). The risk of cancer in patients with psoriasis: A population-based cohort study in the health improvement network. JAMA Dermatol..

[9] Trafford A.M., Parisi R., Kontopantelis E., Griffiths C.E., Ashcroft D.M. (2019). Association of Psoriasis With the Risk of Developing or Dying of Cancer: A Systematic Review and Meta-analysis. JAMA Dermatol..

[10] Strange A., Capon F., Spencer C.C., Knight J., Weale M.E., Allen M.H., Barton A., Band G., Bellenguez C., Bergboer J.G. (2010). A genome-wide association study identifies new psoriasis susceptibility loci and an interaction between HLA-C and ERAP1. Nat. Genet..

[11] Zhang X.-J., Huang W., Yang S., Sun L.-D., Zhang F.-Y., Zhu Q.-X., Zhang F.-R., Zhang C., Du W.-H., Pu X.-M. (2009). Psoriasis genome-wide association study identifies susceptibility variants within LCE gene cluster at 1q21. Nat. Genet..

[12] Xia J., Zhang W. (2013). MicroRNAs in normal and psoriatic skin. Physiol. Genom..

[13] Sonkoly E., Wei T., Janson P.C., Sääf A., Lundeberg L., Tengvall-Linder M., Norstedt G., Alenius H., Homey B., Scheynius A. (2007). MicroRNAs: Novel regulators involved in the pathogenesis of psoriasis?. PLoS ONE.

[14] Xia J., Joyce C.E., Bowcock A.M., Zhang W. (2012). Noncanonical microRNAs and endogenous siRNAs in normal and psoriatic human skin. Hum. Mol. Genet..

[15] Joyce C.E., Zhou X., Xia J., Ryan C., Thrash B., Menter A., Zhang W., Bowcock A.M. (2011). Deep sequencing of small RNAs from human skin reveals major alterations in the psoriasis miRNAome. Hum. Mol. Genet..

[16] Tsoi L.C., Iyer M.K., Stuart P.E., Swindell W.R., Gudjonsson J.E., Tejasvi T., Sarkar M.K., Li B., Ding J., Voorhees J.J. (2015). Analysis of long non-coding RNAs highlights tissue-specific expression patterns and epigenetic profiles in normal and psoriatic skin. Genome Biol..

[17] Meisgen F., Xu N., Wei T., Janson P.C., Obad S., Broom O., Nagy N., Kauppinen S., Kemény L., Ståhle M. (2012). MiR-21 is up-regulated in psoriasis and suppresses T cell apoptosis. Exp. Dermatol..

[18] Xu N., Meisgen F., Butler L.M., Han G., Wang X.-J., Söderberg-Nauclér C., Ståhle M., Pivarcsi A., Sonkoly E. (2013). MicroRNA-31 is overexpressed in psoriasis and modulates inflammatory cytokine and chemokine production in keratinocytes via targeting serine/threonine kinase 40. J. Immunol..

[19] Sonkoly E., Bata-Csorgo Z., Pivarcsi A., Polyanka H., Kenderessy-Szabo A., Molnar G., Szentpali K., Bari L., Megyeri K., Mandi Y. (2005). Identification and characterization of a novel, psoriasis susceptibility-related noncoding RNA gene, PRINS. J. Biol. Chem..

[20] Széll M., Danis J., Bata-Csörgő Z., Kemény L. (2016). PRINS, a primate-specific long non-coding RNA, plays a role in the keratinocyte stress response and psoriasis pathogenesis. Pflügers Arch. Eur. J. Physiol..

[21] Cui H., Xie N., Tan Z., Banerjee S., Thannickal V.J., Abraham E., Liu G. (2014). The human long noncoding RNA lnc-IL 7 R regulates the inflammatory response. Eur. J. Immunol..

[22] Hu G., Tang Q., Sharma S., Yu F., Escobar T.M., Muljo S.A., Zhu J., Zhao K. (2013). Expression and regulation of intergenic long noncoding RNAs during T cell development and differentiation. Nat. Immunol..

[23] Memczak S., Jens M., Elefsinioti A., Torti F., Krueger J., Rybak A., Maier L., Mackowiak S.D., Gregersen L.H., Munschauer M. (2013). Circular RNAs are a large class of animal RNAs with regulatory potency. Nature.

[24] Salzman J., Gawad C., Wang P.L., Lacayo N., Brown P.O. (2012). Circular RNAs are the predominant transcript isoform from hundreds of human genes in diverse cell types. PLoS ONE.

[25] Lu C., Sun X., Li N., Wang W., Kuang D., Tong P., Han Y., Dai J. (2018). CircRNAs in the tree shrew (Tupaia belangeri) brain during postnatal development and aging. Aging.

[26] Wang P.L., Bao Y., Yee M.-C., Barrett S.P., Hogan G.J., Olsen M.N., Dinneny J.R., Brown P.O., Salzman J. (2014). Circular RNA is expressed across the eukaryotic tree of life. PLoS ONE.

[27] Li X., Yang L., Chen L.-L. (2018). The biogenesis, functions, and challenges of circular RNAs. Mol. Cell.

[28] Hansen T.B., Jensen T.I., Clausen B.H., Bramsen J.B., Finsen B., Damgaard C.K., Kjems J. (2013). Natural RNA circles function as efficient microRNA sponges. Nature.

[29] Li Z., Huang C., Bao C., Chen L., Lin M., Wang X., Zhong G., Yu B., Hu W., Dai L. (2015). Exon-intron circular RNAs regulate transcription in the nucleus. Nat. Struct. Mol. Biol..

[30] Hansen T.B., Wiklund E.D., Bramsen J.B., Villadsen S.B., Statham A.L., Clark S.J., Kjems J. (2011). miRNA-dependent gene silencing involving Ago2-mediated cleavage of a circular antisense RNA. EMBO J..

[31] Zhong Y., Du Y., Yang X., Mo Y., Fan C., Xiong F., Ren D., Ye X., Li C., Wang Y. (2018). Circular RNAs function as ceRNAs to regulate and control human cancer progression. Mol. Cancer.

[32] Ashwal-Fluss R., Meyer M., Pamudurti N.R., Ivanov A., Bartok O., Hanan M., Evantal N., Memczak S., Rajewsky N., Kadener S. (2014). circRNA biogenesis competes with pre-mRNA splicing. Mol. Cell.

[33] Pamudurti N.R., Bartok O., Jens M., Ashwal-Fluss R., Stottmeister C., Ruhe L., Hanan M., Wyler E., Perez-Hernandez D., Ramberger E. (2017). Translation of circRNAs. Mol. Cell.

[34] Yang Y., Fan X., Mao M., Song X., Wu P., Zhang Y., Jin Y., Yang Y., Chen L.-L., Wang Y. (2017). Extensive translation of circular RNAs driven by N 6-methyladenosine. Cell Res..

[35] Legnini I., Di Timoteo G., Rossi F., Morlando M., Briganti F., Sthandier O., Fatica A., Santini T., Andronache A., Wade M. (2017). Circ-ZNF609 is a circular RNA that can be translated and functions in myogenesis. Mol. Cell.

[36] Lukiw W. (2013). Circular RNA (circRNA) in Alzheimer’s disease (AD). Front. Genet..

[37] Zhao Y., Alexandrov P., Jaber V., Lukiw W. (2016). Deficiency in the ubiquitin conjugating enzyme UBE2A in Alzheimer’s disease (AD) is linked to deficits in a natural circular miRNA-7 sponge (circRNA; ciRS-7). Genes.

[38] Zhou Z.-B., Huang G.-X., Fu Q., Han B., Lu J.-J., Chen A.-M., Zhu L. (2019). circRNA. 33186 Contributes to the Pathogenesis of Osteoarthritis by Sponging miR-127-5p. Mol. Ther..

[39] Altesha M.A., Ni T., Khan A., Liu K., Zheng X. (2019). Circular RNA in cardiovascular disease. J. Cell. Physiol..

[40] Fan X., Weng X., Zhao Y., Chen W., Gan T., Xu D. (2017). Circular RNAs in cardiovascular disease: An overview. Biomed Res. Int..

[41] Jiang L.-H., Sun D.-W., Hou J.-C., Ji Z.-L. (2018). CircRNA: A novel type of biomarker for cancer. Breast Cancer.

[42] Feng J., Chen K., Dong X., Xu X., Jin Y., Zhang X., Chen W., Han Y., Shao L., Gao Y. (2019). Genome-wide identification of cancer-specific alternative splicing in circRNA. Mol. Cancer.

[43] Qiao M., Ding J., Yan J., Li R., Jiao J., Sun Q. (2018). Circular RNA expression profile and analysis of their potential function in psoriasis. Cell. Physiol. Biochem..

[44] Liu R., Chang W., Li J., Cheng Y., Dang E., Yang X., Wang Q., Wang G., Li X., Zhang K. (2019). Mesenchymal stem cells in psoriatic lesions affect the skin microenvironment through circular RNA. Exp. Dermatol..

[45] Moldovan L.-I., Hansen T.B., Venø M.T., Okholm T.L.H., Andersen T.L., Hager H., Iversen L., Kjems J., Johansen C., Kristensen L.S. (2019). High-throughput RNA sequencing from paired lesional-and non-lesional skin reveals major alterations in the psoriasis circRNAome. BMC Med. Genom..

[46] Liu X., Hu Z., Zhou J., Tian C., Tian G., He M., Gao L., Chen L., Li T., Peng H. (2020). Interior circular RNA. RNA Biol..

[47] Gao Y., Zhang J., Zhao F. (2018). Circular RNA identification based on multiple seed matching. Brief. Bioinform..

[48] Zhang X.-O., Dong R., Zhang Y., Zhang J.-L., Luo Z., Zhang J., Chen L.-L., Yang L. (2016). Diverse alternative back-splicing and alternative splicing landscape of circular RNAs. Genome Res..

[49] Cheng J., Metge F., Dieterich C. (2016). Specific identification and quantification of circular RNAs from sequencing data. Bioinformatics.

[50] Xiao M.-S., Wilusz J.E. (2019). An improved method for circular RNA purification using RNase R that efficiently removes linear RNAs containing G-quadruplexes or structured 3’ ends. Nucleic Acids Res..

[51] Hansen T.B., Venø M.T., Damgaard C.K., Kjems J. (2016). Comparison of circular RNA prediction tools. Nucleic Acids Res..

[52] Salmena L., Poliseno L., Tay Y., Kats L., Pandolfi P.P. (2011). A ceRNA hypothesis: The Rosetta Stone of a hidden RNA language?. Cell.

[53] Choi H.-R., Nam K.-M., Park S.-J., Kim D.-S., Huh C.-H., Park W.-Y., Park K.-C. (2014). Suppression of miR135b increases the proliferative potential of normal human keratinocytes. J. Investig. Dermatol..

[54] Kim K.-H., Son E.D., Kim H.-J., Lee S.H., Bae I.-H., Lee T.R. (2019). EGR3 Is a Late Epidermal Differentiation Regulator that Establishes the Skin-Specific Gene Network. J. Investig. Dermatol..

[55] Campanati A., Consales V., Orciani M., Giuliodori K., Ganzetti G., Bobyr I., Sorgentoni G., di Primio R., Offidani A. (2017). Role of mesenchymal stem cells in the pathogenesis of psoriasis: Current perspectives. Psoriasis.

[56] Rácz E., Kurek D., Kant M., Baerveldt E.M., Florencia E., Mourits S., de Ridder D., Laman J.D., van der Fits L., Prens E.P. (2011). GATA3 expression is decreased in psoriasis and during epidermal regeneration; induction by narrow-band UVB and IL-4. PLoS ONE.

[57] Løvendorf M.B., Zibert J.R., Gyldenløve M., Røpke M.A., Skov L. (2014). MicroRNA-223 and miR-143 are important systemic biomarkers for disease activity in psoriasis. J. Dermatol. Sci..

[58] Su Y., Huang J., Zhao X., Lu H., Wang W., Yang X.O., Shi Y., Wang X., Lai Y., Dong C. (2019). Interleukin-17 receptor D constitutes an alternative receptor for interleukin-17A important in psoriasis-like skin inflammation. Sci. Immunol..

[59] Sundarrajan S., Lulu S., Arumugam M. (2015). Insights into protein interaction networks reveal non-receptor kinases as significant druggable targets for psoriasis. Gene.

[60] Karlsson T., Mark E.B., Henriksson R., Hedman H. (2008). Redistribution of LRIG proteins in psoriasis. J. Investig. Dermatol..

[61] Sundberg T.B., Liang Y., Wu H., Choi H.G., Kim N.D., Sim T., Johannessen L., Petrone A., Khor B., Graham D.B. (2016). Development of chemical probes for investigation of salt-inducible kinase function in vivo. ACS Chem. Biol..

[62] Gilhar A., Yaniv R., Assy B., Serafimovich S., Ullmann Y., Kalish R.S. (2006). Fas pulls the trigger on psoriasis. Am. J. Pathol..

[63] Hansen T.B., Kjems J., Damgaard C.K. (2013). Circular RNA and miR-7 in cancer. Cancer Res..

[64] Li P., Yang X., Yuan W., Yang C., Zhang X., Han J., Wang J., Deng X., Yang H., Li P. (2018). CircRNA-Cdr1as exerts anti-oncogenic functions in bladder Cancer by sponging MicroRNA-135a. Cell. Physiol. Biochem..

[65] Xu H., Guo S., Li W., Yu P. (2015). The circular RNA Cdr1as, via miR-7 and its targets, regulates insulin transcription and secretion in islet cells. Sci. Rep..

[66] Albanesi C., Madonna S., Gisondi P., Girolomoni G. (2018). The interplay between keratinocytes and immune cells in the pathogenesis of psoriasis. Front. Immunol..

[67] Chen Z., Gao Y., Gao S., Song D., Feng Y. (2020). MiR-135b-5p promotes viability, proliferation, migration and invasion of gastric cancer cells by targeting Krüppel-like factor 4 (KLF4). Arch. Med Sci. AMS.

[68] Segre J.A., Bauer C., Fuchs E. (1999). Klf4 is a transcription factor required for establishing the barrier function of the skin. Nat. Genet..

[69] Ye C.Y., Chen L., Liu C., Zhu Q.H., Fan L. (2015). Widespread noncoding circular RNA s in plants. New Phytol..

[70] Guarnerio J., Bezzi M., Jeong J.C., Paffenholz S.V., Berry K., Naldini M.M., Lo-Coco F., Tay Y., Beck A.H., Pandolfi P.P. (2016). Oncogenic role of fusion-circRNAs derived from cancer-associated chromosomal translocations. Cell.

[71] Panda A.C., Gorospe M. (2018). Detection and analysis of circular RNAs by RT-PCR. Bio-Protocol.

[72] Langmead B., Trapnell C., Pop M., Salzberg S.L. (2009). Ultrafast and memory-efficient alignment of short DNA sequences to the human genome. Genome Biol..

[73] Laing E., Smith C.P. (2010). RankProdIt: A web-interactive Rank Products analysis tool. BMC Res. Notes.

[74] Trapnell C., Roberts A., Goff L., Pertea G., Kim D., Kelley D.R., Pimentel H., Salzberg S.L., Rinn J.L., Pachter L. (2012). Differential gene and transcript expression analysis of RNA-seq experiments with TopHat and Cufflinks. Nat. Protoc..

[75] Zhou Y., Zhou B., Pache L., Chang M., Khodabakhshi A.H., Tanaseichuk O., Benner C., Chanda S.K. (2019). Metascape provides a biologist-oriented resource for the analysis of systems-level datasets. Nat. Commun..

[76] Griffiths-Jones S., Grocock R.J., Van Dongen S., Bateman A., Enright A.J. (2006). miRBase: microRNA sequences, targets and gene nomenclature. Nucleic Acids Res..

[77] Enright A.J., John B., Gaul U., Tuschl T., Sander C., Marks D.S. (2003). MicroRNA targets in Drosophila. Genome Biol..

[78] Tsoi L.C., Rodriguez E., Degenhardt F., Baurecht H., Wehkamp U., Volks N., Szymczak S., Swindell W.R., Sarkar M.K., Raja K. (2019). Atopic Dermatitis Is an IL-13–Dominant Disease with Greater Molecular Heterogeneity Compared to Psoriasis. J. Investig. Dermatol..

[79] Ahn R., Gupta R., Lai K., Chopra N., Arron S.T., Liao W. (2016). Network analysis of psoriasis reveals biological pathways and roles for coding and long non-coding RNAs. BMC Genom..

[80] Gupta R., Ahn R., Lai K., Mullins E., Debbaneh M., Dimon M., Arron S., Liao W. (2016). Landscape of long noncoding RNAs in psoriatic and healthy skin. J. Investig. Dermatol..

