# Supplementary material for: Canonical and Interior Circular RNAs Function as Competing Endogenous RNAs in Psoriatic Skin
Source: Int J Mol Sci. 2021 May 13;22(10):5182. doi: 10.3390/ijms22105182 (PMC8153647; doi:10.3390/ijms22105182)
Supplement: Supplementary file 1 [file ijms-22-05182-s001.zip › ijms-1223516-supplementary.pdf]

Supplementary Online Content

Figure S1. Dataset and distribution of circRNAs.
Figure S2. Experimental validation of three identified circRNAs, *CDR1as*, *hsa_skin_088763* and *hsa_skin_052271* in psoriatic skin and HaCaT keratinocyte cells.
Figure S3. Pathways enriched by circRNA-associated genes between PP and NN.
Figure S4. Pathways enriched by circRNA-associated genes between PP and PN.
Figure S5. Pathways enriched by circRNA-associated genes between PN and NN.
Figure S6. Pathways enriched by differentially expressed genes between PP and NN.
Table S1. RNA-seq datasets
Table S2. 179 abundant circRNAs (expressed in at least 10 samples and supported by at least 5 reads in at least one sample, GRCh38)
Table S3. Genes significantly differentially expressed between PP and NN
Table S4. Divergent and convergent primers for the validation experiments
Table S5. Associated genes of three validated circRNAs, *CDR1as*, *hsa_skin_088763* and *hsa_skin_052271*
Table S6. CircRNAs significantly differentially expressed between PP and NN (p-value < 0.01)

figures

(a)

Datasets	Name	Tissue	PP	PN	NN	Total
Discovery	hsa_skin1	Skin	28	27	38	93
Validation	hsa_skin2	Skin	18	0	16	34
	hsa_MSC1	MSC	3	0	3	6
	hsa_MSC2	MSC	3	0	3	6
Total			52	27	60	139

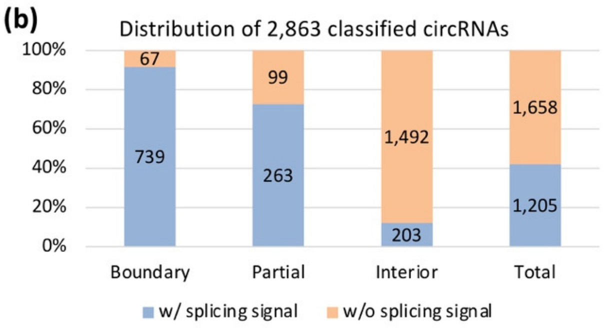

Figure S1. Dataset and distribution of circRNAs. **(a)** Distribution among psoriatic-involved (PP), psoriatic-uninvolved (PN) and normal (NN) skin of the 139 samples from four datasets: *hsa_MSC1*, *hsa_MSC2*, *hsa_skin1* and *hsa_skin2*. **(b)** Distribution of 2,863 annotated circRNAs and i-circRNAs in four datasets that are supported by at least 2 reads. Boundary circRNAs are canonical circRNAs, and i-circRNAs are further classified into *partial and complete i-circRNAs*. All circRNAs are further annotated as whether being adjacent to splicing signals or not.

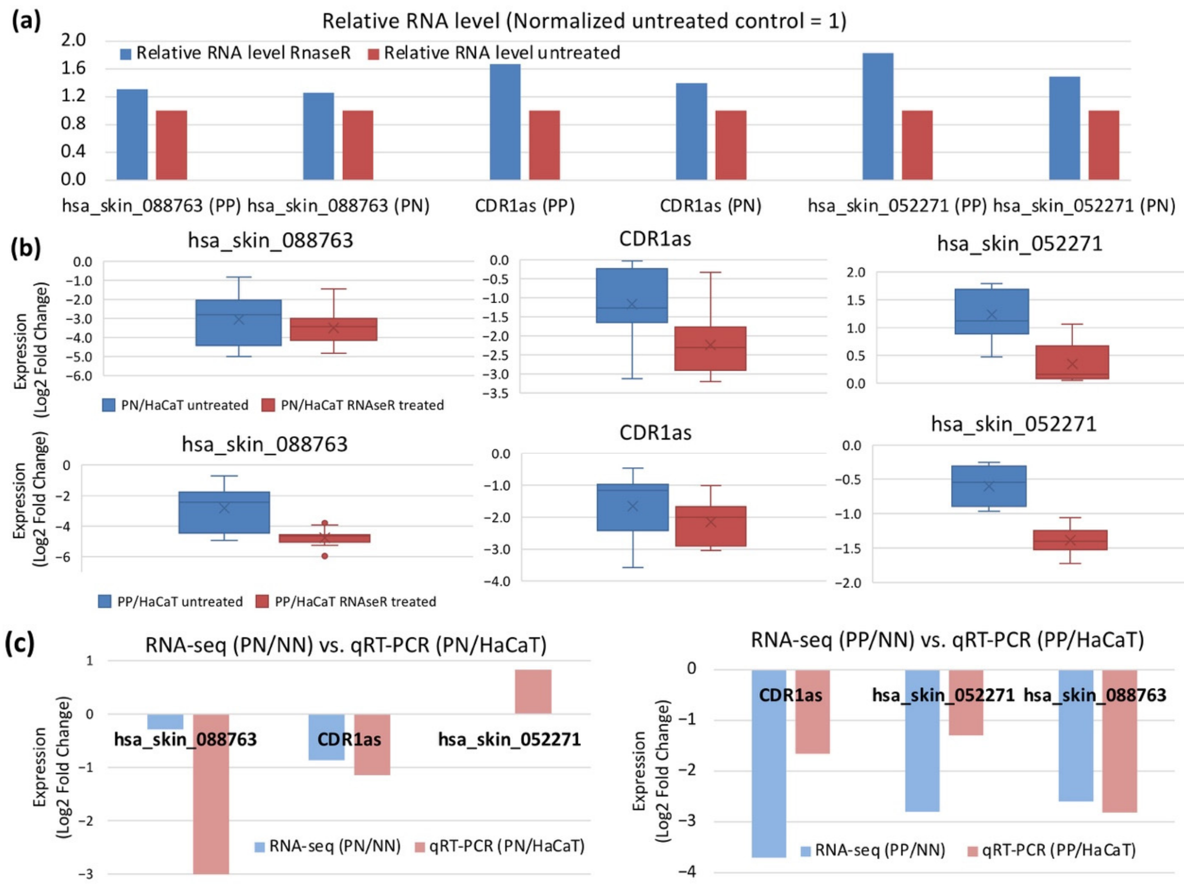

Figure S2. Experimental validation of three identified circRNAs, *CDR1as*, *hsa_skin_088763* and *hsa_skin_052271* in psoriatic skin and HaCaT keratinocyte cells. **(a)** Validation of existence of circRNAs in PP and PN skin by RNase R treatment. **(b)** Box and whisker plot for RNaseR and **(c)** Quantitative real-time (qRT-PCR) validation of differentially expressed circRNAs *hsa_skin_088763*, *CDR1as*, and *hsa_skin_052271* between PN vs. HaCaT and PP vs. HaCaT.

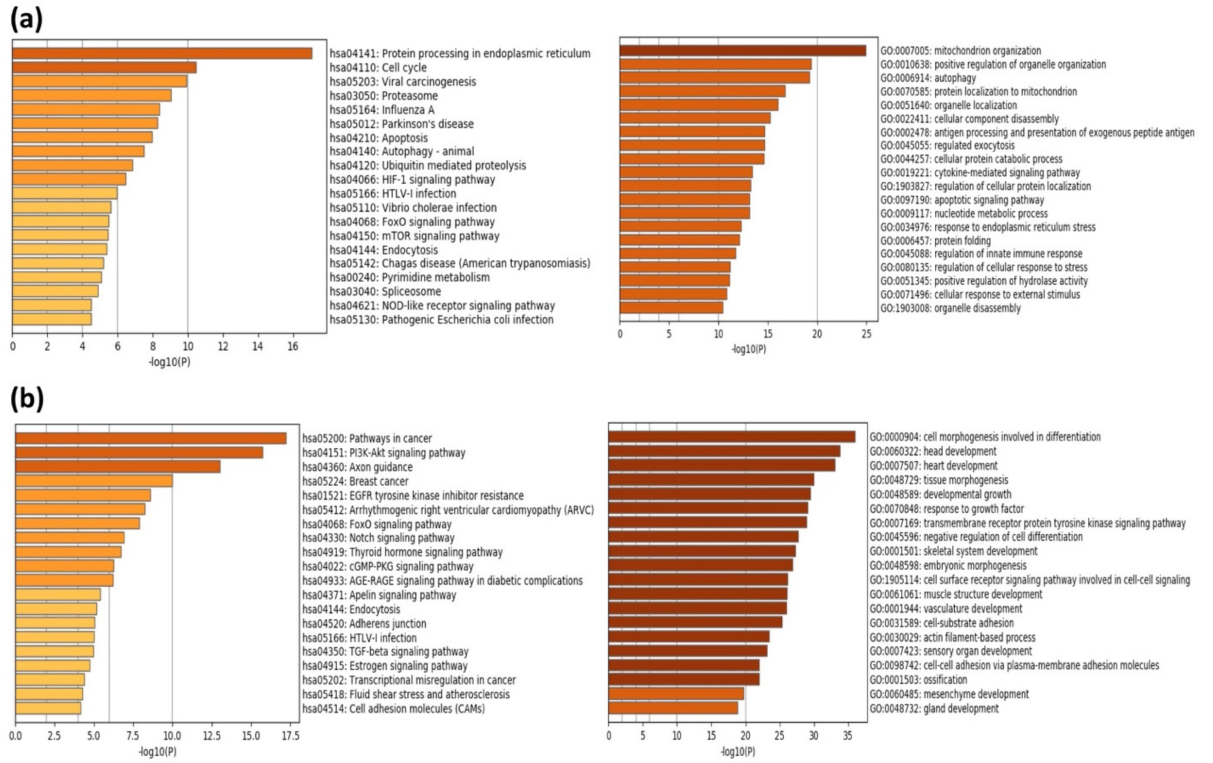

Figure S3. Pathways enriched by circRNA-associated genes between PP and NN. Enriched KEGG pathways (left) and GO pathways (right) by **(a)** up-regulated and **(b)** down-regulated circRNA-associated genes in PP vs. NN.

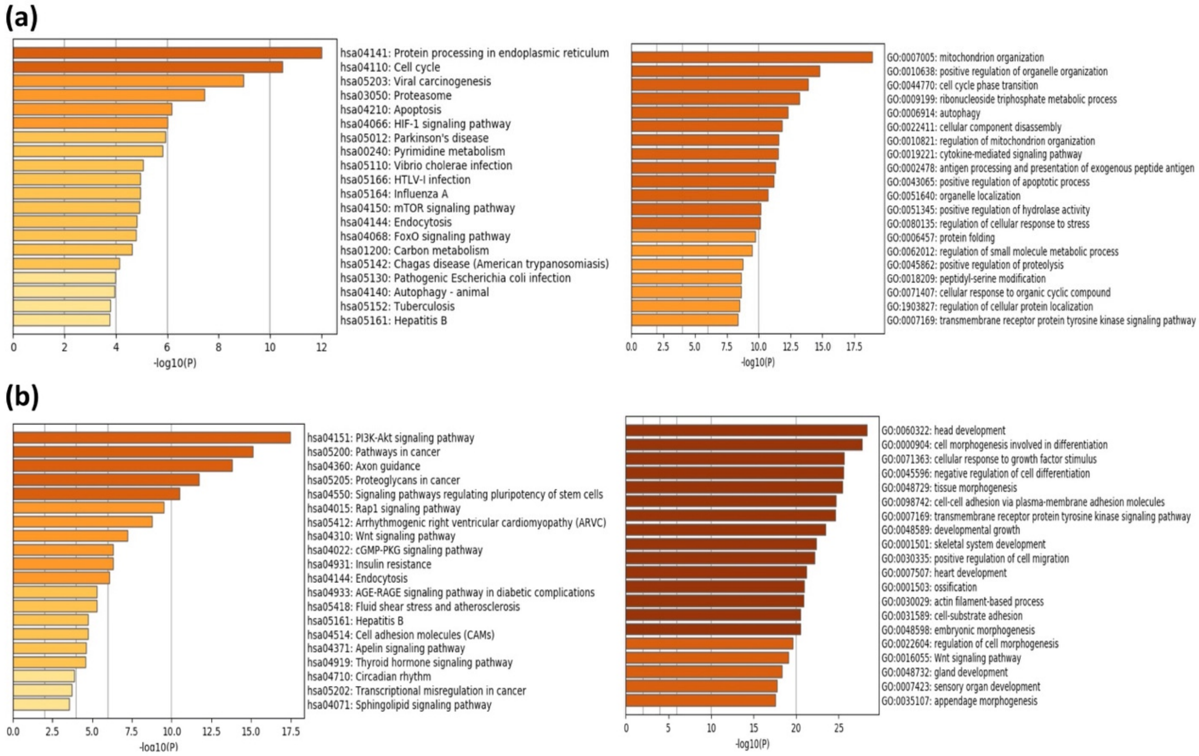

Figure S4. Pathways enriched by circRNA-associated genes between PP and PN. Enriched KEGG pathways (left) and GO pathways (right) by **(a)** up-regulated and **(b)** down-regulated circRNA-associated genes in PP vs. PN.

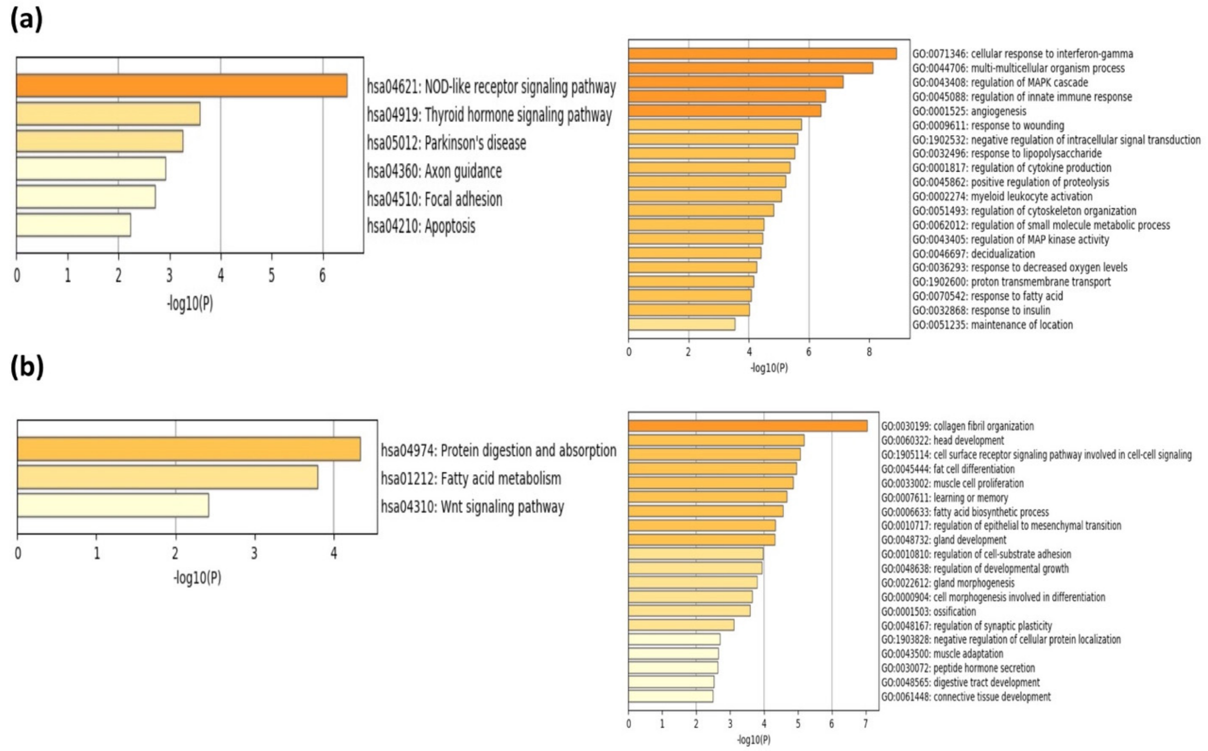

Figure S5. Pathways enriched by circRNA-associated genes between PN and NN. Enriched KEGG pathways (left) and GO pathways (right) by **(a)** up-regulated and **(b)** down-regulated circRNA-associated genes in PN vs. NN.

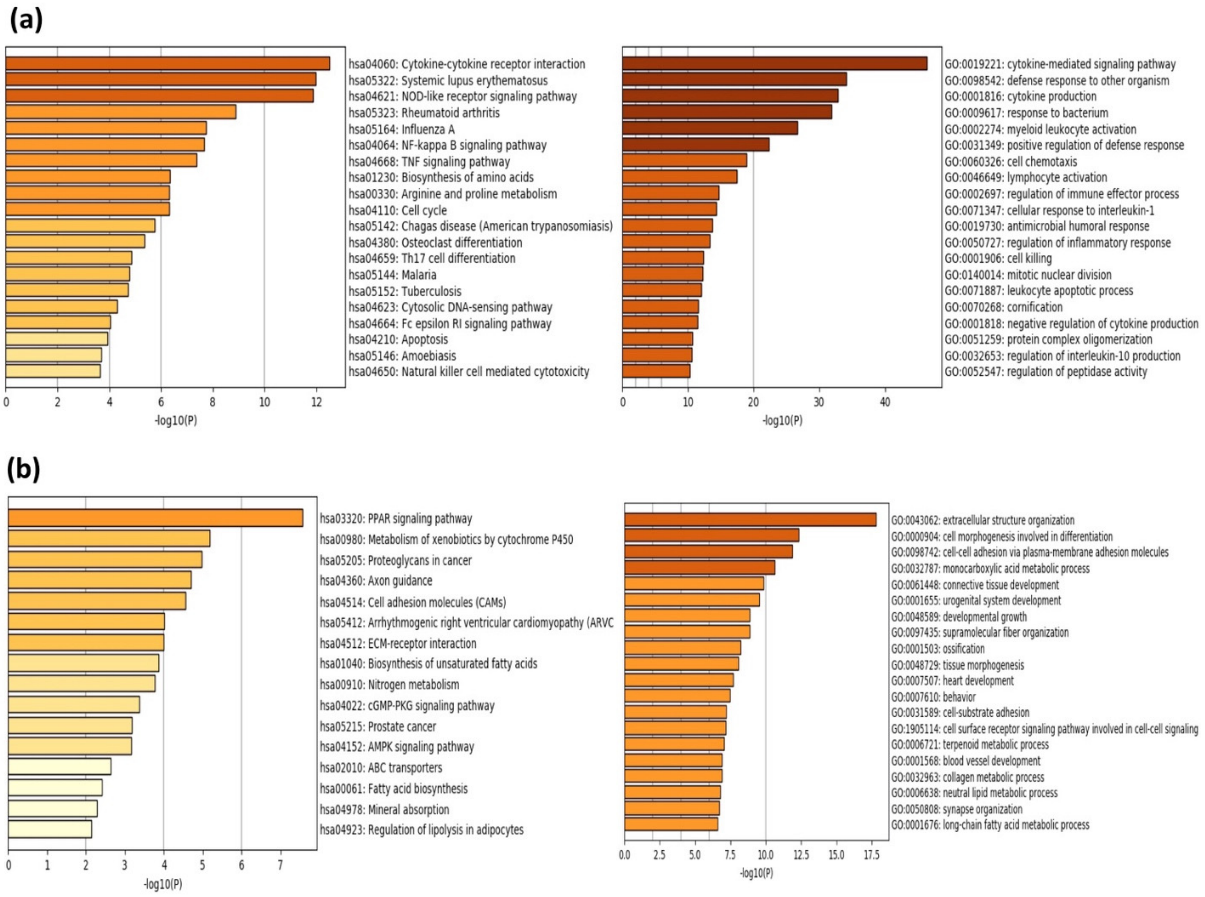

Figure S6. Pathways enriched by differentially expressed genes between PP and NN. Enriched KEGG pathways (left) and GO pathways (right) by **(a)** up-regulated and **(b)** down-regulated genes.

Table S1. RNA-seq datasets

Name	Organism	Tissue	RNA library preparation	# samples	Accession
hsa_MSC1	homo sapiens	Skin-derived Mesenchymal stem cells	circRNA enriched, stranded total RNA, paired-end	6	GSE81106
hsa_MSC2	homo sapiens	Skin-derived Mesenchymal stem cells	ribo-zero, stranded total RNA,paired-end	6	GSE89725
hsa_skin1	homo sapiens	skin	ribo-zero, stranded total RNA, paired-end	93	GSE121212
hsa_skin2	homo sapiens	skin	ribosome-depleted, stranded total RNA, paired-end	34	GSE74697

Table S2. 179 Abundant circRNAs (expressed in at least 10 samples && supported by at least 5 reads in at least one sample, GRCh38)

chrom: chromosome of circRNA
start: start position or possible region (zero based) of circRNA
end: end position or possible region (exculsive) of circRNA
s: strand orientation
name: circRNA identifier
reads: number of supporting reads
uniq: number of unique supporting reads
ss: splicing signal, 2 = major signal AG/GT, 1 = minor signal AC/AT, 0 = no signal
len: distance between start and end postion
SHS: short homologous sequence
host_gene: genes overlapping with circRNAs
anno: type of annotations (exon/intron) overlap with circRNA
type: Whether the candidate originated from annotation boundary. B:boundary, I: Interior, 5': 5' boundary, 3': 3' boundary.
cs: Complementary sequences: Whether the 100-bp sequence outside of circRNA is complementary or not

chrom	start	end	s	name	reads	uniq	ss	len	SHS	host_gene	anno	type	cs
chrX	140783169-140783175	140784654-140784660	+	hsa_skin_194345 (CDR1as)	1440	262	2	1485	TCCAGG	CDR1-AS	intergenic	I	no
chr1	152352401-152352423	152352626-152352648	-	hsa_skin_052271	22	12	0	225	CCACACAG ACAGGGTC CAGGAC	FLG2	exon	I	no
chr19	23831593-23831599	23833518-23833524	+	hsa_skin_088763	405	108	2	1925	TAGGTA	RP11-255H23.2	exon	B	no
chr3	37325180-37325187	37325378-37325385	+	hsa_skin_124531	18	8	0	198	GAAGAAG	GOLGA4	intron; exon	I	no
chr11	65499641-65499650	65499860-65499869	+	hsa_skin_006690	48	31	0	219	AAGATAGAA	MALAT1	intron; exon	I	no
chr1	62161623-62161626	62162662-62162665	+	hsa_skin_212178	74	27	0	1039	CCA	INADL	intron; exon	I	fold
chr11	65504856-65504859	65505049-65505052	+	hsa_skin_228518	50	25	0	193	AAG	MALAT1	exon	I	no
chr17	7576810-7576812	7576950-7576952	+	hsa_skin_169953	99	18	0	140	GC	EIF4A1; RP11-186B7.4; SENP3-EIF4A1; SNORD10	exon	3' B	no
chr19	35497284-35497288	35497481-35497485	-	hsa_skin_017574	318	103	0	197	CTCC	DMKN	exon	3' B	no
chr10	124942455-124942456	124943306-124943307	+	hsa_skin_142248	529	144	2	851	G	RP11-298J20.4; ZRANB1	exon	3' B	no
chr1	152354735-152354791	152354966-152355022	-	hsa_skin_194228	65	24	1	231	TATGGCCA ACATGGTT CTGGCTCA AGTCAGTC ATCTGGCT ATGGTCAA CATGGGTC	FLG2	exon	I	fold
chr12	52645094-52645118	52645241-52645265	-	hsa_skin_151142	77	36	0	147	CTCTGGAG GAGGATAT GGCTCTGG	KRT2	exon	I	no
chr17	39254341-39254346	39255167-39255172	-	hsa_skin_017093	25	21	0	826	AGCCA	FBXL20	exon	I	fold
chr1	152314125-152314130	152314264-152314269	-	hsa_skin_226345	19	10	0	139	AGAAA	FLG	exon	I	no
chr20	25297074-25297079	25297389-25297394	+	hsa_skin_023969	26	15	0	315	CTGCC	PYGB	exon	I	no
chr12	52645124-52645131	52645271-52645278	-	hsa_skin_060631	51	30	0	147	GAGGAGG	KRT2	exon	I	no

chrom	start	end	s	name	reads	uniq	ss	len	SHS	host_gene	anno	type	cs
chr19	35497289-35497290	35497483-35497484	-	hsa_skin_070366	308	98	0	194	T	DMKN	exon	I	no
chr1	152314108-152314115	152314276-152314283	-	hsa_skin_175896	70	33	0	168	TGAAAGA	FLG	exon	I	no
chr3	188882983-188882986	188883478-188883481	+	hsa_skin_126952	143	38	0	495	TCC	LPP	exon	I	fold
chr10	86925982-86925988	86926267-86926273	+	hsa_skin_020081	45	20	0	285	TCCCAA	BMPR1A	exon	I	fold
chr1	152356347-152356353	152356497-152356503	-	hsa_skin_093700	34	14	0	150	GCTTTG	FLG2	exon	I	no
chr12	52644846-52644852	52644989-52644995	-	hsa_skin_057157	70	19	0	143	CTCTCC	KRT2	exon	I	no
chr2	54972968-54972975	54973124-54973131	-	hsa_skin_055859	31	14	0	156	GTTCATC	RTN4	exon	I	no
chr3	188880286-188880293	188880862-188880869	+	hsa_skin_038904	63	28	0	576	TCCCAAA	LPP	exon	I	fold
chr3	179398017-179398019	179398266-179398268	-	hsa_skin_084435	67	30	0	249	TG	GNB4	exon	I	fold
chr2	173954872-173954875	173956232-173956235	-	hsa_skin_009497	366	124	2	1360	CAG	SP3	exon	B	no
chr11	12764177-12764180	12764433-12764436	+	hsa_skin_186524	65	32	2	256	GGT	TEAD1	exon	B	no
chr8	38819521	38820635	+	hsa_skin_044843	23	14	2	1114	-	TACC1	exon	B	no
chr10	68644696-68644698	68647004-68647006	+	hsa_skin_006010	231	65	2	2308	GG	TET1	exon	B	no
chr17	40495986-40495987	40496520-40496521	-	hsa_skin_141959	90	46	2	534	G	TNS4	exon	B	no
chr2	23823256-23823259	23823568-23823571	-	hsa_skin_223319	60	35	2	312	AGG	ATAD2B	exon	B	no
chr2	40428470-40428472	40430299-40430301	-	hsa_skin_130041	134	60	2	1829	GT	SLC8A1	exon	B	no
chr17	83084936-83084937	83085322-83085323	+	hsa_skin_203265	203	79	2	386	G	METRNL	exon	B	no
chr17	73235473-73235476	73236993-73236996	+	hsa_skin_189139	66	40	2	1520	AGG	C17orf80	exon	B	no
chr5	73840477-73840481	73840758-73840762	+	hsa_skin_013327	125	47	2	281	AGGT	ARHGEF28	exon	B	no
chr10	68959803-68959805	68960247-68960249	+	hsa_skin_153897	243	86	2	444	AG	DDX21	exon	B	no
chr18	2890560	2892486	+	hsa_skin_172138	138	68	2	1926	-	EMILIN2	exon	B	no
chr14	99257470-99257473	99257839-99257842	-	hsa_skin_065509	326	124	2	369	CAG	BCL11B	exon	B	no
chr17	46170854	46172232	-	hsa_skin_039501	80	51	2	1378	-	AC217773.1; KANSL1	exon	B	no
chr5	66053403-66053406	66054949-66054952	+	hsa_skin_130930	737	154	2	1546	AGG	ERBB2IP	exon	B	no
chr5	177209633-177209637	177212193-177212197	+	hsa_skin_187603	86	46	2	2560	AGGT	NSD1	exon	B	no
chr1	28987428-28987430	28987903-28987905	+	hsa_skin_228015	53	34	2	475	AG	EPB41	exon	B	no
chr1	224189575-224189576	224190318-224190319	+	hsa_skin_039030	197	52	2	743	G	DEGS1	exon	B	no
chr1	151315572-151315574	151316509-151316511	-	hsa_skin_026246	120	51	2	937	AG	PI4KB	exon	B	no
chr4	186706562-186706565	186709845-186709848	-	hsa_skin_186947	251	114	2	3283	CAG	FAT1	exon	B	no
chr11	92352094-92352096	92355403-92355405	+	hsa_skin_112375	139	58	2	3309	GG	FAT3	exon	B	no
chr12	45926138-45926142	45928856-45928860	-	hsa_skin_069048	199	87	2	2718	GGTA	SCAF11	exon	B	no
chr8	127890586-127890589	127890996-127890999	+	hsa_skin_171562	123	54	2	410	AGG	PVT1; PVT1_3	exon	B	no
chr6	111177210-111177211	111177664-111177665	+	hsa_skin_019506	26	13	2	454	G	SLC16A10	exon	B	no
chr16	346146-346150	347105-347109	-	hsa_skin_213436	68	43	2	959	CAGG	AXIN1	exon	B	fold
chr4	177353304-177353307	177353725-177353728	+	hsa_skin_031743	70	38	2	421	CAG	NEIL3	exon	B	no

chrom	start	end	s	name	reads	uniq	ss	len	SHS	host_gene	anno	type	cs
chr12	14423906-14423908	14425471-14425473	+	hsa_skin_001100	142	63	2	1565	AG	ATF7IP	exon	B	no
chr4	36228580-36228583	36229644-36229647	-	hsa_skin_083065	2572	324	2	1064	AGG	ARAP2	exon	B	no
chr12	95208842-95208844	95211267-95211269	-	hsa_skin_192569	396	148	2	2425	AG	FGD6	exon	B	no
chr8	38429681-38429684	38429948-38429951	-	hsa_skin_194732	38	24	2	267	CAG	FGFR1	exon	B	no
chr22	32478979-32478980	32479274-32479275	+	hsa_skin_223866	98	49	2	295	G	FBXO7	exon	B	no
chr8	60741255-60741259	60743094-60743098	+	hsa_skin_218487	146	61	2	1839	CAGG	CHD7	exon	B	no
chr3	136001056-136001060	136003491-136003495	+	hsa_skin_174542	72	28	2	2435	AGGT	PPP2R3A	exon	B	no
chr17	20204329-20204333	20205909-20205913	+	hsa_skin_192421	398	120	2	1580	AAGG	AC004702.2; SPECC1	exon	B	fold
chr6	161048613-161048615	161049977-161049979	+	hsa_skin_198152	79	31	2	1364	AG	MAP3K4	exon	B	no
chr18	46890579-46890584	46891054-46891059	-	hsa_skin_121259	29	17	2	475	TTTAG	PIAS2	exon	B	no
chr6	134028379-134028380	134029720-134029721	-	hsa_skin_189369	51	26	2	1341	G	SLC2A12	exon	B	no
chr8	100287500-100287501	100288267-100288268	-	hsa_skin_102735	149	62	2	767	G	RNF19A	exon	B	no
chr4	109462899-109462902	109463643-109463646	+	hsa_skin_059513	51	26	2	744	GGT	SEC24B	exon	B	no
chr2	40428472	40430304	-	hsa_skin_192510	884	194	2	1832	-	SLC8A1	exon	B	no
chr2	207976650-207976652	207977586-207977588	-	hsa_skin_072281	410	112	2	936	AG	PLEKHM3	exon	B	no
chr18	44701172-44701174	44701830-44701832	+	hsa_skin_043278	150	62	2	658	AG	SETBP1	exon	B	no
chr6	75702639-75702644	75703067-75703072	+	hsa_skin_169713	160	56	2	428	TACAG	SENP6	exon	B	no
chr1	12275822-12275824	12278036-12278038	+	hsa_skin_184098	211	76	2	2214	AG	VPS13D	exon	B	no
chr10	45625950-45625951	45627105-45627106	-	hsa_skin_032293	29	16	2	1155	G	ZFAND4	exon	B	no
chr7	98191610-98191617	98194569-98194576	+	hsa_skin_175784	134	53	2	2959	CAGGTAT	LMTK2	exon	B	no
chr19	13025019-13025021	13025551-13025553	+	hsa_skin_231328	139	56	2	532	GG	NFIX	exon	B	no
chr12	82857009	82857580	+	hsa_skin_144913	212	85	2	571	-	TMTC2	exon	B	no
chr5	83537003-83537008	83542265-83542270	+	hsa_skin_130995	175	59	2	5262	CAGGT	VCAN	exon	B	no
chr13	52397231-52397234	52398194-52398197	-	hsa_skin_216799	79	38	2	963	CAG	THSD1	exon	B	no
chr10	124681606-124681609	124682379-124682382	-	hsa_skin_148616	320	114	2	773	CAG	FAM53B; RP11-12J10.3	exon	B	no
chr1	155853275-155853277	155853806-155853808	-	hsa_skin_206471	411	142	2	531	AG	GON4L	exon	B	no
chr6	4891710-4891714	4892377-4892381	+	hsa_skin_001947	1422	345	2	667	AGGT	CDYL	exon	B	no
chr12	128814772-128814775	128815068-128815071	-	hsa_skin_213580	92	46	2	296	GGT	SLC15A4	exon	B	no
chr17	77402057-77402058	77402702-77402703	+	hsa_skin_084383	173	73	2	645	G	9-Sep	exon	B	fold
chr13	30630832-30630834	30631472-30631474	+	hsa_skin_150608	41	31	2	640	AG	USPL1	exon	B	no
chr7	66240324	66241270	+	hsa_skin_203402	92	44	2	946	-	TPST1	exon	B	no
chr8	51860843-51860846	51861245-51861248	-	hsa_skin_034968	3525	381	2	402	AGG	PCMTD1	exon	B	no
chr5	61472679-61472682	61473036-61473039	+	hsa_skin_104582	144	64	2	357	GGT	ZSWIM6	exon	B	no
chr4	73090665-73090668	73092299-73092302	-	hsa_skin_189986	490	120	2	1634	AGG	ANKRD17	exon	B	no
chr17	37553113-37553117	37554058-37554062	-	hsa_skin_017912	52	26	2	945	CAGG	SYNRG	exon	B	fold

chrom	start	end	s	name	reads	uniq	ss	len	SHS	host_gene	anno	type	cs
chr19	48633442-48633446	48633654-48633658	-	hsa_skin_004930	101	53	2	212	AAGG	DBP	exon	B	no
chr1	200760837-200760838	200761097-200761098	+	hsa_skin_098650	38	21	2	260	G	CAMSAP2	exon	B	no
chr20	63202286-63202288	63203807-63203809	-	hsa_skin_185622	37	28	2	1521	AG	YTHDF1	exon	B	no
chr2	121605700-121605702	121606180-121606182	-	hsa_skin_164494	79	39	2	480	AG	CLASP1	exon	B	fold
chr7	102227365-102227366	102227668-102227669	+	hsa_skin_222254	101	48	2	303	G	CUX1	exon	B	no
chr14	50150006-50150010	50150229-50150233	-	hsa_skin_086492	329	146	2	223	CAGG	SOS2	exon	B	fold
chr1	24514311-24514313	24514565-24514567	+	hsa_skin_224575	260	88	2	254	AG	RCAN3	exon	B	no
chr8	101558416-101558418	101558810-101558812	+	hsa_skin_048041	368	101	2	394	AG	GRHL2	exon	B	no
chr14	32090499-32090502	32094384-32094387	+	hsa_skin_055982	246	57	2	3885	AGG	ARHGAP5	exon	B	no
chr22	40881767-40881770	40882175-40882178	+	hsa_skin_158920	59	37	2	408	AGG	XPNPEP3	exon	B	no
chr1	84865383-84865387	84866137-84866141	-	hsa_skin_165206	350	145	2	754	TAGG	LPAR3	exon	B	no
chr3	114350269-114350276	114351874-114351881	-	hsa_skin_209986	336	129	2	1605	CAGGTGA	ZBTB20	exon	B	no
chr8	27294077-27294080	27294308-27294311	-	hsa_skin_191860	139	82	2	231	GGT	TRIM35	exon	B	no
chr12	122340750-122340754	122341695-122341699	-	hsa_skin_183503	605	169	2	945	AGGT	CLIP1	exon	B	no
chr18	58918270	58920633	+	hsa_skin_115457	54	33	2	2363	-	ZNF532	exon	B	no
chr9	710801-710804	713462-713465	+	hsa_skin_135493	162	73	2	2661	AGG	KANK1	exon	B	no
chr1	155521099	155521618	-	hsa_skin_199419	67	36	2	519	-	ASH1L	exon	B	no
chr2	210153492-210153494	210154609-210154611	-	hsa_skin_090282	310	127	2	1117	GT	KANSL1L	exon	B	no
chr16	68121985-68121986	68123120-68123121	+	hsa_skin_090307	247	68	2	1135	G	NFATC3; RP11-67A1.2	exon	B	no
chr19	3623685-3623689	3624160-3624164	-	hsa_skin_156795	67	39	2	475	AGGT	CACTIN	exon	B	no
chr9	37126309-37126311	37126940-37126942	+	hsa_skin_051924	172	77	2	631	AG	ZCCHC7	exon	B	no
chr3	183650293-183650295	183651274-183651276	+	hsa_skin_229401	27	12	2	981	AG	KLHL24	exon	B	no
chr1	155438326-155438328	155439068-155439070	-	hsa_skin_119897	135	64	2	742	AG	ASH1L	exon	B	no
chrX	148661907	148662768	+	hsa_skin_165464	113	57	2	861	-	AFF2	exon	B	no
chr11	33286409-33286415	33287508-33287514	+	hsa_skin_080884	3914	476	2	1099	CAGGTA	HIPK3	exon	B	no
chr15	98707561	98708107	+	hsa_skin_215849	119	52	2	546	-	IGF1R	exon	B	no
chr4	87195323-87195325	87195690-87195692	-	hsa_skin_008217	147	67	2	367	AG	KLHL8	exon	B	no
chr14	71587569-71587570	71589369-71589370	+	hsa_skin_199788	102	46	2	1800	G	SIPA1L1	exon	B	no
chr16	53155923-53155926	53157539-53157542	+	hsa_skin_193893	184	52	2	1616	AGG	CHD9	exon	B	fold
chr2	32414764-32414769	32416159-32416164	+	hsa_skin_083378	160	71	2	1395	AAAGG	BIRC6	exon	B	no
chr17	19958012-19958013	19958570-19958571	-	hsa_skin_033567	76	37	2	558	G	AKAP10	exon	B	no
chr16	85633911-85633914	85634130-85634133	+	hsa_skin_117924	310	106	2	219	AGG	GSE1	exon	B	no
chr2	37316235-37316238	37317178-37317181	-	hsa_skin_050529	1075	170	2	943	AGG	PRKD3	exon	B	no
chr7	23611169-23611170	23611552-23611553	+	hsa_skin_208978	514	99	2	383	G	CCDC126	exon	B	no
chr14	34862043-34862045	34862322-34862324	-	hsa_skin_062816	148	78	2	279	AG	BAZ1A	exon	B	no

chrom	start	end	s	name	reads	uniq	ss	len	SHS	host_gene	anno	type	cs
chr16	69695135	69695379	+	hsa_skin_057128	109	43	2	244	-	NFAT5	exon	B	no
chr2	64551439-64551444	64553406-64553411	+	hsa_skin_154906	188	64	2	1967	CAGGT	AFTPH	exon	B	no
chr9	34241183-34241184	34242107-34242108	+	hsa_skin_156445	52	35	2	924	G	UBAP1	exon	B	no
chr8	37877105-37877109	37877548-37877552	-	hsa_skin_105527	114	61	2	443	GGTG	RAB11FIP1	exon	B	no
chr6	106519129-106519130	106521452-106521453	+	hsa_skin_056888	78	37	2	2323	G	AIM1	exon	B	no
chr14	75046374-75046376	75049717-75049719	-	hsa_skin_025687	260	92	2	3343	GG	MLH3	exon	B	no
chr18	32287028-32287030	32288201-32288203	-	hsa_skin_139144	40	26	2	1173	GT	GAREM	exon	B	no
chr22	20933776-20933781	20934242-20934247	+	hsa_skin_171883	106	53	2	466	AGGTA	CRKL	exon	B	no
chr15	101235081-101235083	101235577-101235579	-	hsa_skin_186182	109	53	2	496	AG	CHSY1	exon	B	no
chr9	112574054-112574057	112575249-112575252	+	hsa_skin_105944	51	38	2	1195	AGG	KIAA1958	exon	B	no
chr14	64521484-64521488	64523400-64523404	+	hsa_skin_001742	90	46	2	1916	AGGT	ZBTB1	exon	B	no
chr15	41668826-41668828	41669957-41669959	+	hsa_skin_023883	775	152	2	1131	GG	MGA	exon	B	no
chr6	16326393-16326398	16328470-16328475	-	hsa_skin_127186	114	66	2	2077	CCCAG	ATXN1	exon	B	no
chr16	3850296-3850298	3851009-3851011	-	hsa_skin_048346	286	121	2	713	AG	CREBBP	exon	B	no
chr17	59353212-59353215	59353524-59353527	+	hsa_skin_039912	138	57	2	312	AGG	YPEL2	exon	B	no
chr12	116230531-116230535	116237704-116237708	-	hsa_skin_129279	56	33	2	7173	CAGG	MED13L	intron-exon	5' B	no
chr7	139715928-139715933	139717012-139717017	-	hsa_skin_232176	110	65	2	1084	AGGTA	HIPK2	intron-exon	5' B	no
chr10	20957728-20957731	20961777-20961780	-	hsa_skin_108592	46	25	2	4049	GGT	NEBL	intron-exon	5' B	no
chr12	109852141-109852147	109853437-109853443	-	hsa_skin_063495	37	19	0	1296	GCTGGG	GLTP	exon	I	fold
chr11	119068010-119068014	119069195-119069199	+	hsa_skin_132621	15	8	0	1185	ATAT	VPS11	intron	B	no
chr1	92836390-92836392	92837453-92837455	+	hsa_skin_060951	22	14	0	1063	AG	RPL5; SNORD21	intron	B	no
chr16	53141174-53141178	53157537-53157541	+	hsa_skin_119115	322	82	2	16363	ACAG	CHD9	intron-exon	3' B	no
chr11	36227082-36227084	36227428-36227430	+	hsa_skin_176158	197	70	2	346	AG	LDLRAD3	intron-exon	3' B	no
chr9	33293676-33293680	33295424-33295428	+	hsa_skin_044188	128	58	2	1748	CAGG	NFX1	intron-exon	3' B	no
chr5	36982162-36982165	36986299-36986302	+	hsa_skin_017868	181	45	2	4137	AGG	NIPBL	intron-exon	3' B	no
chr11	19159809-19159815	19160675-19160681	+	hsa_skin_120213	96	36	0	866	GGTTAC	ZDHHC13	intron	I	fold
chr5	158941693-158941696	158941979-158941982	-	hsa_skin_203753	112	42	2	286	CAG	EBF1	intron	I	no
chr7	99493037-99493042	99494629-99494634	-	hsa_skin_112754	308	108	2	1592	CAGGT	ZNF394	intron	I	no
chr8	141253988-141253989	141254629-141254630	-	hsa_skin_124307	390	141	2	641	G	SLC45A4	intron	I	no
chr6	158312048-158312050	158314266-158314268	+	hsa_skin_043418	1363	240	2	2218	AG	RP11-732M18.4; TULP4	intron	I	no
chr11	3811755-3811759	3812083-3812087	+	hsa_skin_100269	41	26	0	328	TGTC	PGAP2	intron	I	fold
chr20	43587212-43587218	43587378-43587384	+	hsa_skin_020512	20	9	0	166	GCTGGG	SGK2	intron	I	fold
chr2	113707872-113707876	113708437-113708441	-	hsa_skin_143837	24	15	0	565	TGAG	SLC35F5	intron	I	fold
chr22	24573141-24573145	24574592-24574596	+	hsa_skin_201121	69	23	0	1451	TGCA	SNRPD3	intron	I	fold
chr20	62276203-62276204	62276962-62276963	+	hsa_skin_130118	32	15	0	759	A	OSBPL2	intron	I	fold

chrom	start	end	s	name	reads	uniq	ss	len	SHS	host_gene	anno	type	cs
chr20	3218514-3218518	3218992-3218996	+	hsa_skin_137810	47	24	2	478	AGGT	ITPA	exon	5' B	no
chr10	92450736-92450738	92451257-92451259	-	hsa_skin_228653	25	15	0	521	GA	-	intergenic	I	no
chr10	92450725-92450733	92451246-92451254	-	hsa_skin_098241	57	27	0	521	CAGGCATG	-	intergenic	I	fold
chr4	86822780-86822784	86823096-86823100	-	hsa_skin_081592	87	40	0	316	CATG	-	intergenic	I	fold
chr19	52383847-52383848	52385323-52385324	+	hsa_skin_102568	25	13	2	1476	G	ZNF880	exon	5' B	no
chr6	106568470-106568472	106568970-106568972	+	hsa_skin_188406	47	29	2	500	GG	AIM1	exon	5' B	no
chr9	4860124-4860125	4860901-4860902	+	hsa_skin_050166	118	51	2	777	G	RCL1	exon	5' B	no
chr9	127444025-127444029	127445246-127445250	+	hsa_skin_028220	49	26	2	1221	CAGG	ZNF79	exon	5' B	fold
chr17	40818300-40818303	40818481-40818484	-	hsa_skin_108296	142	51	0	181	AGA	KRT10	exon	5' B	no
chr17	76007059-76007062	76010543-76010546	-	hsa_skin_194492	81	52	2	3484	CAG	EVPL	exon	5' B	no
chr14	89411932-89411935	89412489-89412492	-	hsa_skin_093345	88	61	2	557	AGG	FOXN3; RP11-33N16.3	exon	5' B	no
chr16	16141172-16141173	16142942-16142943	+	hsa_skin_146560	54	24	2	1770	G	ABCC1	exon	5' B	no
chr11	130260854-130260858	130261928-130261932	-	hsa_skin_102236	1169	240	2	1074	TAGG	ZBTB44	exon	5' B	no
chr15	64499290-64499292	64500164-64500166	+	hsa_skin_164127	1378	328	2	874	AG	ZNF609	exon	5' B	no
chr12	1027744-1027747	1028569-1028572	+	hsa_skin_183280	470	151	2	825	CAG	ERC1	exon	5' B	no
chr15	72045723-72045725	72046634-72046636	-	hsa_skin_173421	140	61	2	911	AG	MYO9A	exon	5' B	no
chr19	52383847-52383848	52385741-52385742	+	hsa_skin_220483	58	27	2	1894	G	ZNF880	exon	5' B	no
chr6	89083752-89083754	89084643-89084645	+	hsa_skin_184272	245	82	2	891	GT	PNRC1	exon	5' B	no
chr19	58260847-58260850	58263673-58263676	+	hsa_skin_039493	159	82	2	2826	CAG	ZNF544	exon	5' B	no
chr17	40818151-40818157	40818475-40818481	-	hsa_skin_214394	78	15	0	324	TACTAA	KRT10	exon	5' B	no
chr17	7576810	7576951	+	hsa_skin_036960	31	6	0	141	-	EIF4A1; RP11-186B7.4; SENP3-EIF4A1; SNORD10	exon	5' B	no
chr19	23358426-23358431	23362722-23362727	-	hsa_skin_107727	903	166	2	4296	AGGTA	ZNF91	exon	5' B	no
chr18	75285445-75285447	75288646-75288648	+	hsa_skin_010497	28	16	2	3201	AG	TSHZ1	exon	5' B	no

Table S3 Genes significantly differentially expressed between PP and NN

Only genes with fold change >= 2 or <= -2, and q_value <= 0.01 are listed due to the large amount.

gene_id	gene	locus	NN	PP	log2(fold_change)	p_value	q_value
XLOC_060937	DEFB4A	chr8:7894628-7896711	0.49	1552.09	11.62	0.00	0.00
XLOC_002649	IL19	chr1:206767601-206842981	0.00	6.88	10.73	0.00	0.00
XLOC_004974	SPRR2F	chr1:153092172-153150872	0.39	521.29	10.39	0.00	0.00
XLOC_034142	IL36A	chr2:113005306-113012244	0.03	29.27	10.10	0.00	0.00
XLOC_001909	S100A7A	chr1:153416523-153423225	0.47	271.72	9.16	0.00	0.00
XLOC_021769	-	chr15:32700980-32701376	0.01	3.24	9.13	0.00	0.00
XLOC_004971	SPRR2A	chr1:153056112-153057537	4.07	2143.20	9.04	0.00	0.00
XLOC_004976	SPRR2C	chr1:153092172-153150872	0.05	16.98	8.45	0.00	0.00
XLOC_011558	TCN1	chr11:59852799-59866575	0.28	93.19	8.40	0.00	0.00
XLOC_038755	PI3	chr20:45174875-45176544	7.43	2067.88	8.12	0.00	0.00
XLOC_035681	-	chr2:10902893-10903530	0.00	1.21	8.12	0.00	0.00
XLOC_004992	S100A8	chr1:153390031-153391188	86.70	23819.90	8.10	0.00	0.00
XLOC_004955	LCE3A	chr1:152622600-152624052	0.92	239.97	8.03	0.00	0.00
XLOC_001907	S100A9	chr1:153357853-153361027	83.81	21838.20	8.03	0.00	0.00
XLOC_047028	CXCL8	chr4:73740505-73743716	0.10	25.50	7.94	0.00	0.00
XLOC_004972	SPRR2B	chr1:153070223-153072162	3.86	945.43	7.93	0.00	0.00
XLOC_004989	S100A12	chr1:153373705-153375649	0.28	68.02	7.93	0.00	0.00
XLOC_057190	AGPAT4	chr6:160990317-161288561	0.01	1.23	7.91	0.00	0.00
XLOC_049116	TNIP3	chr4:121129120-121227466	0.02	5.96	7.91	0.00	0.00
XLOC_032360	CTC-490G23.2	chr19:43329294-43331430	0.01	2.94	7.85	0.00	0.00
XLOC_009616	-	chr11:47910012-47910294	0.02	5.17	7.81	0.00	0.00
XLOC_004997	S100A7	chr1:153457743-153460701	64.05	13102.10	7.68	0.00	0.00
XLOC_010374	-	chr11:94115643-94128772	0.01	1.47	7.55	0.00	0.00
XLOC_001898	SPRR3	chr1:152984035-153032902	0.01	1.84	7.53	0.00	0.00
XLOC_029819	SERPINB3, SERPINB4	chr18:63634977-63726432	9.61	1630.12	7.41	0.00	0.00
XLOC_022582	RHCG	chr15:89471397-89496613	1.10	158.71	7.17	0.00	0.00
XLOC_048635	TMPRSS11D	chr4:67701213-68133166	0.16	23.07	7.17	0.00	0.00
XLOC_010373	HEPHL1	chr11:94021360-94113751	0.46	65.84	7.15	0.00	0.00
XLOC_056564	-	chr6:106860623-106872275	0.01	0.75	7.10	0.00	0.01
XLOC_015050	KRT6A	chr12:52446650-52493257	1.44	192.10	7.06	0.00	0.00
XLOC_002650	IL20	chr1:206863384-206869389	0.02	3.01	7.00	0.00	0.00
XLOC_023603	CNGB1	chr16:57881681-57971116	0.01	1.39	6.69	0.00	0.01
XLOC_021139	-	chr15:70190114-70302720	0.02	2.06	6.66	0.00	0.00
XLOC_024043	CTD-2555A7.2	chr16:89046171-89059251	0.01	0.74	6.65	0.00	0.00
XLOC_056796	VNN3	chr6:132720559-132734765	0.07	6.56	6.64	0.00	0.00

**gene_id**	**gene**	**locus**	**NN**	**PP**	**log2(fold_change)**	**p_value**	**q_value**
XLOC_022581	-	chr15:89451829-89470044	0.02	2.28	6.61	0.00	0.00
XLOC_056030	ADGRF1	chr6:46997702-47042363	0.02	1.79	6.38	0.00	0.00
XLOC_027384	KRT16P3	chr17:20449394-20699914	0.01	0.78	6.38	0.00	0.00
XLOC_003952	CYP4Z2P	chr1:46749643-47180339	0.02	1.71	6.36	0.00	0.00
XLOC_004969	SPRR2D	chr1:153039724-153041931	15.08	1217.52	6.33	0.00	0.00
XLOC_022268	-	chr15:66887508-66896506	0.02	1.90	6.33	0.00	0.00
XLOC_029101	SERPINB11	chr18:63634977-63726432	6.24	485.71	6.28	0.00	0.00
XLOC_059615	C7orf57	chr7:48035510-48061304	0.03	2.33	6.27	0.00	0.00
XLOC_004953	LCE3E	chr1:152510745-152601086	4.81	361.25	6.23	0.00	0.00
XLOC_047035	CXCL1	chr4:73869392-73871242	0.14	10.41	6.22	0.00	0.00
XLOC_048636	TMPRSS11A	chr4:67701213-68133166	0.01	0.99	6.20	0.00	0.00
XLOC_025752	KRT16P1	chr17:18432050-18442994	0.04	2.62	6.04	0.00	0.00
XLOC_040700	CLDN17	chr21:30164327-30167091	0.07	4.57	6.02	0.00	0.00
XLOC_020708	CHAC1	chr15:40952961-40957811	0.30	18.79	5.96	0.00	0.00
XLOC_061159	ADAMDEC1	chr8:24294039-24912073	0.02	1.35	5.93	0.00	0.00
XLOC_004954	LCE3D	chr1:152510745-152601086	16.39	994.48	5.92	0.00	0.00
XLOC_030897	CD177	chr19:43353658-43368970	0.04	2.19	5.91	0.00	0.00
XLOC_012170	PHBP16	chr11:94021360-94113751	0.02	0.96	5.89	0.00	0.00
XLOC_016927	-	chr13:20330950-20402282	0.02	0.97	5.88	0.00	0.00
XLOC_029818	SERPINB4	chr18:63634977-63726432	1.90	109.49	5.85	0.00	0.00
XLOC_065058	LCN2	chr9:128149070-128153455	3.46	192.21	5.80	0.00	0.00
XLOC_058723	AKR1B10	chr7:134527557-134541549	2.11	111.91	5.73	0.00	0.00
XLOC_008516	-	chr10:84166690-84172816	0.03	1.59	5.71	0.00	0.00
XLOC_015051	KRT6C	chr12:52446650-52493257	6.53	305.72	5.55	0.00	0.00
XLOC_055798	-	chr6:31853374-31853595	0.06	2.81	5.52	0.00	0.00
XLOC_043188	-	chr3:71897133-71899275	0.03	1.36	5.52	0.00	0.01
XLOC_058724	AKR1B15	chr7:134549106-134579875	0.08	3.67	5.44	0.00	0.00
XLOC_034386	KYNU	chr2:142877276-143055832	0.82	34.43	5.40	0.00	0.00
XLOC_015052	KRT6A	chr12:52446650-52493257	100.67	4023.08	5.32	0.00	0.00
XLOC_030979	IGFL1	chr19:46078815-46250270	0.66	25.51	5.27	0.00	0.00
XLOC_016176	ATP12A	chr13:24680410-24716604	1.87	71.45	5.26	0.00	0.00
XLOC_015873	OASL	chr12:121017487-121039242	0.47	17.01	5.19	0.00	0.00
XLOC_018812	IFI27	chr14:94104488-94116725	34.16	1192.60	5.13	0.00	0.00
XLOC_057827	AC004870.5	chr7:46890624-47079128	0.03	1.15	5.11	0.00	0.00
XLOC_010607	ABCG4	chr11:119149011-119162654	0.08	2.72	5.10	0.00	0.00
XLOC_044806	LTF	chr3:46435520-46485234	1.60	54.26	5.08	0.00	0.00
XLOC_032223	-	chr19:38797508-38798775	0.15	5.15	5.08	0.00	0.00
XLOC_064440	GDA	chr9:72114594-72257193	0.50	17.01	5.08	0.00	0.00

gene_id	gene	locus	NN	PP	log2(fold_change)	p_value	q_value
XLOC_005658	REN	chr1:204153763-204166322	0.06	1.86	5.05	0.00	0.00
XLOC_012228	MMP1	chr11:102746967-102843803	0.03	0.90	5.04	0.00	0.00
XLOC_027642	CCL3L3	chr17:36116176-36264553	0.06	1.87	5.02	0.00	0.00
XLOC_016925	GJB2	chr13:20184110-20192967	31.36	981.98	4.97	0.00	0.00
XLOC_007122	C10orf99	chr10:84173717-84190044	7.49	229.47	4.94	0.00	0.00
XLOC_068139	SLC6A14	chrX:116436443-116461556	3.05	92.37	4.92	0.00	0.00
XLOC_038245	TGM6	chr20:2380870-2435307	0.07	2.11	4.91	0.00	0.00
XLOC_062363	DEFB103B	chr8:7426692-7430348	0.05	1.23	4.77	0.00	0.00
XLOC_039864	HRH3	chr20:62214962-62221841	0.04	0.97	4.76	0.00	0.00
XLOC_035394	CCL20	chr2:227813841-227817564	0.48	12.83	4.75	0.00	0.00
XLOC_014140	CCDC60	chr12:119334711-119682273	0.03	0.91	4.75	0.00	0.00
XLOC_044493	SLC6A11	chr3:10816026-10941067	0.04	0.93	4.71	0.00	0.00
XLOC_026069	CCL4L2	chr17:36116176-36264553	0.08	2.06	4.70	0.00	0.00
XLOC_009487	RP4-743O11.1	chr11:34727049-34754578	0.14	3.65	4.70	0.00	0.00
XLOC_058598	HYAL4	chr7:123814138-123877823	0.32	7.91	4.64	0.00	0.00
XLOC_034086	-	chr2:110321522-110345715	0.06	1.41	4.63	0.00	0.00
XLOC_021135	RNU6-745P	chr15:70190114-70302720	0.13	3.29	4.63	0.00	0.00
XLOC_055320	RP4-529N6.2	chr6:4599286-4602420	0.51	12.24	4.58	0.00	0.00
XLOC_062160	-	chr8:140646162-140646439	0.36	8.65	4.58	0.00	0.00
XLOC_004973	SPRR2E, SPRR2G	chr1:153092172-153150872	179.67	4251.19	4.56	0.00	0.00
XLOC_024190	PRSS27	chr16:2712417-2720803	1.85	43.16	4.55	0.00	0.00
XLOC_036791	-	chr2:110042108-110050039	0.06	1.33	4.49	0.00	0.00
XLOC_001548	CHI3L2	chr1:111117332-111243440	5.36	119.70	4.48	0.00	0.00
XLOC_027302	KRT16P2, KRT16P6	chr17:16817982-16832830	0.34	7.60	4.47	0.00	0.00
XLOC_026064	CCL4	chr17:36103589-36105621	0.12	2.61	4.45	0.00	0.00
XLOC_027640	CCL3	chr17:36072717-36091012	0.16	3.43	4.45	0.00	0.00
XLOC_032222	RNU6-140P	chr19:38791952-38797411	0.04	0.86	4.41	0.00	0.00
XLOC_024199	PRSS22	chr16:2852726-2859726	2.04	43.33	4.41	0.00	0.00
XLOC_009071	KRTAP5-AS1	chr11:1554043-1600142	0.04	0.84	4.40	0.00	0.00
XLOC_030899	TEX101	chr19:43395738-43418597	0.07	1.47	4.39	0.00	0.00
XLOC_010523	HTR3A	chr11:113974880-113990313	0.32	6.56	4.34	0.00	0.00
XLOC_032578	KLK6	chr19:50941230-51020175	4.27	84.52	4.31	0.00	0.00
XLOC_042476	CTA-384D8.35	chr22:50542058-50547852	0.28	5.34	4.27	0.00	0.00
XLOC_020660	-	chr15:38610131-38651319	0.10	1.95	4.27	0.00	0.00
XLOC_048733	CXCL9	chr4:75910871-76007488	0.20	3.83	4.25	0.00	0.00
XLOC_047038	EPGN	chr4:74308223-74323535	0.67	12.55	4.23	0.00	0.00
XLOC_056815	RP11-557H15.3	chr6:134428037-134524383	0.14	2.61	4.20	0.00	0.00

gene_id	gene	locus	NN	PP	log2(fold_change)	p_value	q_value
XLOC_001897	SPRR1A, SPRR1B	chr1:152984035-153032902	78.04	1421.88	4.19	0.00	0.00
XLOC_042059	-	chr22:24790469-24790786	0.20	3.69	4.18	0.00	0.00
XLOC_039828	ZBP1	chr20:57603845-57620576	0.10	1.84	4.18	0.00	0.00
XLOC_041822	CTA-384D8.31, CTA-384D8.34	chr22:50542058-50547852	0.20	3.62	4.17	0.00	0.00
XLOC_056794	VNN1	chr6:132681467-132714049	0.20	3.54	4.16	0.00	0.00
XLOC_042972	CAMP	chr3:48223346-48225491	0.06	1.13	4.16	0.00	0.00
XLOC_063840	CD274	chr9:5439195-5833117	0.35	6.32	4.16	0.00	0.00
XLOC_018601	LINC01269	chr14:70681937-70714872	0.10	1.69	4.14	0.00	0.00
XLOC_057834	UPP1	chr7:48088627-48108734	2.07	36.32	4.13	0.00	0.00
XLOC_019921	KCNK10	chr14:88175390-88327481	0.09	1.58	4.08	0.00	0.00
XLOC_052738	PITX1	chr5:135027341-135401296	0.88	14.11	4.01	0.00	0.00
XLOC_021937	PLA2G4D	chr15:42064650-42094651	7.94	125.89	3.99	0.00	0.00
XLOC_065918	TJP2	chr9:69121110-69255208	0.07	1.15	3.97	0.00	0.00
XLOC_004946	HRNR	chr1:152122533-152445456	0.54	8.41	3.97	0.00	0.00
XLOC_041561	APOL1	chr22:36253009-36267530	0.99	15.40	3.96	0.00	0.00
XLOC_000926	FAAHP1	chr1:46423797-46482493	0.12	1.80	3.96	0.00	0.00
XLOC_042144	OSM	chr22:30262754-30266911	0.08	1.24	3.95	0.00	0.00
XLOC_003606	IFI6	chr1:27666060-27703063	17.77	273.41	3.94	0.00	0.00
XLOC_040936	-	chr21:43300384-43303838	0.08	1.21	3.93	0.00	0.01
XLOC_041823	KLHDC7B	chr22:50547966-50551023	0.09	1.30	3.92	0.00	0.00
XLOC_019275	GZMB	chr14:24595722-24657774	0.28	4.22	3.92	0.00	0.00
XLOC_019941	CTD-2547L24.4	chr14:91255833-91259003	0.08	1.13	3.90	0.00	0.01
XLOC_000460	EPHB2	chr1:22710838-22921500	0.63	9.37	3.89	0.00	0.00
XLOC_007932	ENKUR	chr10:24843535-25062477	0.07	0.97	3.89	0.00	0.00
XLOC_015049	KRT6B	chr12:52446650-52493257	59.80	866.90	3.86	0.00	0.00
XLOC_011676	BATF2	chr11:64987847-64997211	0.15	2.22	3.85	0.00	0.00
XLOC_012230	MMP3	chr11:102746967-102843803	0.05	0.74	3.85	0.00	0.00
XLOC_061891	LINC01181	chr8:103121031-103335268	0.35	5.02	3.84	0.00	0.00
XLOC_051335	HRH2	chr5:175657798-175719643	0.55	7.80	3.84	0.00	0.00
XLOC_051305	CTB-33O18.1	chr5:173561391-173574283	0.08	1.09	3.83	0.00	0.00
XLOC_000038	ISG15	chr1:1001137-1014541	4.13	58.53	3.83	0.00	0.00
XLOC_014070	OAS2	chr12:112906776-113017751	1.40	19.79	3.82	0.00	0.00
XLOC_025753	LGALS9C	chr17:18450243-18494945	0.15	2.19	3.82	0.00	0.00
XLOC_031586	FUT3	chr19:5842179-5858239	0.73	10.13	3.80	0.00	0.00
XLOC_021542	ALDH1A3	chr15:100849560-101086066	1.19	16.51	3.79	0.00	0.00
XLOC_057742	TRG-AS1	chr7:38239579-38378804	0.14	1.94	3.77	0.00	0.00
XLOC_014540	CLEC7A	chr12:10116776-10130258	2.69	36.49	3.76	0.00	0.00

gene_id	gene	locus	NN	PP	log2(fold_change)	p_value	q_value
XLOC_055039	RP11-350J20.12	chr6:149724314-149923121	3.82	51.75	3.76	0.00	0.00
XLOC_032585	KLK13	chr19:51055405-51069556	5.03	67.54	3.75	0.00	0.00
XLOC_048734	CXCL10	chr4:76011183-76112802	0.76	10.04	3.73	0.00	0.00
XLOC_046459	S100P	chr4:6693068-6697170	6.19	82.19	3.73	0.00	0.00
XLOC_035156	ADAM23	chr2:206443503-206621277	0.26	3.48	3.73	0.00	0.00
XLOC_007779	PRKCQ	chr10:6394335-6616452	0.18	2.41	3.72	0.00	0.00
XLOC_008577	HTR7	chr10:90739646-90857865	0.09	1.23	3.72	0.00	0.00
XLOC_001268	IFI44,IFI44L	chr1:78619921-78664373	3.36	43.60	3.70	0.00	0.00
XLOC_040307	MX1	chr21:41420303-41459214	4.62	59.82	3.70	0.00	0.00
XLOC_049593	-	chr4:183329999-183350835	0.10	1.28	3.69	0.00	0.00
XLOC_042474	ODF3B, SCO2, TYMP	chr22:50523567-50532580	29.46	366.53	3.64	0.00	0.00
XLOC_005986	PGBD5	chr1:230314454-230425729	0.38	4.65	3.63	0.00	0.00
XLOC_032916	RSAD2	chr2:6840569-6898239	0.68	8.34	3.63	0.00	0.00
XLOC_027254	HS3ST3A1	chr17:13492712-13601997	0.26	3.17	3.61	0.00	0.00
XLOC_048735	CXCL11	chr4:76011183-76112802	0.07	0.88	3.59	0.00	0.00
XLOC_055724	TRIM10	chr6:30151882-30172696	0.12	1.49	3.58	0.00	0.00
XLOC_064447	TMC1	chr9:72521800-72836351	0.10	1.16	3.57	0.00	0.00
XLOC_011555	FABP5P7	chr11:59712822-59806024	0.62	7.34	3.57	0.00	0.00
XLOC_016926	GJB6	chr13:20221960-20233032	28.10	332.89	3.57	0.00	0.00
XLOC_039903	SRMS	chr20:63538369-63547736	0.29	3.33	3.51	0.00	0.00
XLOC_067067	-	chrX:7040872-7446415	0.16	1.79	3.50	0.00	0.00
XLOC_030773	PAPL	chr19:38990713-39117070	6.02	67.73	3.49	0.00	0.00
XLOC_036838	HMGN2P23	chr2:112882045-112985723	0.23	2.61	3.48	0.00	0.00
XLOC_061670	FABP5	chr8:81279870-81284777	181.21	2018.80	3.48	0.00	0.00
XLOC_048951	ADH7	chr4:99412233-99438578	0.32	3.47	3.46	0.00	0.00
XLOC_006343	PRKCQ-AS1	chr10:6394335-6616452	0.40	4.40	3.46	0.00	0.00
XLOC_062782	PLAT	chr8:42175232-42271263	2.62	28.53	3.45	0.00	0.00
XLOC_002556	ELF3	chr1:201982371-202017188	2.04	22.26	3.45	0.00	0.00
XLOC_005230	ADAMTS4	chr1:161184252-161214468	0.16	1.77	3.45	0.00	0.00
XLOC_001892	-	chr1:152859986-152861423	0.33	3.48	3.42	0.00	0.00
XLOC_067049	ARSF	chrX:3041372-3112891	1.74	18.47	3.40	0.00	0.00
XLOC_062713	LINC01605, RP11-150O12.6, RP11-527N22.2	chr8:37403374-37554183	0.86	9.07	3.39	0.00	0.00
XLOC_005162	AIM2	chr1:159062483-159147096	0.13	1.36	3.39	0.00	0.00
XLOC_010572	RP11-832A4.7	chr11:118264540-118268506	0.19	1.96	3.38	0.00	0.00
XLOC_003777	RP11-334L9.1	chr1:38568805-38728578	0.25	2.62	3.38	0.00	0.00
XLOC_063326	BAALC-AS2	chr8:103121031-103335268	0.12	1.20	3.37	0.00	0.00

gene_id	gene	locus	NN	PP	log2(fold_change)	p_value	q_value
XLOC_000733	ZC3H12A	chr1:37453001-37484563	10.44	107.64	3.37	0.00	0.00
XLOC_064710	FOXE1	chr9:97853124-97860370	0.64	6.58	3.37	0.00	0.00
XLOC_018782	CTD-2547L24.4	chr14:91255833-91259003	0.14	1.45	3.36	0.00	0.00
XLOC_032340	CXCL17	chr19:42397127-42740569	0.08	0.78	3.34	0.00	0.00
XLOC_062656	LINC00589	chr8:29667485-29748109	0.07	0.71	3.34	0.00	0.00
XLOC_046759	CHRNA9	chr4:40334989-40355304	0.55	5.56	3.33	0.00	0.00
XLOC_021415	MESP2	chr15:89760590-89778754	0.16	1.57	3.31	0.00	0.00
XLOC_001339	GBP6	chr1:89363890-89427806	0.97	9.54	3.30	0.00	0.00
XLOC_027793	KRT17	chr17:41619436-41624842	201.69	1980.40	3.30	0.00	0.00
XLOC_010570	TMPRSS4	chr11:118015771-118121890	1.73	16.98	3.29	0.00	0.00
XLOC_005111	NES	chr1:156668762-156677397	1.02	9.98	3.29	0.00	0.00
XLOC_055044	ULBP2	chr6:149941988-149949235	0.14	1.40	3.28	0.00	0.00
XLOC_043320	GPR15	chr3:98497603-98593723	0.08	0.77	3.25	0.00	0.00
XLOC_021204	CPLX3, LMAN1L	chr15:74812715-74831821	0.14	1.26	3.20	0.00	0.00
XLOC_050969	SPATA24	chr5:139390591-139404088	0.22	1.97	3.18	0.00	0.00
XLOC_045427	-	chr3:124749563-124751229	0.26	2.30	3.16	0.00	0.00
XLOC_053802	TRIM15	chr6:30151882-30172696	0.33	2.93	3.16	0.00	0.00
XLOC_038781	MMP9	chr20:46008907-46089971	0.68	6.03	3.15	0.00	0.00
XLOC_032318	AC005626.3	chr19:41630960-41641861	0.24	2.09	3.15	0.00	0.00
XLOC_022886	IL32	chr16:3065296-3087100	2.07	18.09	3.13	0.00	0.00
XLOC_067065	-	chrX:7040872-7446415	0.18	1.58	3.12	0.00	0.00
XLOC_008261	SLC16A9	chr10:59650760-59736002	0.17	1.45	3.12	0.00	0.00
XLOC_004391	GBP5	chr1:89256194-89273057	0.33	2.88	3.12	0.00	0.00
XLOC_031833	PGLYRP2	chr19:15468644-15498956	0.15	1.31	3.11	0.00	0.00
XLOC_029516	DSC2	chr18:31058839-31163061	10.20	87.61	3.10	0.00	0.00
XLOC_019264	-	chr14:24243401-24244191	0.12	1.05	3.09	0.00	0.00
XLOC_018818	SERPINA3, SERPINA4, SERPINA5	chr14:94561090-94624646	3.46	29.22	3.08	0.00	0.00
XLOC_061996	FAM83A	chr8:123178863-123210471	2.04	17.12	3.07	0.00	0.00
XLOC_044181	RTP4	chr3:187368331-187372423	0.39	3.22	3.06	0.00	0.00
XLOC_044997	FAM3D	chr3:58564085-58666848	0.70	5.87	3.06	0.00	0.00
XLOC_005003	S100A2, S100A3	chr1:153543612-153567890	103.96	861.89	3.05	0.00	0.00
XLOC_005368	-	chr1:172531729-172532344	0.22	1.80	3.03	0.00	0.00
XLOC_019972	SERPINA1	chr14:94376746-94390693	0.71	5.80	3.03	0.00	0.00
XLOC_037052	CXCR4	chr2:136114348-136118165	0.98	7.98	3.03	0.00	0.00
XLOC_035630	CMPK2	chr2:6840569-6898239	0.89	7.24	3.02	0.00	0.00
XLOC_005687	SLC26A9	chr1:205912922-205943460	1.04	8.46	3.02	0.00	0.00
XLOC_040892	AP001610.5	chr21:41420303-41459214	0.10	0.81	3.02	0.00	0.00

gene_id	gene	locus	NN	PP	log2(fold_change)	p_value	q_value
XLOC_047173	HERC6	chr4:88378738-88443111	2.28	18.47	3.02	0.00	0.00
XLOC_054041	MDFI	chr6:41636881-41654501	3.30	26.37	3.00	0.00	0.00
XLOC_012575	LINC01395, RP11-507F16.1	chr11:129591492-129815147	0.27	2.19	3.00	0.00	0.00
XLOC_036904	-	chr2:120227406-120232462	0.11	0.84	2.99	0.00	0.00
XLOC_047365	RP11-384K6.6	chr4:118591752-118633893	0.96	7.60	2.98	0.00	0.00
XLOC_059973	AC073850.6	chr7:80312573-80682610	0.40	3.09	2.96	0.00	0.00
XLOC_048700	CXCL2	chr4:74097034-74099293	0.19	1.49	2.95	0.00	0.00
XLOC_027381	LGALS9B	chr17:20449394-20699914	0.40	3.07	2.94	0.00	0.00
XLOC_009395	LUZP2	chr11:24496710-25082879	0.23	1.72	2.92	0.00	0.00
XLOC_057471	AC011288.2	chr7:13101390-13704149	0.33	2.47	2.92	0.00	0.00
XLOC_038976	CDH26	chr20:59958331-60034460	0.24	1.80	2.91	0.00	0.00
XLOC_019011	CTD-2555C10.3	chr14:102545253-102558930	0.22	1.65	2.91	0.00	0.00
XLOC_008067	NAMPTP1	chr10:36521720-36524234	0.72	5.36	2.89	0.00	0.00
XLOC_027143	RP11-609D21.3	chr17:6755432-6776116	2.19	16.25	2.89	0.00	0.00
XLOC_005114	CRABP2	chr1:156699605-156705816	61.56	448.20	2.86	0.00	0.00
XLOC_019940	GPR68	chr14:90845874-91253925	3.25	23.51	2.86	0.00	0.00
XLOC_044990	DNASE1L3	chr3:58192107-58214697	1.74	12.60	2.86	0.00	0.00
XLOC_056566	CD24	chr6:106969830-106975627	57.77	414.99	2.84	0.00	0.00
XLOC_002247	FMO2	chr1:171167182-171286274	0.76	5.46	2.84	0.00	0.00
XLOC_050336	TMEM171	chr5:73120291-73131817	0.52	3.72	2.83	0.00	0.00
XLOC_036833	IL1B	chr2:112829750-112864043	0.61	4.30	2.82	0.00	0.00
XLOC_031683	ZNF812	chr19:9689923-9700817	0.10	0.71	2.82	0.00	0.00
XLOC_001091	PCSK9	chr1:55039447-55064906	0.98	6.91	2.82	0.00	0.00
XLOC_056032	TNFRSF21	chr6:47231378-47309974	1.96	13.80	2.82	0.00	0.00
XLOC_006010	RP11-295G20.2	chr1:231519950-231528643	14.83	103.40	2.80	0.00	0.00
XLOC_024293	CARHSP1	chr16:8674564-8869028	13.25	92.24	2.80	0.00	0.00
XLOC_044964	WNT5A	chr3:55465714-55490539	2.04	14.07	2.79	0.00	0.00
XLOC_060058	SAMD9	chr7:93098363-93118023	2.11	14.55	2.79	0.00	0.00
XLOC_014068	OAS1	chr12:112906776-113017751	5.58	38.01	2.77	0.00	0.00
XLOC_060015	STEAP4	chr7:87934127-88306993	5.08	34.18	2.75	0.00	0.00
XLOC_017209	EPSTI1	chr13:42886202-42992271	1.13	7.60	2.75	0.00	0.00
XLOC_059320	STEAP1B	chr7:22418996-22665533	0.22	1.45	2.75	0.00	0.00
XLOC_032337	CNFN	chr19:42387018-42390287	248.25	1650.74	2.73	0.00	0.00
XLOC_030144	ANGPTL4	chr19:7958578-8489114	5.44	36.08	2.73	0.00	0.00
XLOC_030107	TNFSF9	chr19:6530998-6536793	0.41	2.73	2.72	0.00	0.00
XLOC_063464	FAM83A-AS1	chr8:123178863-123210471	0.13	0.86	2.71	0.00	0.00
XLOC_060269	NAMPT	chr7:106248119-106286326	30.99	200.22	2.69	0.00	0.00
XLOC_007382	INA	chr10:103277146-103290351	0.72	4.63	2.69	0.00	0.00

gene_id	gene	locus	NN	PP	log2(fold_change)	p_value	q_value
XLOC_007199	IFIT3	chr10:89213464-89414557	2.82	18.08	2.68	0.00	0.00
XLOC_052794	PROB1	chr5:139390591-139404088	0.49	3.10	2.68	0.00	0.00
XLOC_045299	ZBED2	chr3:111292718-111665750	0.61	3.85	2.66	0.00	0.00
XLOC_011147	SAA2, SAA2-SAA4, SAA4	chr11:18231348-18248643	0.34	2.13	2.65	0.00	0.00
XLOC_052835	FCHSD1	chr5:141636949-141651621	14.25	88.67	2.64	0.00	0.00
XLOC_028973	LIPG	chr18:49560698-49599685	1.53	9.52	2.63	0.00	0.00
XLOC_048819	HPSE	chr4:83292460-83335153	6.65	41.13	2.63	0.00	0.00
XLOC_055950	TREM2	chr6:41158505-41163186	0.13	0.83	2.62	0.00	0.00
XLOC_041546	APOL6	chr22:35648374-35668462	2.43	14.85	2.61	0.00	0.00
XLOC_032931	AC092580.4	chr2:7725568-7732348	0.37	2.26	2.61	0.00	0.01
XLOC_069890	HAUS7, TREX2	chrX:153432711-153509554	16.45	100.50	2.61	0.00	0.00
XLOC_003148	RP11-34P13.16	chr1:89217-181062	0.34	2.05	2.60	0.00	0.00
XLOC_022705	RP11-66B24.4	chr15:100849560-101086066	1.25	7.60	2.60	0.00	0.00
XLOC_011721	FOSL1	chr11:65891762-65900573	0.56	3.36	2.59	0.00	0.00
XLOC_051216	FABP6	chr5:160187366-160238735	0.17	1.01	2.57	0.00	0.00
XLOC_025547	XAF1	chr17:6755432-6776116	2.48	14.76	2.57	0.00	0.00
XLOC_059133	RP11-482G13.1	chr7:12703-36681	0.47	2.79	2.56	0.00	0.00
XLOC_058238	CD36	chr7:80312573-80682610	16.51	96.73	2.55	0.00	0.00
XLOC_028433	SOCS3	chr17:78355177-78360077	3.39	19.87	2.55	0.00	0.00
XLOC_024072	FAM157C	chr16:90102220-90222678	0.17	0.97	2.55	0.00	0.00
XLOC_046204	-	chr3:193824058-193837852	0.74	4.27	2.53	0.00	0.00
XLOC_035924	XDH	chr2:31334009-31429404	0.76	4.37	2.52	0.00	0.00
XLOC_041824	SYCE3	chr22:50551111-50565795	0.16	0.91	2.50	0.00	0.00
XLOC_030874	LIPE-AS1	chr19:42397127-42740569	6.32	35.74	2.50	0.00	0.00
XLOC_007202	IFIT1	chr10:89213464-89414557	3.17	17.92	2.50	0.00	0.00
XLOC_013489	IL23A	chr12:56334173-56340410	0.17	0.95	2.50	0.00	0.00
XLOC_055287	SERPINB1	chr6:2832331-2842006	8.74	49.24	2.49	0.00	0.00
XLOC_045413	PARP9	chr3:122527772-122575203	5.10	28.21	2.47	0.00	0.00
XLOC_002453	RGS1	chr1:192491723-192766340	0.67	3.73	2.47	0.00	0.00
XLOC_019155	RP11-203M5.8	chr14:20468953-20482076	1.76	9.71	2.46	0.00	0.00
XLOC_024802	NETO2	chr16:47077692-47144014	0.75	4.08	2.45	0.00	0.00
XLOC_049607	-	chr4:183794945-184023924	0.48	2.64	2.44	0.00	0.00
XLOC_022462	BCL2A1	chr15:79960888-79971446	0.59	3.21	2.44	0.00	0.00
XLOC_031846	UCA1	chr19:15827044-15838385	0.20	1.06	2.44	0.00	0.01
XLOC_002224	ATP1B1	chr1:169105696-169367967	22.91	123.66	2.43	0.00	0.00
XLOC_044392	FAM157A	chr3:198153286-198222513	0.22	1.21	2.43	0.00	0.00
XLOC_034535	DHRS9	chr2:168918130-169101210	1.98	10.58	2.42	0.00	0.00

gene_id	gene	locus	NN	PP	log2(fold_change)	p_value	q_value
XLOC_029099	SERPINB13	chr18:63586988-63604639	18.07	96.64	2.42	0.00	0.00
XLOC_065957	TRPM6	chr9:74722404-74888094	0.15	0.81	2.42	0.00	0.00
XLOC_026413	B4GALNT2	chr17:49095388-49177580	0.37	1.97	2.42	0.00	0.00
XLOC_038243	TGM3	chr20:2295952-2346939	45.73	243.53	2.41	0.00	0.00
XLOC_012594	-	chr11:129591492-129815147	0.14	0.73	2.41	0.00	0.00
XLOC_017820	PNP	chr14:20468953-20482076	10.68	55.43	2.38	0.00	0.00
XLOC_031032	FUT2	chr19:48695805-48708218	4.86	25.25	2.38	0.00	0.00
XLOC_032237	-	chr19:38990713-39117070	0.21	1.08	2.36	0.00	0.00
XLOC_061450	LYN	chr8:55879812-56014421	2.33	11.84	2.35	0.00	0.00
XLOC_027676	C17orf96	chr17:38671702-38729803	1.64	8.35	2.34	0.00	0.00
XLOC_025596	AC129492.6	chr17:8063935-8088016	1.55	7.85	2.34	0.00	0.00
XLOC_009909	RP11-783K16.5	chr11:64244463-64249494	6.10	30.96	2.34	0.00	0.00
XLOC_013631	LYZ	chr12:69348340-69354234	10.44	52.96	2.34	0.00	0.00
XLOC_050083	CARD6	chr5:40841183-40860175	1.08	5.49	2.34	0.00	0.00
XLOC_010750	TMEM45B	chr11:129815818-129895620	16.80	84.43	2.33	0.00	0.00
XLOC_005308	NME7	chr1:169105696-169367967	14.24	71.32	2.32	0.00	0.00
XLOC_019265	TGM1	chr14:24244271-24264432	39.41	197.10	2.32	0.00	0.00
XLOC_012408	MPZL2	chr11:118253402-118264536	27.87	138.14	2.31	0.00	0.00
XLOC_067066	-	chrX:7040872-7446415	0.19	0.92	2.31	0.00	0.00
XLOC_028693	RAB31	chr18:9708164-9862717	7.27	35.95	2.30	0.00	0.00
XLOC_009477	EHF	chr11:34620760-34666569	28.34	140.04	2.30	0.00	0.00
XLOC_023191	IL4R	chr16:27313386-27364778	7.77	38.19	2.30	0.00	0.00
XLOC_015023	GALNT6	chr12:51351246-51515763	5.14	25.22	2.30	0.00	0.00
XLOC_007392	SH3PXD2A-AS1	chr10:103593042-104029233	3.03	14.86	2.30	0.00	0.00
XLOC_056973	RAET1G	chr6:149724314-149923121	1.16	5.70	2.29	0.00	0.00
XLOC_040275	DSCR8,KCNJ15	chr21:38121450-38307448	1.74	8.48	2.28	0.00	0.00
XLOC_054667	FAM26F	chr6:116460738-116464944	0.69	3.34	2.28	0.00	0.00
XLOC_023249	RP11-455F5.6	chr16:30104395-30123506	0.16	0.75	2.27	0.00	0.00
XLOC_005213	SLAMF1	chr1:160608099-160647295	0.17	0.81	2.26	0.00	0.00
XLOC_064581	CTSL	chr9:87725518-87731393	8.60	41.25	2.26	0.00	0.00
XLOC_055958	TREM1	chr6:41267925-41286719	0.16	0.75	2.26	0.00	0.00
XLOC_038559	ID1	chr20:31605282-31606515	44.41	211.37	2.25	0.00	0.00
XLOC_033165	EFR3B	chr2:25042129-25159423	0.43	2.03	2.25	0.00	0.00
XLOC_035280	CXCR2	chr2:218125288-218137259	2.46	11.68	2.25	0.00	0.00
XLOC_015329	RP11-1143G9.4	chr12:69348340-69354234	9.63	45.76	2.25	0.00	0.00
XLOC_051785	-	chr5:34396437-34396748	0.66	3.14	2.24	0.00	0.01
XLOC_038504	CST7	chr20:24949201-24992979	1.00	4.74	2.24	0.00	0.00
XLOC_004388	GBP1	chr1:89051513-89065363	5.22	24.54	2.23	0.00	0.00

gene_id	gene	locus	NN	PP	log2(fold_change)	p_value	q_value
XLOC_053205	GPRIN1	chr5:176526342-176610133	0.96	4.52	2.23	0.00	0.00
XLOC_034143	IL36RN	chr2:113058637-113065382	40.92	191.99	2.23	0.00	0.00
XLOC_009484	RP11-350D17.3	chr11:34709269-34715086	0.16	0.75	2.23	0.00	0.01
XLOC_002081	SLAMF7	chr1:160739056-160755112	0.71	3.30	2.21	0.00	0.00
XLOC_011332	CHST1	chr11:45648876-45665622	0.21	0.96	2.21	0.00	0.00
XLOC_010820	IRF7	chr11:575704-616003	8.56	39.52	2.21	0.00	0.00
XLOC_031564	CTB-50L17.14, LRG1,PLIN5	chr19:4522440-4540474	7.53	34.70	2.21	0.00	0.00
XLOC_054382	TPBG	chr6:82363205-82401505	6.74	31.07	2.20	0.00	0.00
XLOC_032582	KLK10	chr19:50941230-51020175	31.37	143.71	2.20	0.00	0.00
XLOC_027730	CCR7	chr17:40552261-40565472	0.40	1.82	2.19	0.00	0.00
XLOC_028443	LGALS3BP	chr17:78971186-78980109	16.33	74.45	2.19	0.00	0.00
XLOC_061322	IDO1	chr8:39902274-40016391	0.29	1.30	2.19	0.00	0.00
XLOC_005121	SH2D2A	chr1:156806242-156881850	0.20	0.89	2.19	0.00	0.00
XLOC_014069	OAS3	chr12:112906776-113017751	5.48	24.92	2.19	0.00	0.00
XLOC_009185	OR56B1, TRIM22	chr11:5663234-5938619	5.91	26.84	2.18	0.00	0.00
XLOC_001895	LINC01527	chr1:152897815-152955627	0.79	3.57	2.18	0.00	0.00
XLOC_002102	PCP4L1	chr1:161258726-161289057	3.15	14.25	2.18	0.00	0.00
XLOC_019652	RP11-1112J20.2	chr14:63122614-63183526	0.36	1.62	2.18	0.00	0.00
XLOC_038841	LINC01272	chr20:50267485-50279795	0.21	0.96	2.18	0.00	0.00
XLOC_007760	RP11-116G8.5	chr10:5514243-5531184	271.60	1225.76	2.17	0.00	0.00
XLOC_001618	CD2	chr1:116754384-116769230	0.57	2.58	2.17	0.00	0.00
XLOC_028857	DSG3	chr18:31447556-31486660	36.66	165.01	2.17	0.00	0.00
XLOC_029463	ANKRD29	chr18:23598925-23663203	0.83	3.71	2.17	0.00	0.00
XLOC_051143	GPX3	chr5:151020437-151028993	0.26	1.16	2.16	0.00	0.01
XLOC_019059	ASPG,KIF26A	chr14:104085678-104181093	9.56	42.71	2.16	0.00	0.00
XLOC_042586	IRAK2	chr3:10164718-10243888	1.52	6.75	2.15	0.00	0.00
XLOC_006308	CALML3	chr10:5514243-5531184	54.53	241.74	2.15	0.00	0.00
XLOC_014615	PLBD1	chr12:14503595-14757963	21.89	97.01	2.15	0.00	0.00
XLOC_056048	CRISP3	chr6:49727380-49744437	0.49	2.18	2.15	0.00	0.00
XLOC_023716	HAS3	chr16:69105563-69408571	2.90	12.86	2.15	0.00	0.00
XLOC_041082	USP18	chr22:18149723-18177397	0.98	4.32	2.15	0.00	0.00
XLOC_031851	CYP4F11	chr19:15907826-15934881	0.38	1.68	2.14	0.00	0.00
XLOC_065200	PAEP	chr9:135561755-135566955	0.19	0.85	2.14	0.00	0.00
XLOC_036556	IGKV3-20	chr2:89142573-89143160	0.61	2.66	2.13	0.00	0.01
XLOC_025595	RP11-599B13.9	chr17:8063935-8088016	0.29	1.26	2.13	0.00	0.00
XLOC_055290	SERPINB9	chr6:2887265-2903325	1.01	4.41	2.13	0.00	0.00
XLOC_025665	RP11-214O1.2	chr17:14301082-14349404	0.34	1.50	2.13	0.00	0.01

gene_id	gene	locus	NN	PP	log2(fold_change)	p_value	q_value
XLOC_000690	TRIM62	chr1:33124076-33301015	0.19	0.83	2.13	0.00	0.00
XLOC_050266	CCNB1	chr5:69167009-69178245	5.47	23.88	2.13	0.00	0.00
XLOC_058869	TRBV20-1	chr7:142626648-142627399	0.38	1.66	2.12	0.00	0.01
XLOC_030599	CCNE1	chr19:29811779-29824405	1.64	7.15	2.12	0.00	0.00
XLOC_012099	CTSC	chr11:88293553-88337791	21.04	91.47	2.12	0.00	0.00
XLOC_000045	SCNN1D	chr1:1274716-1292188	1.24	5.33	2.11	0.00	0.00
XLOC_015214	AGAP2	chr12:57723760-57761067	0.88	3.80	2.11	0.00	0.00
XLOC_022442	CHRNA3	chr15:78565490-78621295	0.31	1.35	2.10	0.00	0.00
XLOC_003961	PDZK1IP1	chr1:47183435-47191044	52.78	226.21	2.10	0.00	0.00
XLOC_008815	AFAP1L2	chr10:114239222-114405186	4.92	21.05	2.10	0.00	0.00
XLOC_027794	KRT42P	chr17:41626326-41640199	0.61	2.60	2.10	0.00	0.00
XLOC_008334	LRRC20	chr10:70298682-70382688	1.42	6.04	2.09	0.00	0.00
XLOC_064114	-	chr9:38078383-38094674	0.27	1.14	2.08	0.00	0.00
XLOC_024743	BCAP31P1, RP11-812E19.14, RP11-812E19.3	chr16:33965578-33991740	0.51	2.17	2.08	0.00	0.00
XLOC_001894	IVL	chr1:152897815-152955627	43.72	184.41	2.08	0.00	0.00
XLOC_004390	GBP4	chr1:89181147-89198945	1.04	4.39	2.07	0.00	0.00
XLOC_047041	AREG	chr4:74445133-74455653	1.21	5.09	2.07	0.00	0.00
XLOC_032964	RRM2	chr2:10120733-10131419	5.52	23.15	2.07	0.00	0.00
XLOC_011266	ELF5	chr11:34478670-34525096	0.79	3.32	2.07	0.00	0.00
XLOC_004029	LRP8	chr1:53226891-53328070	1.32	5.51	2.06	0.00	0.00
XLOC_020730	CTD-2382E5.2	chr15:41972762-42051190	0.67	2.79	2.06	0.00	0.00
XLOC_041610	APOBEC3A, APOBEC3B	chr22:38952740-38998209	1.06	4.41	2.06	0.00	0.00
XLOC_003601	CD164L2	chr1:27379173-27383380	1.94	8.01	2.05	0.00	0.00
XLOC_040840	AP000692.10	chr21:36319791-36419015	0.32	1.32	2.05	0.00	0.01
XLOC_035133	CTLA4	chr2:203853905-203874119	0.20	0.81	2.04	0.00	0.00
XLOC_057174	SOD2	chr6:159669056-159789749	52.37	215.46	2.04	0.00	0.00
XLOC_021396	ISG20	chr15:88636152-88656483	2.31	9.50	2.04	0.00	0.00
XLOC_023342	BCAP31P2, RP11-989E6.3	chr16:32869793-32890918	0.19	0.78	2.04	0.00	0.00
XLOC_010806	IFITM3	chr11:318639-330122	65.67	269.13	2.04	0.00	0.00
XLOC_016923	GJA3	chr13:20138254-20161478	0.21	0.85	2.03	0.00	0.00
XLOC_064036	PRSS3	chr9:33750465-34049181	24.54	100.04	2.03	0.00	0.00
XLOC_039765	FAM65C	chr20:50510320-50691538	1.26	5.14	2.03	0.00	0.00
XLOC_003997	TTC39A	chr1:51285852-51350193	6.90	28.06	2.02	0.00	0.00
XLOC_055574	HIST1H1A	chr6:26017084-26017732	5.85	23.80	2.02	0.00	0.00
XLOC_001009	TTC39A-AS1	chr1:51285852-51350193	0.25	1.00	2.02	0.00	0.00
XLOC_026028	CCL2	chr17:34255217-34257203	6.02	24.34	2.01	0.00	0.00
XLOC_021933	PLA2G4E	chr15:41972762-42051190	21.03	84.75	2.01	0.00	0.00

gene_id	gene	locus	NN	PP	log2(fold_change)	p_value	q_value
XLOC_017623	RP11-12G12.7	chr13:100083705-100589528	1.21	4.89	2.01	0.00	0.00
XLOC_042233	APOL2	chr22:36225910-36239954	4.04	16.22	2.01	0.00	0.00
XLOC_052648	FBN2	chr5:128257908-129033882	0.31	1.24	2.00	0.00	0.00
XLOC_047389	PDE5A	chr4:119454700-119628991	0.75	0.19	-2.00	0.00	0.01
XLOC_025701	LRRC75A	chr17:16438821-16492153	1.88	0.47	-2.01	0.00	0.00
XLOC_014644	NDFIP1P1	chr12:18080648-18773385	0.81	0.20	-2.01	0.00	0.00
XLOC_004948	FLG2	chr1:152122533-152445456	756.93	188.44	-2.01	0.00	0.01
XLOC_043418	SLC9C1	chr3:112086334-112294258	2.89	0.72	-2.01	0.00	0.00
XLOC_065683	CNTNAP3	chr9:39072048-39288443	15.81	3.92	-2.01	0.00	0.00
XLOC_051341	FAM153B	chr5:176049677-176132258	5.85	1.45	-2.01	0.00	0.00
XLOC_032430	AC007193.6	chr19:46078815-46250270	2.01	0.50	-2.02	0.00	0.00
XLOC_023746	IL34	chr16:70579521-70686066	51.50	12.69	-2.02	0.00	0.00
XLOC_031328	ZNF471	chr19:56507842-56530532	1.42	0.35	-2.03	0.00	0.00
XLOC_020749	MAP1A	chr15:43510957-43532330	2.61	0.64	-2.03	0.00	0.00
XLOC_001901	RP1-140J1.1	chr1:153174517-153215617	1.10	0.27	-2.03	0.00	0.00
XLOC_069579	TMEM255A	chrX:120258649-120315775	2.20	0.54	-2.03	0.00	0.00
XLOC_029332	LINC01254	chr18:10405132-10425411	0.73	0.18	-2.03	0.00	0.00
XLOC_048746	SOWAHB	chr4:76893789-76898211	2.04	0.50	-2.03	0.00	0.00
XLOC_058456	LRRC17	chr7:102748970-103149560	1.26	0.31	-2.03	0.00	0.00
XLOC_017675	-	chr13:105570154-105599548	2.77	0.68	-2.03	0.00	0.00
XLOC_004099	CYP2J2	chr1:59893307-59926790	10.28	2.51	-2.03	0.00	0.00
XLOC_046967	TMPRSS11E	chr4:68447413-68501058	3.24	0.79	-2.04	0.00	0.00
XLOC_023880	GAN	chr16:81314934-81396605	116.59	28.35	-2.04	0.00	0.00
XLOC_046241	APOD	chr3:195568701-195584205	33.27	8.06	-2.05	0.00	0.00
XLOC_014670	SOX5	chr12:23528854-23951197	7.94	1.92	-2.05	0.00	0.00
XLOC_062488	RPS3AP34	chr8:12448012-12708364	1.92	0.47	-2.05	0.00	0.00
XLOC_068494	BGN	chrX:153432711-153509554	23.58	5.68	-2.05	0.00	0.00
XLOC_054669	FAM26E	chr6:116492296-116558868	1.17	0.28	-2.06	0.00	0.00
XLOC_009612	FAM180B	chr11:47586692-47589194	3.78	0.90	-2.06	0.00	0.00
XLOC_012221	MMP7	chr11:102520507-102530753	5.53	1.32	-2.07	0.00	0.00
XLOC_064163	RP11-290L7.5	chr9:39072048-39288443	1.25	0.30	-2.07	0.00	0.00
XLOC_007506	SLC18A2	chr10:117239599-117375467	1.45	0.34	-2.07	0.00	0.00
XLOC_026512	ANKFN1	chr17:56110957-56516998	2.18	0.52	-2.07	0.00	0.00
XLOC_022281	ITGA11	chr15:68267791-68432314	2.85	0.68	-2.07	0.00	0.00
XLOC_045035	MAGI1-AS1	chr3:65353016-66038834	1.14	0.27	-2.07	0.00	0.00
XLOC_018441	PELI2	chr14:56117315-56310761	6.51	1.55	-2.08	0.00	0.00
XLOC_032431	AC007193.6	chr19:46078815-46250270	2.41	0.57	-2.08	0.00	0.01
XLOC_009058	CRACR2B	chr11:818901-842545	2.90	0.69	-2.08	0.00	0.00

gene_id	gene	locus	NN	PP	log2(fold_change)	p_value	q_value
XLOC_021257	RP11-307C19.2	chr15:77568969-77608888	1.85	0.44	-2.08	0.00	0.00
XLOC_056429	TBX18	chr6:84687350-84764592	2.96	0.70	-2.08	0.00	0.00
XLOC_004713	HMGCS2	chr1:119747900-119769001	2.66	0.63	-2.09	0.00	0.00
XLOC_064270	-	chr9:61330412-61380478	1.66	0.39	-2.09	0.00	0.00
XLOC_045445	ENPP7P4	chr3:125826844-125916384	0.79	0.19	-2.09	0.00	0.01
XLOC_043421	SLC9C1	chr3:112086334-112294258	1.06	0.25	-2.09	0.00	0.00
XLOC_008681	LOXL4	chr10:98134491-98268250	2.37	0.55	-2.09	0.00	0.00
XLOC_055346	RP1-80N2.2	chr6:6691955-6745253	3.27	0.77	-2.10	0.00	0.00
XLOC_004139	LINC01359	chr1:64972224-65002489	0.93	0.22	-2.10	0.00	0.00
XLOC_045013	CADPS	chr3:62398294-62875389	1.52	0.35	-2.10	0.00	0.00
XLOC_019092	AL928654.7, C14orf80,CRIP1	chr14:105486316-105499575	291.24	67.76	-2.10	0.00	0.00
XLOC_056075	GSTA3, GSTA5	chr6:52831654-52909717	7.49	1.74	-2.11	0.00	0.00
XLOC_051626	FAM134B	chr5:16448159-16629969	10.53	2.45	-2.11	0.00	0.00
XLOC_023151	CHP2	chr16:23753688-23759057	103.25	23.92	-2.11	0.00	0.00
XLOC_008827	GFRA1	chr10:116056924-116273998	7.96	1.84	-2.11	0.00	0.00
XLOC_060506	PLXNA4	chr7:132123283-132648688	2.82	0.65	-2.11	0.00	0.00
XLOC_056603	METTL24	chr6:110180140-110358474	0.85	0.20	-2.11	0.00	0.00
XLOC_018703	NRXN3	chr14:78170372-79868290	1.09	0.25	-2.11	0.00	0.00
XLOC_035769	OSR1	chr2:19351484-19364109	4.32	1.00	-2.11	0.00	0.00
XLOC_023099	ACSM3	chr16:20610242-20925006	4.19	0.97	-2.11	0.00	0.00
XLOC_036009	SLC8A1	chr2:39786452-40611053	17.05	3.94	-2.11	0.00	0.00
XLOC_046174	-	chr3:189415689-189903858	1.88	0.43	-2.11	0.00	0.00
XLOC_009409	BDNF-AS	chr11:27506837-27877648	2.31	0.53	-2.12	0.00	0.00
XLOC_029960	PALM	chr19:708938-748332	11.44	2.63	-2.12	0.00	0.00
XLOC_054768	FABP7	chr6:122778494-122784074	37.94	8.70	-2.12	0.00	0.00
XLOC_050904	CTC-321K16.4	chr5:135634962-135828910	1.61	0.37	-2.13	0.00	0.00
XLOC_051107	ADRB2	chr5:148451031-149063163	5.97	1.36	-2.13	0.00	0.00
XLOC_024149	HS3ST6	chr16:1901752-1918440	77.88	17.71	-2.14	0.00	0.00
XLOC_049113	NDNF	chr4:121030692-121080476	1.43	0.32	-2.14	0.00	0.00
XLOC_031160	ZNF528-AS1	chr19:52369843-52418709	1.23	0.28	-2.14	0.00	0.00
XLOC_064234	RP11-24B13.2	chr9:41889198-42129510	0.90	0.20	-2.14	0.00	0.00
XLOC_045033	MAGI1-AS1	chr3:65353016-66038834	1.25	0.28	-2.14	0.00	0.01
XLOC_068731	RAI2	chrX:17800042-17861337	2.99	0.68	-2.14	0.00	0.00
XLOC_060550	ATP6V0A4, SVOPL	chr7:138593969-138981318	2.71	0.62	-2.14	0.00	0.00
XLOC_061143	PEBP4	chr8:22678706-23083619	1.24	0.28	-2.14	0.00	0.00
XLOC_012835	RP11-113C12.4	chr12:8254526-8396803	0.74	0.17	-2.14	0.00	0.00
XLOC_053582	ID4	chr6:19534535-19847453	124.74	28.16	-2.15	0.00	0.00

gene_id	gene	locus	NN	PP	log2(fold_change)	p_value	q_value
XLOC_019922	PTPN21	chr14:88384923-88555056	45.03	10.16	-2.15	0.00	0.00
XLOC_042143	LIF	chr22:30239193-30257981	2.60	0.59	-2.15	0.00	0.00
XLOC_037655	NAB1	chr2:190606685-190708716	1.05	0.23	-2.16	0.00	0.00
XLOC_065598	CCL27, RP11-195F19.5	chr9:34638132-34681298	157.69	35.35	-2.16	0.00	0.00
XLOC_024760	FRG2HP	chr16:35315906-35336932	9.90	2.22	-2.16	0.00	0.00
XLOC_045666	PCOLCE2	chr3:142815921-142889203	6.70	1.50	-2.16	0.00	0.00
XLOC_037841	ACADL	chr2:210171517-210230383	2.04	0.45	-2.17	0.00	0.00
XLOC_069340	NAP1L3	chrX:93670929-93673568	1.15	0.25	-2.17	0.00	0.00
XLOC_024900	CES1	chr16:55802846-55833337	7.44	1.65	-2.17	0.00	0.00
XLOC_029968	CFD	chr19:859448-863630	249.67	55.42	-2.17	0.00	0.00
XLOC_002414	PRG4	chr1:186296278-186421764	1.45	0.32	-2.17	0.00	0.00
XLOC_013766	ACSS3	chr12:80792519-81759553	2.45	0.54	-2.17	0.00	0.00
XLOC_046182	-	chr3:190292901-190297567	0.82	0.18	-2.18	0.00	0.00
XLOC_004438	BCAR3	chr1:93448066-93848939	9.26	2.03	-2.19	0.00	0.00
XLOC_044508	TIMP4	chr3:12004401-12191705	1.11	0.24	-2.19	0.00	0.00
XLOC_002334	SOAT1	chr1:179273165-179358680	16.09	3.51	-2.20	0.00	0.00
XLOC_046183	CLDN1	chr3:190305700-190412237	260.82	56.67	-2.20	0.00	0.00
XLOC_001519	GSTM5	chr1:109712254-109775428	2.95	0.64	-2.21	0.00	0.00
XLOC_062185	PSCA	chr8:142641560-142682724	3.69	0.80	-2.21	0.00	0.00
XLOC_054924	ARFGEF3	chr6:138161784-138344663	1.15	0.25	-2.21	0.00	0.00
XLOC_004023	RP11-334A14.5	chr1:53062051-53142632	6.25	1.35	-2.21	0.00	0.00
XLOC_006906	LINC01515	chr10:65570284-65820595	14.52	3.11	-2.22	0.00	0.00
XLOC_047420	LINC01091, RP11-381N20.1	chr4:123490267-123934766	2.90	0.62	-2.22	0.00	0.00
XLOC_066091	OMD	chr9:92297357-92674943	5.03	1.07	-2.23	0.00	0.00
XLOC_023543	IRX6	chr16:55258674-55346844	4.26	0.91	-2.23	0.00	0.00
XLOC_015794	TBX5	chr12:114353905-114412961	0.77	0.16	-2.23	0.00	0.00
XLOC_052832	AC005618.8	chr5:141330570-141512981	0.87	0.19	-2.23	0.00	0.00
XLOC_002678	RP11-372M18.2	chr1:209367661-209389072	1.47	0.31	-2.23	0.00	0.00
XLOC_038953	APCDD1L-AS1	chr20:58515378-58619888	1.40	0.30	-2.23	0.00	0.00
XLOC_028467	NPTX1	chr17:80466877-80477843	2.63	0.56	-2.24	0.00	0.00
XLOC_004453	F3	chr1:94529224-94562515	45.86	9.68	-2.24	0.00	0.00
XLOC_025672	RP11-849N15.1, RP11-849N15.3	chr17:15229776-15265707	1.05	0.22	-2.25	0.00	0.00
XLOC_046228	-	chr3:194981415-194999067	0.93	0.20	-2.25	0.00	0.00
XLOC_015044	KRT85	chr12:52360005-52367481	3.28	0.69	-2.25	0.00	0.00
XLOC_025563	SLC2A4	chr17:7281666-7294615	1.08	0.23	-2.26	0.00	0.00
XLOC_031301	SSC5D	chr19:55488403-55519117	5.60	1.17	-2.26	0.00	0.00
XLOC_002581	PRELP	chr1:203475782-203491643	5.02	1.05	-2.26	0.00	0.00

gene_id	gene	locus	NN	PP	log2(fold_change)	p_value	q_value
XLOC_022373	CYP1A1	chr15:74719437-74725679	1.55	0.32	-2.26	0.00	0.00
XLOC_044995	ACOX2	chr3:58505135-58537360	3.66	0.76	-2.26	0.00	0.00
XLOC_044705	ITGA9	chr3:37441319-37861780	1.39	0.29	-2.27	0.00	0.00
XLOC_068820	TMEM47	chrX:34627063-34657288	4.92	1.02	-2.27	0.00	0.00
XLOC_032643	ZNF415	chr19:53062150-53193386	2.11	0.44	-2.27	0.00	0.00
XLOC_008717	PDZD7	chr10:100996598-101031157	1.05	0.22	-2.28	0.00	0.00
XLOC_020975	AQP9	chr15:57953423-58570035	22.30	4.60	-2.28	0.00	0.00
XLOC_047922	HAND2-AS1	chr4:173524968-173591324	1.20	0.25	-2.28	0.00	0.00
XLOC_043014	SEMA3B	chr3:50267392-50277546	5.60	1.15	-2.28	0.00	0.00
XLOC_013355	OR7E47P	chr12:52069245-52118207	0.82	0.17	-2.29	0.00	0.00
XLOC_042527	CNTN4	chr3:2098751-3126613	1.38	0.28	-2.29	0.00	0.00
XLOC_055561	-	chr6:25077351-25218560	0.73	0.15	-2.29	0.00	0.01
XLOC_059306	SP8	chr7:20615206-20787054	6.18	1.26	-2.29	0.00	0.00
XLOC_041618	PDGFB	chr22:39215719-39249159	0.76	0.16	-2.29	0.00	0.00
XLOC_014505	MFAP5	chr12:8634366-8662888	23.23	4.75	-2.29	0.00	0.00
XLOC_028518	NOTUM	chr17:81952506-81961840	0.93	0.19	-2.29	0.00	0.00
XLOC_043420	SLC9C1	chr3:112086334-112294258	1.18	0.24	-2.29	0.00	0.00
XLOC_037883	IGFBP5	chr2:216671971-216994079	80.04	16.25	-2.30	0.00	0.00
XLOC_010859	PRR33	chr11:1852969-1917678	2.45	0.50	-2.30	0.00	0.00
XLOC_061040	-	chr8:12448012-12708364	1.66	0.34	-2.30	0.00	0.00
XLOC_032570	KLK1	chr19:50818906-50823787	12.92	2.61	-2.31	0.00	0.00
XLOC_065593	CNTFR	chr9:34551346-34591830	1.55	0.31	-2.31	0.00	0.00
XLOC_010707	DDX25	chr11:125883613-125939111	1.06	0.21	-2.31	0.00	0.00
XLOC_003226	RP4-740C4.7	chr1:2321173-2391732	1.03	0.21	-2.32	0.00	0.00
XLOC_045452	KLF15	chr3:126301346-126357442	1.26	0.25	-2.32	0.00	0.00
XLOC_017972	DHRS2	chr14:23630114-23645639	0.91	0.18	-2.32	0.00	0.00
XLOC_015032	AC078864.1, RP1-288H2.2	chr12:52069245-52118207	1.34	0.27	-2.33	0.00	0.00
XLOC_047181	RP11-115D19.1	chr4:89551355-89841978	0.95	0.19	-2.33	0.00	0.00
XLOC_069495	CHRDL1	chrX:110671940-110796414	10.02	2.00	-2.33	0.00	0.00
XLOC_047675	GLRB	chr4:157076056-157172090	1.09	0.22	-2.33	0.00	0.00
XLOC_029074	CDH20	chr18:61333581-61555863	0.76	0.15	-2.33	0.00	0.00
XLOC_021273	CRABP1	chr15:78339207-78348230	6.60	1.30	-2.34	0.00	0.00
XLOC_012472	CLMP	chr11:123068355-123228277	8.55	1.68	-2.35	0.00	0.00
XLOC_043422	RP11-757F18.3	chr3:112086334-112294258	0.92	0.18	-2.36	0.00	0.00
XLOC_062182	ADGRB1	chr8:142444494-142545009	2.68	0.52	-2.36	0.00	0.00
XLOC_055560	AL590084.1	chr6:25077351-25218560	0.74	0.14	-2.37	0.00	0.00
XLOC_064649	WNK2	chr9:93184134-93346414	8.33	1.62	-2.37	0.00	0.00
XLOC_052746	CTC-321K16.4	chr5:135634962-135828910	1.42	0.27	-2.37	0.00	0.00

gene_id	gene	locus	NN	PP	log2(fold_change)	p_value	q_value
XLOC_065099	RP11-344B5.2	chr9:129282407-129288674	2.95	0.57	-2.37	0.00	0.00
XLOC_017121	POSTN	chr13:37562429-37598897	90.85	17.56	-2.37	0.00	0.00
XLOC_065680	ANKRD18A	chr9:38568683-38625132	1.71	0.33	-2.39	0.00	0.00
XLOC_067928	-	chrX:93434920-93438590	0.80	0.15	-2.39	0.00	0.00
XLOC_053342	FOXCUT	chr6:1604213-1607599	1.22	0.23	-2.39	0.00	0.00
XLOC_004977	RP1-140J1.1	chr1:153174517-153215617	2.11	0.40	-2.39	0.00	0.00
XLOC_008517	LRIT2	chr10:84194387-84225589	1.41	0.27	-2.39	0.00	0.00
XLOC_033319	AC007317.1	chr2:40716945-40770668	3.79	0.72	-2.39	0.00	0.00
XLOC_027783	KRT35	chr17:41399001-41481140	1.04	0.20	-2.40	0.00	0.01
XLOC_032614	HAS1	chr19:51685362-51724137	1.32	0.25	-2.40	0.00	0.00
XLOC_046379	RP11-20I20.4	chr4:1166931-1209393	1.34	0.25	-2.40	0.00	0.00
XLOC_013063	RP11-666F17.1	chr12:26117866-26833260	1.90	0.36	-2.41	0.00	0.00
XLOC_007733	-	chr10:3432492-3467563	2.16	0.41	-2.41	0.00	0.00
XLOC_007792	-	chr10:8569756-8595407	1.29	0.24	-2.41	0.00	0.00
XLOC_043419	SLC9C1	chr3:112086334-112294258	1.82	0.34	-2.42	0.00	0.01
XLOC_028322	AC005152.3, SOX9-AS1	chr17:72021850-72237203	2.00	0.37	-2.42	0.00	0.00
XLOC_022586	PLIN1	chr15:89664364-89690783	2.10	0.39	-2.42	0.00	0.00
XLOC_025388	SLC22A31	chr16:89195760-89201664	2.16	0.40	-2.43	0.00	0.00
XLOC_056844	NHEG1	chr6:136965395-136999690	0.91	0.17	-2.43	0.00	0.00
XLOC_066676	ABO	chr9:133233290-133275214	1.15	0.21	-2.43	0.00	0.00
XLOC_066090	OGN	chr9:92297357-92674943	3.78	0.70	-2.44	0.00	0.00
XLOC_042463	MLC1	chr22:50059209-50085902	1.11	0.20	-2.44	0.00	0.00
XLOC_045034	MAGI1-AS1	chr3:65353016-66038834	1.19	0.22	-2.44	0.00	0.00
XLOC_013647	LGR5	chr12:71124995-71586310	3.35	0.62	-2.44	0.00	0.00
XLOC_042044	GSTT2B	chr22:23957256-23984094	4.08	0.75	-2.45	0.00	0.00
XLOC_027788	KRT19	chr17:41523614-41537123	6.66	1.22	-2.45	0.00	0.00
XLOC_013090	FAR2	chr12:29149102-29381263	17.36	3.17	-2.45	0.00	0.00
XLOC_064424	FAM189A2	chr9:69324571-69393795	4.80	0.88	-2.45	0.00	0.00
XLOC_066123	FBP1	chr9:94603081-94640249	3.47	0.63	-2.46	0.00	0.00
XLOC_068315	FHL1	chrX:136146701-136211475	32.88	5.96	-2.46	0.00	0.00
XLOC_035245	AC007563.5	chr2:216671971-216994079	17.61	3.19	-2.46	0.00	0.00
XLOC_048498	CNGA1	chr4:47593792-48040173	2.61	0.47	-2.47	0.00	0.00
XLOC_030553	ZNF730	chr19:23075200-23148112	1.09	0.20	-2.47	0.00	0.00
XLOC_020424	RP11-701H24.3	chr15:24823636-25439099	0.80	0.14	-2.49	0.00	0.00
XLOC_059988	SEMA3E	chr7:83362376-83649156	1.52	0.27	-2.50	0.00	0.00
XLOC_016723	CLDN10	chr13:95433603-95644703	1.69	0.30	-2.51	0.00	0.00
XLOC_045449	ALDH1L1	chr3:126103561-126210169	0.81	0.14	-2.51	0.00	0.00
XLOC_059995	SEMA3D	chr7:84995128-85187172	2.86	0.50	-2.51	0.00	0.00

gene_id	gene	locus	NN	PP	log2(fold_change)	p_value	q_value
XLOC_022182	RP11-39M21.1	chr15:60479177-61229319	1.20	0.21	-2.51	0.00	0.01
XLOC_055546	CMAHP	chr6:25077351-25218560	17.81	3.10	-2.52	0.00	0.00
XLOC_048806	SCD5	chr4:82629538-82798982	7.96	1.38	-2.53	0.00	0.00
XLOC_015071	KRT4	chr12:52806548-52820393	0.72	0.13	-2.53	0.00	0.00
XLOC_032552	MYH14	chr19:50168089-50310545	1.18	0.20	-2.53	0.00	0.00
XLOC_006421	OLAH	chr10:15032226-15073853	0.87	0.15	-2.54	0.00	0.00
XLOC_065591	ENHO	chr9:34521039-34524241	1.37	0.23	-2.54	0.00	0.01
XLOC_030628	RGS9BP	chr19:32596906-32681760	1.51	0.26	-2.54	0.00	0.00
XLOC_015072	KRT79	chr12:52821256-52836899	60.61	10.40	-2.54	0.00	0.00
XLOC_061041	-	chr8:12448012-12708364	1.87	0.32	-2.55	0.00	0.00
XLOC_014673	-	chr12:24055859-24056661	0.94	0.16	-2.56	0.00	0.00
XLOC_057460	SCIN	chr7:12570124-12660179	5.22	0.89	-2.56	0.00	0.00
XLOC_061035	RP11-419I17.1	chr8:12448012-12708364	0.75	0.13	-2.56	0.00	0.00
XLOC_013360	KRT7	chr12:52232519-52309163	6.00	1.02	-2.56	0.00	0.00
XLOC_007149	SNCG	chr10:86935539-87024732	8.19	1.39	-2.56	0.00	0.00
XLOC_069169	P2RY4	chrX:70250925-70260217	1.45	0.25	-2.56	0.00	0.00
XLOC_065682	-	chr9:39068308-39070583	0.79	0.13	-2.57	0.00	0.00
XLOC_018838	C14orf132	chr14:96039303-96098023	17.21	2.90	-2.57	0.00	0.00
XLOC_061865	KB-1107E3.1	chr8:101686542-102124907	1.20	0.20	-2.58	0.00	0.00
XLOC_067232	CNKSR2	chrX:21374192-21658330	0.92	0.15	-2.58	0.00	0.00
XLOC_051267	GABRP	chr5:170747046-170814047	1.82	0.30	-2.58	0.00	0.00
XLOC_010775	NTM	chr11:131370477-132336978	4.62	0.77	-2.58	0.00	0.00
XLOC_020425	PWAR6	chr15:24823636-25439099	0.78	0.13	-2.61	0.00	0.00
XLOC_043738	CLSTN2	chr3:139935184-140577731	1.55	0.25	-2.61	0.00	0.00
XLOC_046184	CLDN16	chr3:190305700-190412237	2.24	0.37	-2.62	0.00	0.00
XLOC_014681	-	chr12:24191516-24562741	1.47	0.24	-2.62	0.00	0.00
XLOC_011275	SLC1A2	chr11:35251205-35421002	1.09	0.18	-2.62	0.00	0.00
XLOC_033633	ATP6V1B1, VAX2	chr2:70900589-71083371	2.93	0.48	-2.63	0.00	0.00
XLOC_048112	RP11-1263C18.1	chr4:572425-576119	4.20	0.68	-2.63	0.00	0.00
XLOC_048948	ADH6	chr4:99088856-99321571	1.46	0.23	-2.63	0.00	0.00
XLOC_014674	-	chr12:24057011-24057985	1.01	0.16	-2.63	0.00	0.00
XLOC_014676	-	chr12:24072750-24086530	1.05	0.17	-2.64	0.00	0.00
XLOC_014672	-	chr12:24007792-24055495	0.96	0.15	-2.65	0.00	0.00
XLOC_033227	FAM179A	chr2:28956610-29074261	1.09	0.17	-2.65	0.00	0.00
XLOC_052277	EDIL3	chr5:83940386-84490765	10.99	1.74	-2.66	0.00	0.00
XLOC_046594	C1QTNF7	chr4:15002443-15446166	3.50	0.55	-2.66	0.00	0.00
XLOC_051470	ZDHHC11B	chr5:612271-784746	3.60	0.57	-2.66	0.00	0.00
XLOC_065925	MAMDC2-AS1	chr9:69996082-70356817	1.15	0.18	-2.67	0.00	0.00

gene_id	gene	locus	NN	PP	log2(fold_change)	p_value	q_value
XLOC_014675	-	chr12:24059440-24071649	1.06	0.17	-2.67	0.00	0.00
XLOC_063444	SNTB1	chr8:120380760-120813359	16.18	2.53	-2.68	0.00	0.00
XLOC_057963	ZNF727	chr7:64045438-64086611	1.25	0.19	-2.69	0.00	0.00
XLOC_002861	C1orf95	chr1:226548781-226609290	1.80	0.28	-2.70	0.00	0.00
XLOC_019283	NOVA1	chr14:26443048-26597981	1.64	0.25	-2.70	0.00	0.00
XLOC_058108	RP11-731K22.1	chr7:73916739-74000066	2.73	0.42	-2.72	0.00	0.00
XLOC_002611	CNTN2	chr1:205043196-205078499	0.78	0.12	-2.72	0.00	0.00
XLOC_038766	WFDC2	chr20:45469705-45481532	2.57	0.39	-2.72	0.00	0.00
XLOC_042253	TMPRSS6	chr22:37065014-37109759	1.61	0.24	-2.72	0.00	0.00
XLOC_053229	FAM153A	chr5:177682293-177794396	1.28	0.19	-2.72	0.00	0.00
XLOC_031327	ZNF667-AS1	chr19:56439138-56500666	5.98	0.90	-2.73	0.00	0.00
XLOC_055133	FNDC1	chr6:159165898-159272353	0.85	0.13	-2.73	0.00	0.00
XLOC_005164	CADM3-AS1	chr1:159164658-159207973	5.92	0.89	-2.74	0.00	0.00
XLOC_055140	PNLDC1	chr6:159800116-159821123	2.00	0.30	-2.74	0.00	0.00
XLOC_014088	TBX5-AS1	chr12:114353905-114412961	1.27	0.19	-2.75	0.00	0.00
XLOC_061707	CA3	chr8:85319016-85481493	0.99	0.15	-2.75	0.00	0.00
XLOC_014679	-	chr12:24187382-24191047	1.67	0.25	-2.75	0.00	0.00
XLOC_032762	ZNF667	chr19:56439138-56500666	1.13	0.17	-2.76	0.00	0.00
XLOC_026131	PPP1R1B	chr17:39626739-39636638	4.46	0.66	-2.76	0.00	0.00
XLOC_014677	-	chr12:24086914-24180291	1.23	0.18	-2.76	0.00	0.00
XLOC_023868	LINC01229	chr16:79645561-79827150	2.57	0.38	-2.77	0.00	0.00
XLOC_011276	PAMR1	chr11:35431822-35530300	14.85	2.17	-2.78	0.00	0.00
XLOC_021234	ODF3L1	chr15:75723600-75763321	1.11	0.16	-2.78	0.00	0.00
XLOC_012215	ANGPTL5	chr11:101890437-102001255	3.52	0.51	-2.78	0.00	0.00
XLOC_045676	-	chr3:144374342-144379684	3.85	0.56	-2.78	0.00	0.00
XLOC_043144	ADAMTS9-AS2	chr3:64515653-65027017	1.46	0.21	-2.78	0.00	0.00
XLOC_058359	AC079781.7	chr7:97852117-97972357	5.92	0.86	-2.79	0.00	0.00
XLOC_011629	CHRM1	chr11:62891174-62921869	1.95	0.28	-2.79	0.00	0.00
XLOC_034032	C2orf40	chr2:106041549-106078159	3.34	0.48	-2.79	0.00	0.00
XLOC_052906	C5orf46	chr5:147850533-147906556	254.59	36.70	-2.79	0.00	0.00
XLOC_064156	FAM201A	chr9:38568683-38625132	2.11	0.30	-2.80	0.00	0.00
XLOC_010066	MYEOV	chr11:69292478-69367726	4.14	0.60	-2.80	0.00	0.00
XLOC_068091	PAK3	chrX:110944284-111227519	2.09	0.30	-2.80	0.00	0.00
XLOC_009086	TNNT3	chr11:1919561-1938779	2.03	0.29	-2.81	0.00	0.00
XLOC_025188	FA2H	chr16:74712550-74774831	7.30	1.03	-2.82	0.00	0.00
XLOC_055792	VWA7	chr6:31739947-31777403	6.87	0.97	-2.83	0.00	0.00
XLOC_064567	-	chr9:86750997-86768101	4.90	0.69	-2.83	0.00	0.00
XLOC_031985	ZNF208	chr19:21932957-22010949	1.06	0.15	-2.85	0.00	0.00

gene_id	gene	locus	NN	PP	log2(fold_change)	p_value	q_value
XLOC_003712	RP1-34M23.5	chr1:34712736-34859816	28.61	3.94	-2.86	0.00	0.00
XLOC_014678	-	chr12:24181038-24186766	1.77	0.24	-2.87	0.00	0.00
XLOC_049463	NPY1R	chr4:163323869-163352379	8.49	1.16	-2.87	0.00	0.00
XLOC_032114	SCGB1B2P	chr19:34576725-34577729	4.16	0.57	-2.88	0.00	0.01
XLOC_032799	ZSCAN18	chr19:58059238-58118427	17.97	2.41	-2.90	0.00	0.00
XLOC_052423	SLCO4C1	chr5:102233985-102296549	0.73	0.10	-2.90	0.00	0.01
XLOC_042151	SEC14L4	chr22:30475363-30505871	1.06	0.14	-2.91	0.00	0.00
XLOC_057316	CYP2W1	chr7:978140-1138271	5.39	0.72	-2.91	0.00	0.00
XLOC_011809	AP000439.3	chr11:69475365-69479989	2.90	0.38	-2.92	0.00	0.00
XLOC_058524	AC003989.4	chr7:110662503-111562517	0.78	0.10	-2.92	0.00	0.00
XLOC_064572	-	chr9:86942719-86947959	1.65	0.22	-2.93	0.00	0.00
XLOC_018633	ACOT1	chr14:73478483-73558947	9.69	1.26	-2.94	0.00	0.00
XLOC_014533	-	chr12:9852364-9932887	2.02	0.26	-2.96	0.00	0.01
XLOC_034000	SLC9A2	chr2:102619532-102712138	0.99	0.13	-2.97	0.00	0.00
XLOC_041333	UPB1	chr22:24270816-24528390	1.24	0.16	-2.97	0.00	0.00
XLOC_046069	-	chr3:182371771-182412296	0.94	0.12	-2.97	0.00	0.00
XLOC_060089	PDK4	chr7:95583498-95596491	7.31	0.92	-2.98	0.00	0.00
XLOC_030267	BEST2	chr19:12750068-12758458	1.59	0.20	-2.99	0.00	0.00
XLOC_028092	CHAD	chr17:50426157-50475211	2.21	0.28	-2.99	0.00	0.00
XLOC_004278	-	chr1:81134408-81178197	1.79	0.22	-3.00	0.00	0.00
XLOC_015353	TSPAN8	chr12:71124995-71586310	8.35	1.04	-3.01	0.00	0.00
XLOC_027692	RP1-56K13.5	chr17:39003247-39053210	1.10	0.13	-3.04	0.00	0.00
XLOC_048670	UGT2A1, UGT2A2	chr4:69588256-69653281	1.69	0.21	-3.04	0.00	0.00
XLOC_052949	PDE6A	chr5:149857938-149944845	0.93	0.11	-3.04	0.00	0.00
XLOC_049857	ANKRD33B	chr5:10564098-10658001	9.65	1.16	-3.06	0.00	0.00
XLOC_031384	ZNF135	chr19:58059238-58118427	1.75	0.21	-3.06	0.00	0.00
XLOC_038988	CDH4	chr20:61252425-61940746	1.05	0.13	-3.06	0.00	0.00
XLOC_024761	-	chr16:35315906-35336932	2.19	0.26	-3.07	0.00	0.00
XLOC_000627	RNU11, RP11-442N24__B.1	chr1:28589322-28649253	19.34	2.26	-3.10	0.00	0.00
XLOC_066617	CRAT	chr9:129092639-129149027	29.66	3.45	-3.10	0.00	0.00
XLOC_062575	PHYHIP	chr8:22219581-22232341	20.28	2.35	-3.11	0.00	0.00
XLOC_062636	SCARA5	chr8:27869759-27992727	32.28	3.74	-3.11	0.00	0.00
XLOC_061042	AC068587.2	chr8:12448012-12708364	4.60	0.53	-3.12	0.00	0.00
XLOC_049528	HAND2	chr4:173524968-173591324	1.62	0.18	-3.15	0.00	0.00
XLOC_051078	CTC-327F10.4	chr5:147850533-147906556	9.27	1.04	-3.15	0.00	0.00
XLOC_058354	TAC1	chr7:97731907-97740472	1.52	0.17	-3.16	0.00	0.00
XLOC_036915	-	chr2:121118654-121133457	1.14	0.13	-3.16	0.00	0.00

gene_id	gene	locus	NN	PP	log2(fold_change)	p_value	q_value
XLOC_010075	-	chr11:69639656-69640983	0.79	0.09	-3.16	0.00	0.00
XLOC_004794	PDZK1	chr1:145607989-145708148	2.49	0.28	-3.17	0.00	0.00
XLOC_039699	WFDC3	chr20:45747943-45811471	3.26	0.36	-3.18	0.00	0.00
XLOC_059928	AC005077.9, GTF2IP7	chr7:76088172-76108864	0.95	0.10	-3.19	0.00	0.00
XLOC_008837	SNRPGP6	chr10:116403890-116424625	0.88	0.10	-3.20	0.00	0.00
XLOC_013379	AC107016.2	chr12:52821256-52836899	116.97	12.57	-3.22	0.00	0.01
XLOC_051464	TPPP	chr5:612271-784746	13.50	1.43	-3.24	0.00	0.00
XLOC_040702	CLDN8	chr21:30214005-30216073	7.47	0.78	-3.26	0.00	0.00
XLOC_014682	-	chr12:24191516-24562741	1.87	0.19	-3.27	0.00	0.00
XLOC_011341	CHRM4	chr11:46380755-46391728	3.73	0.38	-3.28	0.00	0.00
XLOC_051587	RP11-54F2.1	chr5:10564098-10658001	4.78	0.48	-3.30	0.00	0.00
XLOC_065363	GLDC	chr9:6532463-6645698	3.37	0.34	-3.31	0.00	0.00
XLOC_015119	DCD	chr12:54644590-54648493	130.29	13.08	-3.32	0.00	0.00
XLOC_009850	FADS2, FEN1	chr11:61680380-61867411	254.19	25.41	-3.32	0.00	0.00
XLOC_007262	ACSM6	chr10:95194134-95231144	1.50	0.15	-3.33	0.00	0.00
XLOC_009866	SCGB2A2	chr11:62270148-62273159	42.99	4.26	-3.34	0.00	0.00
XLOC_014534	KLRF2	chr12:9852364-9932887	1.33	0.13	-3.36	0.00	0.00
XLOC_067741	AWAT1	chrX:70234602-70240979	6.61	0.63	-3.38	0.00	0.00
XLOC_031835	-	chr19:15594343-15608034	1.71	0.16	-3.43	0.00	0.00
XLOC_062401	CTA-398F10.2	chr8:8456908-8461382	3.38	0.31	-3.44	0.00	0.00
XLOC_060956	CLDN23	chr8:8701937-8704106	20.04	1.83	-3.45	0.00	0.00
XLOC_048360	PPARGC1A	chr4:23560922-23904089	3.04	0.28	-3.45	0.00	0.00
XLOC_058892	PIP	chr7:143132076-143139746	12.63	1.15	-3.46	0.00	0.00
XLOC_002688	HSD11B1	chr1:209661363-209806175	25.59	2.27	-3.49	0.00	0.00
XLOC_052908	CTC-327F10.5	chr5:147850533-147906556	1.53	0.14	-3.50	0.00	0.00
XLOC_033140	AC011752.1, AC067959.1	chr2:21221122-22531105	2.60	0.23	-3.50	0.00	0.00
XLOC_006907	AC022538.1	chr10:65570284-65820595	0.78	0.07	-3.55	0.00	0.00
XLOC_045395	HGD	chr3:120628172-120682788	1.29	0.11	-3.57	0.00	0.00
XLOC_006874	RP11-252C24.3	chr10:65110210-65145737	0.78	0.07	-3.57	0.00	0.00
XLOC_032818	AC016629.3	chr19:58559128-58599801	13.42	1.12	-3.58	0.00	0.00
XLOC_003331	-	chr1:11304778-11307565	1.53	0.13	-3.60	0.00	0.01
XLOC_030568	RP11-255H23.5	chr19:23738976-23874701	1.70	0.14	-3.61	0.00	0.00
XLOC_031961	CTD-2626G11.2	chr19:20746859-20755250	0.84	0.07	-3.62	0.00	0.00
XLOC_010074	-	chr11:69637578-69639538	0.95	0.08	-3.63	0.00	0.00
XLOC_006275	RP11-464C19.3	chr10:3911868-3949461	2.96	0.24	-3.64	0.00	0.00
XLOC_047811	NPY5R	chr4:163323869-163352379	1.20	0.10	-3.65	0.00	0.00
XLOC_013313	GPD1	chr12:50103621-50111443	6.53	0.52	-3.65	0.00	0.00

gene_id	gene	locus	NN	PP	log2(fold_change)	p_value	q_value
XLOC_042395	PNPLA5	chr22:43879504-43892013	3.67	0.29	-3.65	0.00	0.00
XLOC_013387	KRT18	chr12:52897186-52952901	11.34	0.89	-3.67	0.00	0.00
XLOC_042157	SEC14L6	chr22:30522571-30546881	19.35	1.52	-3.67	0.00	0.00
XLOC_011595	FADS1	chr11:61680380-61867411	36.43	2.86	-3.67	0.00	0.00
XLOC_066391	C9orf152	chr9:110190047-110208189	0.73	0.06	-3.68	0.00	0.00
XLOC_065203	RP11-98L5.5	chr9:135613944-135622022	4.05	0.31	-3.70	0.00	0.00
XLOC_067740	DGAT2L6	chrX:70133448-70206924	10.74	0.81	-3.73	0.00	0.00
XLOC_039390	RP5-1100I6.1	chr20:24085736-24148433	6.05	0.44	-3.78	0.00	0.00
XLOC_061852	KB-1410C5.1, KB-1410C5.2, KB-1410C5.3	chr8:101214128-101321611	4.60	0.33	-3.79	0.00	0.00
XLOC_054164	RP1-27K12.4	chr6:53497340-53617171	1.11	0.08	-3.80	0.00	0.00
XLOC_068352	CDR1-AS	chrX:140782404-140785393	19.87	1.42	-3.81	0.00	0.00
XLOC_020654	RP11-1008C21.1	chr15:37968354-38084775	2.53	0.18	-3.81	0.00	0.00
XLOC_053125	-	chr5:169410461-169426978	1.49	0.11	-3.82	0.00	0.00
XLOC_023558	MT4	chr16:56565048-56569284	17.45	1.22	-3.84	0.00	0.00
XLOC_009865	SCGB1D2	chr11:62208667-62260549	19.96	1.38	-3.85	0.00	0.00
XLOC_047101	OR7E94P, RP11-234K19.1	chr4:79585149-79601820	1.18	0.08	-3.87	0.00	0.00
XLOC_062910	-	chr8:58701761-58752513	2.81	0.19	-3.88	0.00	0.00
XLOC_044935	TNNC1	chr3:52451101-52454704	5.11	0.34	-3.89	0.00	0.00
XLOC_051546	C5orf49	chr5:7830377-7906025	1.12	0.07	-3.90	0.00	0.00
XLOC_006176	OR2T10, OR2T11	chr1:248449167-248642818	1.16	0.08	-3.91	0.00	0.00
XLOC_007355	ELOVL3	chr10:102226327-102229589	33.73	2.14	-3.98	0.00	0.00
XLOC_001649	HSD3B1	chr1:119507143-119515054	4.46	0.28	-3.98	0.00	0.00
XLOC_004005	RAB3B	chr1:51907952-51990764	2.73	0.17	-4.02	0.00	0.00
XLOC_039678	MATN4	chr20:45293444-45317824	9.30	0.56	-4.06	0.00	0.00
XLOC_051807	UGT3A2	chr5:36025630-36071358	7.94	0.46	-4.10	0.00	0.00
XLOC_059888	CLDN3	chr7:73768996-73770270	1.61	0.09	-4.15	0.00	0.00
XLOC_010053	GAL	chr11:68683646-68691180	53.24	2.93	-4.18	0.00	0.00
XLOC_037845	ERBB4	chr2:211375637-212538841	1.20	0.07	-4.19	0.00	0.00
XLOC_061529	TRIM55	chr8:66126895-66175487	0.70	0.04	-4.21	0.00	0.00
XLOC_036835	-	chr2:112882045-112985723	1.27	0.07	-4.22	0.00	0.00
XLOC_010861	H19,MIR675	chr11:1995162-2001470	91.12	4.68	-4.28	0.00	0.00
XLOC_010190	MOGAT2	chr11:75716920-75734943	2.17	0.11	-4.30	0.00	0.00
XLOC_015064	KRT77	chr12:52689625-52703516	164.78	7.82	-4.40	0.00	0.00
XLOC_052903	SPINK1	chr5:147824567-147831786	2.12	0.10	-4.42	0.00	0.01
XLOC_017013	GPR12	chr13:26755001-26760816	1.26	0.06	-4.43	0.00	0.00
XLOC_069154	AWAT2	chrX:70040536-70050065	16.23	0.68	-4.57	0.00	0.00
XLOC_048705	BTC	chr4:74738626-74795004	33.74	1.42	-4.58	0.00	0.00

gene_id	gene	locus	NN	PP	log2(fold_change)	p_value	q_value
XLOC_051672	CDH12	chr5:21616261-22853622	2.11	0.09	-4.58	0.00	0.00
XLOC_001643	HAO2	chr1:119368778-119394251	3.73	0.15	-4.59	0.00	0.00
XLOC_050778	RP11-257I8.2, RP11-395P13.7, RP11-508M8.1	chr5:125036680-125416647	1.24	0.05	-4.59	0.00	0.00
XLOC_003874	CFAP57	chr1:43164174-43274002	0.73	0.03	-4.60	0.00	0.00
XLOC_010221	THRSP	chr11:78015714-78080219	42.51	1.69	-4.65	0.00	0.00
XLOC_015283	WIF1	chr12:65017467-65121566	11.56	0.44	-4.72	0.00	0.00
XLOC_024895	RP11-26L20.3	chr16:55258674-55346844	0.79	0.03	-4.72	0.00	0.00
XLOC_022796	CACNA1H	chr16:1153240-1225257	8.90	0.33	-4.74	0.00	0.00
XLOC_035356	MOGAT1	chr2:222671676-222718719	1.07	0.04	-4.80	0.00	0.00
XLOC_033544	-	chr2:64486258-64595483	0.77	0.03	-4.80	0.00	0.00
XLOC_061381	RP11-350F16.1	chr8:46915188-46934446	2.05	0.07	-4.95	0.00	0.00
XLOC_028701	LINC01254	chr18:10405132-10425411	2.98	0.09	-5.02	0.00	0.00
XLOC_047873	LINC01612	chr4:170226604-170310849	1.28	0.04	-5.05	0.00	0.00
XLOC_000213	AADACL3	chr1:12716003-12741606	23.51	0.70	-5.07	0.00	0.00
XLOC_005836	-	chr1:221488748-221504463	1.12	0.03	-5.10	0.00	0.01
XLOC_040915	SLC37A1	chr21:42472485-42581440	0.76	0.02	-5.12	0.00	0.00
XLOC_065396	LINC00583, PES1P2	chr9:13881866-14021613	0.80	0.02	-5.15	0.00	0.00
XLOC_070008	ZNF736P6Y	chrY:8299691-8352143	3.13	0.08	-5.28	0.00	0.00
XLOC_015076	KRT8	chr12:52897186-52952901	30.77	0.74	-5.37	0.00	0.00
XLOC_060356	WNT2	chr7:117275387-117324884	1.28	0.02	-5.80	0.00	0.00
XLOC_059277	AGR3	chr7:16846712-16881990	4.52	0.07	-5.93	0.00	0.00
XLOC_001965	AC234582.1	chr1:155169407-155192916	2.40	0.03	-6.36	0.00	0.00
XLOC_069153	EDA	chrX:69616066-70039743	1.55	0.02	-6.41	0.00	0.00
XLOC_014963	RND1	chr12:48857144-48865898	0.14	4.83	5.07	0.00	0.00
XLOC_001402	-	chr1:95092489-95096479	2.04	0.02	-6.61	0.00	0.00
XLOC_030566	RP11-255H23.2	chr19:23738976-23874701	17.78	5.79	-1.62	0.00	0.00

Table S4. Divergent and convergent primers for the validation experiments

	**DIVERGENT Primers (5' – 3')**	
**circRNA name (circBase ID)**	**Forward**	**Reverse**
CDR1as (*hsa_circ_0001946*)	CATGTCTTCCAACGTCTCCA	CTGGAAGACCCGGAGTTGT
hsa_skin_088763 (*hsa_circ_0109237*)	AAATCATGCTGCTGAGAATCC	AATCTTCCATGCCCTGCTCT
hsa_skin_052271	CACCAGACATGCCCACTCT	TCCTGAGTACCCTTCACTGTCA
	**CONVERGENT Primers (5' – 3')**	
**circRNA name (circBase ID)**	**Forward**	**Reverse**
CDR1as (*hsa_circ_0001946*)	CCTGGAGGCCATTGGAAGAT	TCCAGTAACCTCCCAGTCTT
hsa_skin_088763 (*hsa_circ_0109237*)	AAAGCATTTTGCCAGCCCTC	TGCCACATGAGATTTGTTGGGTT
hsa_skin_052271	ACTGGAAGAAGGGGATCTGG	GTGGGCATGTCTGGTGGTAT
GAPDH	GAGTCCACTGGCGTCTTCA	GGGGTGCTAAGCAGTTGTT

Table S5. Associated genes of three validated circRNAs, *CDR1as*, *hsa_skin_088763* and *hsa_skin_052271*

r: Spearman correlation coefficient
p: p value

Associated genes of CDR1as via hsa-miR-7-5p											
gene	r	p	gene	r	p	gene	r	p	gene	r	p
HIP1	0.741	0.000	SRGAP2	0.616	0.000	IRS1	0.548	0.000	KLHL28	0.455	0.000
NFATC2	0.734	0.000	IRS2	0.616	0.000	GGT7	0.547	0.000	SNCA	0.455	0.000
NFIB	0.732	0.000	RYBP	0.612	0.000	PCGF5	0.546	0.000	RSBN1L	0.453	0.000
ERBB4	0.731	0.000	MAP1B	0.611	0.000	PRR13	0.541	0.000	WDR47	0.452	0.000
KMT2A	0.714	0.000	ZNF805	0.610	0.000	RYK	0.539	0.000	TDRP	0.451	0.000
ZBTB20	0.694	0.000	CDH20	0.609	0.000	PDE4D	0.538	0.000	FAM208A	0.444	0.000
GLI3	0.693	0.000	NFIA	0.608	0.000	DACH1	0.536	0.000	SEMA6D	0.439	0.000
GATAD2B	0.680	0.000	ARID2	0.606	0.000	TNRC6A	0.534	0.000	OXR1	0.436	0.000
GLG1	0.678	0.000	PBX3	0.605	0.000	COL1A2	0.533	0.000	HERPUD2	0.431	0.000
LIF	0.674	0.000	SOX6	0.603	0.000	PTAR1	0.533	0.000	TTC16	0.430	0.000
IDS	0.670	0.000	NOTCH3	0.603	0.000	PLCB1	0.529	0.000	OGT	0.428	0.000
ITGA9	0.667	0.000	PLEC	0.602	0.000	GRIN2A	0.528	0.000	ZMIZ1	0.425	0.000
WWP1	0.666	0.000	KIAA0430	0.600	0.000	MEGF9	0.527	0.000	FXR1	0.421	0.000
WNT2B	0.665	0.000	LIMD1	0.596	0.000	ZNF395	0.525	0.000	KIF16B	0.418	0.000
ARIH1	0.661	0.000	RBMS3	0.594	0.000	ZFAND4	0.521	0.000	KIF13A	0.417	0.000
KDM3B	0.660	0.000	CCDC80	0.593	0.000	NCOR1	0.520	0.000	FNDC4	0.413	0.000
PHF21A	0.659	0.000	SLC38A4	0.588	0.000	FAM46C	0.517	0.000	MOB1B	0.401	0.000
WDTC1	0.658	0.000	FLRT2	0.588	0.000	ATXN7	0.516	0.000	CAMK2D	0.398	0.000
TCF12	0.658	0.000	GSE1	0.584	0.000	FOXN3	0.513	0.000	TCF4	0.396	0.000
EGFR	0.657	0.000	TSNARE1	0.584	0.000	EGR3	0.508	0.000	CUL5	0.370	0.000
ZNF704	0.654	0.000	INSIG2	0.581	0.000	CTDSPL	0.508	0.000	KLF4	0.369	0.000
RNF24	0.649	0.000	COL4A3BP	0.580	0.000	WIPF2	0.507	0.000	TRIM33	0.369	0.000
NFIC	0.646	0.000	ARID4A	0.579	0.000	PIAS1	0.502	0.000	NDFIP2	0.368	0.000
RNF150	0.645	0.000	POU6F1	0.578	0.000	PPIL6	0.498	0.000	SLC38A2	0.365	0.000
IGF1R	0.645	0.000	SATB1	0.574	0.000	ORAI1	0.494	0.000	SLC16A7	0.365	0.000
LPP	0.645	0.000	ZBTB38	0.572	0.000	OSBPL5	0.493	0.000	GOLGB1	0.362	0.000
TGFBR2	0.636	0.000	ZNF652	0.568	0.000	RSBN1	0.493	0.000	PSEN1	0.355	0.000
PRKCB	0.636	0.000	MED13L	0.567	0.000	NLGN2	0.490	0.000	BMPR2	0.354	0.001
GATA6	0.635	0.000	AGO1	0.567	0.000	POGK	0.489	0.000	SH3GLB1	0.347	0.001
AKT3	0.635	0.000	PLAG1	0.562	0.000	KLF12	0.489	0.000	WDFY2	0.346	0.001
NFAT5	0.631	0.000	CRY2	0.556	0.000	ITGB8	0.489	0.000	ITCH	0.326	0.001
TAF1	0.626	0.000	EIF4EBP2	0.554	0.000	CHAMP1	0.485	0.000	CYTH3	0.318	0.002
ABI2	0.625	0.000	CDKL1	0.553	0.000	ZNF148	0.481	0.000	RNF141	0.316	0.002
SMG1	0.619	0.000	DPYSL2	0.552	0.000	ZBTB3	0.479	0.000	HBP1	0.305	0.003
SP1	0.619	0.000	PIK3CB	0.550	0.000	NKD1	0.469	0.000	CCNT2	0.293	0.004
NECAB1	0.618	0.000	CTDSP2	0.550	0.000	TAB2	0.468	0.000			
RAB5B	0.618	0.000	PARVA	0.549	0.000	CLASP2	0.468	0.000			
FAM168A	0.617	0.000	ZNF75A	0.549	0.000	GPBP1	0.456	0.000			

**Associated genes of CDR1as via hsa-miR-135b-5p**											
**gene**	**r**	**p**	**gene**	**r**	**p**	**gene**	**r**	**p**	**gene**	**r**	**p**
ERBB4	0.731	0.000	AKAP2	0.600	0.000	ZNF292	0.534	0.000	RSBN1L	0.453	0.000
LMX1B	0.719	0.000	PALM2-AKAP2	0.600	0.000	DIAPH2	0.533	0.000	SNRK	0.450	0.000
NAALADL2	0.710	0.000	ADNP	0.600	0.000	SMAD5	0.533	0.000	SV2B	0.440	0.000
DENND2A	0.708	0.000	PELI2	0.598	0.000	ELMSAN1	0.531	0.000	SEMA6D	0.439	0.000
SORL1	0.707	0.000	EBF1	0.598	0.000	SLC8A1	0.530	0.000	LMBRD2	0.438	0.000
TCF7L2	0.698	0.000	TXNIP	0.596	0.000	PLCB1	0.529	0.000	ZNF322	0.438	0.000
ZNF302	0.695	0.000	LIMD1	0.596	0.000	ARHGEF6	0.528	0.000	GAS7	0.437	0.000
ZNF862	0.687	0.000	DCLK1	0.595	0.000	MEGF9	0.527	0.000	NPTX1	0.436	0.000
NR3C2	0.684	0.000	KLF8	0.595	0.000	TNPO2	0.527	0.000	SUV420H2	0.435	0.000
USP31	0.682	0.000	THRA	0.594	0.000	ATP8A1	0.526	0.000	TLK1	0.434	0.000
HMBOX1	0.678	0.000	MMP16	0.593	0.000	FERMT2	0.525	0.000	NCOA1	0.431	0.000
TRPC1	0.676	0.000	FBXL16	0.591	0.000	FMN1	0.521	0.000	HERPUD2	0.431	0.000
TLN1	0.675	0.000	ZNF70	0.590	0.000	TRPC6	0.521	0.000	ZNF304	0.426	0.000
ZCCHC14	0.673	0.000	PRUNE2	0.590	0.000	LANCL1	0.520	0.000	SNX18	0.421	0.000
ZNF84	0.672	0.000	GSK3B	0.587	0.000	ARNT	0.518	0.000	APMAP	0.416	0.000
ZBTB44	0.670	0.000	SMARCA2	0.585	0.000	EFNB2	0.517	0.000	ZNF225	0.414	0.000
ARIH1	0.661	0.000	HDAC4	0.584	0.000	SNX16	0.516	0.000	GPM6B	0.414	0.000
SLC46A1	0.660	0.000	DIP2C	0.584	0.000	NCOA2	0.514	0.000	PANK3	0.413	0.000
KDM5A	0.659	0.000	GSE1	0.584	0.000	FOXN3	0.513	0.000	SLC35A1	0.410	0.000
ZNF135	0.657	0.000	KAT6B	0.583	0.000	REST	0.512	0.000	SIRT1	0.407	0.000
POU2F3	0.656	0.000	BBX	0.582	0.000	FANCF	0.511	0.000	CCSAP	0.405	0.000
TMEM136	0.656	0.000	DIDO1	0.582	0.000	RPS6KA6	0.511	0.000	RORA	0.405	0.000
DLG2	0.655	0.000	RASAL2	0.582	0.000	JDP2	0.511	0.000	ZCCHC3	0.403	0.000
ZNF704	0.654	0.000	VCAN	0.580	0.000	MDM4	0.510	0.000	SHOC2	0.403	0.000
RNF24	0.649	0.000	CTC-432M15.3	0.579	0.000	PSIP1	0.507	0.000	MOB1B	0.401	0.000
PTK2	0.646	0.000	RAPGEF6	0.579	0.000	CTTNBP2	0.507	0.000	RAB3GAP2	0.398	0.000
MSRB3	0.646	0.000	NEGR1	0.579	0.000	LONRF1	0.506	0.000	CAMK2D	0.398	0.000
MSL2	0.644	0.000	UTRN	0.576	0.000	RARA	0.504	0.000	OTUD3	0.396	0.000
IGSF10	0.642	0.000	ARHGAP6	0.574	0.000	LMLN	0.501	0.000	ARNTL	0.390	0.000
SKI	0.639	0.000	TBC1D5	0.574	0.000	CEP170	0.501	0.000	MEF2C	0.385	0.000
PTPRF	0.638	0.000	ARHGEF7	0.572	0.000	ZCCHC24	0.501	0.000	LATS2	0.384	0.000
GATA3	0.636	0.000	GCNT2	0.571	0.000	BMPR1A	0.498	0.000	PDE8B	0.380	0.000
TGFBR2	0.636	0.000	RARB	0.570	0.000	ADAMTS9	0.498	0.000	INTS2	0.379	0.000
AKT3	0.635	0.000	PRLR	0.569	0.000	ENTPD1	0.496	0.000	MED13	0.379	0.000
NF1	0.634	0.000	NPAT	0.569	0.000	BCL9L	0.494	0.000	AHSA2	0.377	0.000
PDGFA	0.631	0.000	ZNF652	0.568	0.000	PTPN3	0.493	0.000	TMEM106B	0.374	0.000
NFAT5	0.631	0.000	RBFOX2	0.567	0.000	CCDC50	0.493	0.000	ZBTB34	0.372	0.000
TBC1D4	0.628	0.000	AGO1	0.567	0.000	KIAA1324L	0.493	0.000	KLF4	0.369	0.000
ABAT	0.627	0.000	AGO3	0.567	0.000	CBLB	0.492	0.000	NDFIP2	0.368	0.000
ZKSCAN1	0.626	0.000	FAM110B	0.558	0.000	VAMP2	0.490	0.000	JAK2	0.362	0.000
ATP2B4	0.626	0.000	PHLPP2	0.556	0.000	POGK	0.489	0.000	ANGPTL2	0.357	0.000
SP8	0.626	0.000	C16orf52	0.556	0.000	FTO	0.487	0.000	SDCBP	0.355	0.000
ABI2	0.625	0.000	LDLRAD4	0.555	0.000	FOXK1	0.486	0.000	BMPR2	0.354	0.001
LRRN1	0.624	0.000	ANK3	0.554	0.000	SLC12A6	0.486	0.000	CPD	0.351	0.001
DTNA	0.622	0.000	ACVR1B	0.554	0.000	ASPH	0.485	0.000	ELK4	0.344	0.001

**Associated genes of CDR1as via hsa-miR-135b-5p**											
**gene**	**r**	**p**	**gene**	**r**	**p**	**gene**	**r**	**p**	**gene**	**r**	**p**
SP1	0.619	0.000	DAG1	0.553	0.000	TMEM237	0.484	0.000	GPR155	0.343	0.001
RAB5B	0.618	0.000	SLITRK6	0.552	0.000	TFAP2A	0.481	0.000	RASSF8	0.343	0.001
CCDC171	0.616	0.000	RNF217	0.551	0.000	KLHL15	0.481	0.000	MAN2A1	0.341	0.001
IRS2	0.616	0.000	APLF	0.550	0.000	SPOCK1	0.481	0.000	MRAS	0.340	0.001
RYBP	0.612	0.000	CTDSP2	0.550	0.000	KLF13	0.479	0.000	MID2	0.336	0.001
MYO9A	0.612	0.000	YBX2	0.549	0.000	BTBD2	0.475	0.000	ATG14	0.328	0.001
USP13	0.611	0.000	DCUN1D4	0.548	0.000	FZD1	0.474	0.000	CADM4	0.327	0.001
CEP85L	0.610	0.000	ALCAM	0.542	0.000	GDPD1	0.469	0.000	ERMP1	0.317	0.002
FOXO1	0.609	0.000	EVI5	0.542	0.000	MEF2A	0.462	0.000	SPRED1	0.310	0.002
FRMD4A	0.607	0.000	HIC2	0.539	0.000	KCTD12	0.462	0.000	ELOVL6	0.300	0.003
COL5A1	0.605	0.000	TET3	0.539	0.000	DST	0.461	0.000	PCMTD2	0.293	0.004
SERTAD2	0.604	0.000	CXCL12	0.539	0.000	TMEM168	0.460	0.000	MEGF11	0.289	0.005
CLASP1	0.603	0.000	C2CD2	0.539	0.000	TET2	0.459	0.000	GTF2A1	0.284	0.006
PLAGL1	0.603	0.000	TAF4	0.538	0.000	ETV3	0.459	0.000	CHSY1	0.277	0.007
RSF1	0.601	0.000	ANKRD40	0.538	0.000	MAPKBP1	0.457	0.000			
PSD3	0.600	0.000	BAG4	0.534	0.000	KLHL28	0.455	0.000			

**Associated genes of hsa_skin_088763 via hsa-miR-338-3p**											
**gene**	**r**	**p**	**gene**	**r**	**p**	**gene**	**r**	**p**	**gene**	**r**	**p**
SOX5	0.602	0.000	SLCO3A1	0.454	0.000	RBMS3	0.389	0.000	SS18L1	0.337	0.001
ATXN7L3B	0.531	0.000	CACNA2D1	0.445	0.000	FBXW7	0.387	0.000	CHL1	0.337	0.001
MACROD2	0.529	0.000	HELZ	0.445	0.000	TACC1	0.381	0.000	MAN2A1	0.333	0.001
MSL2	0.527	0.000	GATS	0.445	0.000	SOX4	0.379	0.000	ZSCAN12	0.331	0.001
WNT2B	0.518	0.000	ANAPC16	0.440	0.000	ARMCX3	0.379	0.000	TBC1D15	0.329	0.001
SOX6	0.515	0.000	SP6	0.440	0.000	KLHL12	0.378	0.000	SEMA6D	0.324	0.002
FOXP2	0.510	0.000	TMEM164	0.439	0.000	LMBRD2	0.377	0.000	FBXL3	0.321	0.002
TAF1	0.509	0.000	ZNF436	0.438	0.000	LGALSL	0.375	0.000	ERC1	0.321	0.002
NR3C2	0.507	0.000	JDP2	0.437	0.000	ATF2	0.374	0.000	NDFIP1	0.317	0.002
BRD3	0.506	0.000	EFNA5	0.435	0.000	SKIDA1	0.373	0.000	FAM168A	0.315	0.002
ZNF607	0.506	0.000	PCDHB16	0.433	0.000	LAMC1	0.372	0.000	DAB2IP	0.314	0.002
TMEM255A	0.505	0.000	NIPAL1	0.430	0.000	HUNK	0.370	0.000	TSHZ2	0.313	0.002
SIK2	0.501	0.000	KIAA1549L	0.426	0.000	PTN	0.368	0.000	PLD1	0.310	0.002
NHS	0.495	0.000	KIAA1429	0.426	0.000	SEPSECS	0.365	0.000	TRIM33	0.307	0.003
CDON	0.492	0.000	ZBTB10	0.425	0.000	CRELD1	0.365	0.000	DES	0.305	0.003
ZFP36L1	0.477	0.000	AKAP12	0.423	0.000	FRMD3	0.362	0.000	HPSE2	0.300	0.004
ZNF652	0.476	0.000	NOVA1	0.423	0.000	MAPT	0.362	0.000	HSPA12A	0.299	0.004
FBXO32	0.476	0.000	WNK1	0.419	0.000	NFIA	0.362	0.000	FGFR2	0.298	0.004
BBX	0.473	0.000	RNF150	0.415	0.000	VAV3	0.361	0.000	ESR2	0.293	0.004
ZBTB20	0.471	0.000	ABI2	0.413	0.000	SON	0.361	0.000	TANC1	0.290	0.005
DLG2	0.468	0.000	GATA6	0.411	0.000	AKT3	0.361	0.000	TBL1XR1	0.285	0.006
DMTN	0.462	0.000	TGOLN2	0.405	0.000	ZBTB18	0.354	0.001	GNG12	0.282	0.006
EIF4E3	0.462	0.000	JMJD1C	0.402	0.000	TIMP4	0.351	0.001	MAF	0.280	0.006
RAB30	0.460	0.000	ESYT2	0.395	0.000	SNX18	0.348	0.001	SEPT4	0.273	0.008
SLC35F5	0.459	0.000	TSPYL4	0.393	0.000	RNF141	0.346	0.001	TCF4	0.268	0.009
RNF217	0.456	0.000	GPD2	0.393	0.000	XRN1	0.343	0.001			
N4BP2L1	0.456	0.000	ABCA9	0.391	0.000	F10	0.343	0.001			
PEAK1	0.455	0.000	TSPYL5	0.390	0.000	MACC1	0.341	0.001			

**Associated genes of hsa_skin_088763 via hsa-miR-12a/b-3p**											
**gene**	**r**	**p**	**gene**	**r**	**p**	**gene**	**r**	**p**	**gene**	**r**	**p**
SOX5	0.602	0.000	UBN2	0.459	0.000	SEPT11	0.411	0.000	TET2	0.360	0.000
ERBB4	0.597	0.000	WDR31	0.459	0.000	NDFIP2	0.410	0.000	EGR3	0.356	0.000
PTPN14	0.583	0.000	C3orf52	0.458	0.000	LPAR1	0.410	0.000	ZBTB18	0.354	0.001
MAML2	0.553	0.000	BMPR1A	0.458	0.000	SMAD5	0.409	0.000	MGAT3	0.353	0.001
ZC3H6	0.545	0.000	SMURF2	0.457	0.000	CEP85L	0.408	0.000	MACF1	0.353	0.001
ZNF395	0.544	0.000	N4BP2L1	0.456	0.000	GCNT2	0.408	0.000	CASD1	0.352	0.001
NFIB	0.541	0.000	SALL2	0.454	0.000	DMXL1	0.407	0.000	MEIS1	0.351	0.001
ACSS3	0.540	0.000	SPRY1	0.453	0.000	ATXN7	0.407	0.000	MDFIC	0.350	0.001
BCL2	0.538	0.000	CYBRD1	0.452	0.000	ZCCHC2	0.404	0.000	DTNA	0.350	0.001
SATB1	0.536	0.000	TET3	0.450	0.000	PLAG1	0.404	0.000	CYB561D1	0.348	0.001
SSBP2	0.534	0.000	MAGI1	0.450	0.000	PARD6G	0.404	0.000	LASP1	0.347	0.001
USP31	0.534	0.000	SETD2	0.448	0.000	NCOA2	0.403	0.000	RNF141	0.346	0.001
ATXN7L3B	0.531	0.000	TGFBR2	0.448	0.000	NISCH	0.403	0.000	CSGALNACT2	0.345	0.001
RYBP	0.531	0.000	RNF38	0.446	0.000	AMER1	0.403	0.000	DIP2C	0.344	0.001
MTMR10	0.530	0.000	EPC1	0.446	0.000	NUAK1	0.403	0.000	AUTS2	0.343	0.001
MSL2	0.527	0.000	SLC2A4RG	0.445	0.000	MITF	0.402	0.000	CRISPLD1	0.343	0.001
SOX6	0.515	0.000	HELZ	0.445	0.000	TRIM24	0.402	0.000	MADD	0.342	0.001
MARCH6	0.514	0.000	CREBBP	0.443	0.000	JMJD1C	0.402	0.000	BTBD7	0.341	0.001
LPP	0.513	0.000	VCPIP1	0.443	0.000	POGZ	0.402	0.000	ARNT	0.341	0.001
JAZF1	0.512	0.000	ZNF292	0.441	0.000	KIAA1109	0.400	0.000	RBM12B	0.340	0.001
INPP5A	0.510	0.000	MET	0.440	0.000	IGSF10	0.398	0.000	PBRM1	0.340	0.001
FOXP2	0.510	0.000	ERBB2IP	0.440	0.000	FNIP1	0.397	0.000	TTLL7	0.340	0.001
RAI14	0.506	0.000	GABRB3	0.439	0.000	CHST15	0.397	0.000	MAP1B	0.339	0.001
PTK2B	0.506	0.000	ZNRF2	0.439	0.000	FAM73A	0.395	0.000	MPP2	0.339	0.001
AFF1	0.505	0.000	ARHGEF5	0.439	0.000	DACH1	0.394	0.000	TMEM168	0.339	0.001
TRDMT1	0.504	0.000	FGD4	0.437	0.000	SATB2	0.394	0.000	PRR13	0.338	0.001
NRXN3	0.504	0.000	TEAD1	0.435	0.000	NKAP	0.394	0.000	SS18L1	0.337	0.001
PDK4	0.502	0.000	EFNA5	0.435	0.000	GPBP1	0.394	0.000	CHL1	0.337	0.001
SIK2	0.501	0.000	PCDHA1	0.435	0.000	TSPYL4	0.393	0.000	AFF3	0.336	0.001
VKORC1L1	0.500	0.000	PCDHA10	0.435	0.000	INTU	0.393	0.000	FUCA1	0.336	0.001
WEE1	0.496	0.000	PCDHA11	0.435	0.000	GLCE	0.393	0.000	DAPK1	0.335	0.001
BACH2	0.496	0.000	PCDHA12	0.435	0.000	MAML1	0.393	0.000	ARNT2	0.335	0.001
TGFBR3	0.493	0.000	PCDHA13	0.435	0.000	EIF4EBP2	0.392	0.000	USP24	0.334	0.001
ZNF329	0.490	0.000	PCDHA2	0.435	0.000	SLC25A36	0.392	0.000	RSBN1L	0.334	0.001
PDGFA	0.490	0.000	PCDHA3	0.435	0.000	TRRAP	0.391	0.000	CELF2	0.330	0.001
LZTFL1	0.490	0.000	PCDHA4	0.435	0.000	RTF1	0.391	0.000	TBC1D15	0.329	0.001
ST7L	0.489	0.000	PCDHA5	0.435	0.000	HIC1	0.389	0.000	NUAK2	0.328	0.001
ELF2	0.489	0.000	PCDHA6	0.435	0.000	NEGR1	0.389	0.000	C5orf42	0.327	0.001
PNMA1	0.489	0.000	PCDHA7	0.435	0.000	CNOT2	0.388	0.000	CBFA2T3	0.327	0.001
ID4	0.487	0.000	PCDHA8	0.435	0.000	RUNX1T1	0.387	0.000	EBF1	0.326	0.001
CCND1	0.487	0.000	PCDHA9	0.435	0.000	PPM1K	0.387	0.000	FOXO4	0.324	0.002
PDCD4	0.486	0.000	PCDHAC1	0.435	0.000	FOXK1	0.385	0.000	SEMA6D	0.324	0.002

**Associated genes of hsa_skin_088763 via hsa-miR-12a/b-3p**											
**gene**	**r**	**p**	**gene**	**r**	**p**	**gene**	**r**	**p**	**gene**	**r**	**p**
PNRC1	0.485	0.000	PCDHAC2	0.435	0.000	FKBP7	0.385	0.000	PIK3CB	0.324	0.002
PPARGC1A	0.484	0.000	DCBLD2	0.435	0.000	ZMYM2	0.384	0.000	ELOVL3	0.323	0.002
RBL2	0.484	0.000	MICU3	0.435	0.000	CRTAP	0.383	0.000	LRP5	0.323	0.002
DNAJB4	0.484	0.000	VGLL3	0.433	0.000	FGF14	0.383	0.000	CTNND2	0.322	0.002
SP1	0.483	0.000	ZNF280C	0.433	0.000	TMOD2	0.381	0.000	VCAN	0.320	0.002
STXBP6	0.482	0.000	PGRMC2	0.433	0.000	ZC3H13	0.381	0.000	ARID3B	0.318	0.002
ISM1	0.482	0.000	JAK1	0.433	0.000	AGO3	0.380	0.000	GXYLT1	0.317	0.002
REPS2	0.480	0.000	SNX27	0.432	0.000	C8orf58	0.379	0.000	RXRG	0.317	0.002
KPNA5	0.480	0.000	FAM46A	0.430	0.000	CCSAP	0.378	0.000	AZIN1	0.317	0.002
ZKSCAN1	0.479	0.000	PIP4K2B	0.429	0.000	SPIN1	0.378	0.000	RBM27	0.316	0.002
ZNF420	0.476	0.000	USP30	0.429	0.000	SOGA3	0.377	0.000	DAB2IP	0.314	0.002
ZNF652	0.476	0.000	COL4A3BP	0.429	0.000	SPOCK1	0.376	0.000	TPST1	0.314	0.002
FBXO32	0.476	0.000	MAP3K1	0.428	0.000	CA2	0.376	0.000	SWT1	0.313	0.002
CBFA2T2	0.475	0.000	SPRY2	0.428	0.000	FAS	0.376	0.000	RAB8B	0.311	0.002
GREM2	0.475	0.000	TNRC6A	0.428	0.000	NT5DC3	0.376	0.000	SETX	0.311	0.002
ANKRD50	0.474	0.000	RPRD2	0.428	0.000	UBE2R2	0.376	0.000	HAS2	0.311	0.002
ZNF274	0.474	0.000	STAT5B	0.426	0.000	EPS15	0.375	0.000	CCDC82	0.306	0.003
EPN2	0.473	0.000	FAM46C	0.426	0.000	RORA	0.375	0.000	ZBTB2	0.306	0.003
BBX	0.473	0.000	VPS37D	0.425	0.000	FAM117B	0.375	0.000	PRKRIR	0.305	0.003
PAK3	0.472	0.000	MBTD1	0.424	0.000	LRCH1	0.373	0.000	SERINC3	0.300	0.003
ZNF667	0.470	0.000	ZBTB37	0.424	0.000	PURA	0.373	0.000	HSPA12A	0.299	0.004
MYO9A	0.470	0.000	PLA2R1	0.423	0.000	LSAMP	0.373	0.000	ZBTB34	0.297	0.004
CCDC171	0.470	0.000	AKAP12	0.423	0.000	SLC39A10	0.373	0.000	TGFB2	0.297	0.004
DOCK7	0.470	0.000	ZBED3	0.421	0.000	FTO	0.373	0.000	SRPK2	0.296	0.004
OSBPL8	0.469	0.000	GPC4	0.421	0.000	RSBN1	0.371	0.000	FKBP5	0.296	0.004
DLG2	0.468	0.000	WNK1	0.419	0.000	SLC7A6OS	0.371	0.000	PTGFR	0.295	0.004
KLHL28	0.468	0.000	PRELID2	0.419	0.000	CREBZF	0.371	0.000	SETD1B	0.293	0.004
FRA10AC1	0.468	0.000	KDM4A	0.419	0.000	ZNF423	0.371	0.000	USP46	0.292	0.004
GSK3B	0.467	0.000	XYLT1	0.419	0.000	CAMK2N1	0.371	0.000	ZEB1	0.291	0.005
PPP1R12A	0.466	0.000	MEF2A	0.417	0.000	PSD3	0.371	0.000	NCOA1	0.289	0.005
NFIX	0.466	0.000	RAPGEF2	0.417	0.000	TNRC6C	0.371	0.000	ORMDL1	0.289	0.005
KLHL15	0.465	0.000	ZFHX3	0.416	0.000	WASF2	0.370	0.000	CCDC6	0.288	0.005
FAM107B	0.464	0.000	EXOC8	0.416	0.000	ZNF225	0.370	0.000	TAX1BP1	0.283	0.006
EML5	0.463	0.000	FREM1	0.416	0.000	TMEM144	0.370	0.000	CFL2	0.281	0.006
EIF4E3	0.462	0.000	WIPF2	0.416	0.000	FZD4	0.368	0.000	TAB2	0.280	0.006
ZNF793	0.462	0.000	SLC8A1	0.415	0.000	SMAD3	0.368	0.000	MAF	0.280	0.006
TMEM136	0.462	0.000	ACVR2B	0.415	0.000	CEP128	0.367	0.000	SDCBP	0.277	0.007
ZBTB44	0.461	0.000	ADNP	0.415	0.000	LIPH	0.366	0.000	PKDCC	0.274	0.008
PRRC2B	0.461	0.000	RNF150	0.415	0.000	SEPSECS	0.365	0.000	ZBTB11	0.273	0.008
CNOT6L	0.460	0.000	PAPOLG	0.415	0.000	BAZ2B	0.365	0.000	SLC25A40	0.270	0.009
FNBP1L	0.460	0.000	TENM4	0.414	0.000	NFIA	0.362	0.000	ADAMTS5	0.270	0.009
LRIG1	0.460	0.000	GPC6	0.414	0.000	PLXNC1	0.361	0.000	IL17RD	0.269	0.009
MED13L	0.459	0.000	MRC2	0.414	0.000	TK2	0.361	0.000	MMGT1	0.266	0.010

**Associated genes of hsa_skin_052271 via hsa-miR-135b-5p**											
**gene**	**r**	**p**	**gene**	**r**	**p**	**gene**	**r**	**p**	**gene**	**r**	**p**
ALCAM	0.292	0.005	NAALADL2	0.280	0.007	SLC44A5	0.271	0.009			
**Associated genes of hsa_skin_052271 via hsa-miR-205-5p**											
**gene**	**r**	**p**	**gene**	**r**	**p**	**gene**	**r**	**p**	**gene**	**r**	**p**
MAGI2	0.288	0.0052	CEP128	0.283	0.01						
**Associated genes of hsa_skin_052271 via hsa-miR-27a-3p**											
**gene**	**r**	**p**	**gene**	**r**	**p**	**gene**	**r**	**p**	**gene**	**r**	**p**
GATA6	0.294	0.0042	CEP128	0.283	0.01	FOXP2	0.272	0.01	WNK1	0.269	0.00904118
CDR2	0.293	0.0044	KIAA1147	0.278	0.01	SLC38A4	0.271	0.01	PAN3	0.266	0.00994808
MAGI2	0.288	0.0052									

Table S6. CircRNAs significantly differentially expressed between PP and NN (p-value < 0.01)

Avg PP: average expression level in PP
Avg NN: average expression level in NN
log2(PP/NN): log2 fold change of PP vs. NN
p-value: p value

name	Avg PP	Avg NN	log2(PP/NN)	p-value
hsa_skin_176158	20.63	59.07	-1.52	0.005
hsa_skin_017868	24.75	84.19	-1.77	0.005
hsa_skin_173421	15.71	42.25	-1.43	0.004
hsa_skin_102568	1.83	7.55	-2.05	0.005
hsa_skin_137810	2.98	21.99	-2.88	0.000
hsa_skin_028220	4.05	23.72	-2.55	0.002
hsa_skin_050166	15.69	42.72	-1.44	0.006
hsa_skin_039030	13.12	103.15	-2.97	0.000
hsa_skin_032293	9.80	14.89	-0.60	0.000
hsa_skin_006010	7.48	30.19	-2.01	0.001
hsa_skin_112375	3.54	16.56	-2.22	0.001
hsa_skin_150608	3.26	14.96	-2.20	0.000
hsa_skin_189139	4.05	17.35	-2.10	0.005
hsa_skin_192510	92.49	275.52	-1.57	0.005
hsa_skin_223866	3.17	34.37	-3.44	0.000
hsa_skin_059513	4.13	12.13	-1.56	0.003
hsa_skin_013327	17.45	44.27	-1.34	0.005
hsa_skin_130995	16.07	54.42	-1.76	0.008
hsa_skin_194732	2.47	20.78	-3.07	0.000
hsa_skin_194345	23.57	310.97	-3.72	0.000
hsa_skin_052271	1.43	9.92	-2.79	0.000
hsa_skin_194228	6.83	26.73	-1.97	0.000
hsa_skin_093700	1.43	11.77	-3.04	0.000
hsa_skin_098241	15.60	25.64	-0.72	0.000
hsa_skin_017093	2.85	14.16	-2.31	0.004
hsa_skin_088763	21.83	129.55	-2.57	0.000
hsa_skin_143837	1.46	14.89	-3.35	0.001
hsa_skin_142248	100.02	50.76	0.98	0.000
hsa_skin_169953	30.30	37.90	-0.32	0.010
hsa_skin_146560	27.17	12.48	1.12	0.000
hsa_skin_214394	29.05	20.63	0.49	0.003
hsa_skin_232176	36.79	14.87	1.31	0.000
hsa_skin_026246	40.16	30.11	0.42	0.001
hsa_skin_153897	77.69	33.06	1.23	0.000
hsa_skin_183503	181.70	141.33	0.36	0.005
hsa_skin_216799	37.60	6.77	2.47	0.000

name	Avg PP	Avg NN	log2(PP/NN)	p-value
hsa_skin_199788	53.33	14.32	1.90	0.000
hsa_skin_213436	25.78	12.18	1.08	0.000
hsa_skin_192421	71.48	28.28	1.34	0.000
hsa_skin_203265	61.75	24.55	1.33	0.000
hsa_skin_083378	27.21	17.12	0.67	0.000
hsa_skin_050529	204.16	144.49	0.50	0.006
hsa_skin_083065	680.04	415.55	0.71	0.001
hsa_skin_189369	18.41	11.27	0.71	0.002
hsa_skin_171562	37.36	17.79	1.07	0.000
hsa_skin_156445	8.79	2.43	1.85	0.000
hsa_skin_228653	5.71	1.82	1.65	0.000
hsa_skin_006690	13.62	11.38	0.26	0.008
hsa_skin_228518	10.72	5.15	1.06	0.002
hsa_skin_060631	12.85	4.19	1.62	0.002
hsa_skin_130118	12.98	9.89	0.39	0.001
